# Whole genome resequencing in tomato reveals variation associated with introgression and breeding events

**DOI:** 10.1186/1471-2164-14-791

**Published:** 2013-11-14

**Authors:** Mathilde Causse, Nelly Desplat, Laura Pascual, Marie-Christine Le Paslier, Christopher Sauvage, Guillaume Bauchet, Aurélie Bérard, Rémi Bounon, Maria Tchoumakov, Dominique Brunel, Jean-Paul Bouchet

**Affiliations:** INRA, UR1052, Génétique et Amélioration des Fruits et Légumes, BP94, Montfavet, F-84143 France; US1279 INRA, Etude du Polymorphisme des Génomes Végétaux, CEA-Institut de Génomique-Centre National de Génotypage, Evry, 91057 France; Syngenta Seeds 12, chemin de l’Hobit, Saint-Sauveur, 31790 France; BIOGEMMA, Centre de Recherche de Chappes, CS 90126, Chappes, 63720 France

**Keywords:** Tomato, Genome, Sequence, Single nucleotide polymorphism, Introgression

## Abstract

**Background:**

One of the goals of genomics is to identify the genetic loci responsible for variation in phenotypic traits. The completion of the tomato genome sequence and recent advances in DNA sequencing technology allow for in-depth characterization of genetic variation present in the tomato genome. Like many self-pollinated crops, cultivated tomato accessions show a low molecular but high phenotypic diversity. Here we describe the whole-genome resequencing of eight accessions (four cherry-type and four large fruited lines) chosen to represent a large range of intra-specific variability and the identification and annotation of novel polymorphisms.

**Results:**

The eight genomes were sequenced using the GAII Illumina platform. Comparison of the sequences with the reference genome yielded more than 4 million single nucleotide polymorphisms (SNPs). This number varied from 80,000 to 1.5 million according to the accessions. Almost 128,000 InDels were detected. The distribution of SNPs and InDels across and within chromosomes was highly heterogeneous revealing introgressions from wild species and the mosaic structure of the genomes of the cherry tomato accessions. In-depth annotation of the polymorphisms identified more than 16,000 unique non-synonymous SNPs. In addition 1,686 putative copy-number variations (CNVs) were identified.

**Conclusions:**

This study represents the first whole genome resequencing experiment in cultivated tomato. Substantial genetic differences exist between the sequenced tomato accessions and the reference sequence. The heterogeneous distribution of the polymorphisms may be related to introgressions that occurred during domestication or breeding. The annotated SNPs, InDels and CNVs identified in this resequencing study will serve as useful genetic tools, and as candidate polymorphisms in the search for phenotype-altering DNA variations.

**Electronic supplementary material:**

The online version of this article (doi:10.1186/1471-2164-14-791) contains supplementary material, which is available to authorized users.

## Background

Currently next generation sequencing facilitates SNP discovery and allows deeper analysis of genome variation [1, 2]. In plants, SNP discovery has been performed either from RNA-Seq experiments [3, 4] or whole genome resequencing. Millions of polymorphisms have thus been discovered in Arabidopsis [5], rice [6, 7], soybean [8] and maize [9, 10].

The tomato genome has recently been sequenced and the international Tomato Genome Consortium has released a high-quality reference sequence [11]. The available sequence covers 780 Mb of the estimated 900 Mb. The annotation predicts 34,724 gene models, among which 30,855 were confirmed by RNA-Seq data. An initial comparison of the genomes of the sequenced cultivated accession (*Solanum lycopersicum*) and an accession of the closest wild relative, *S. pimpinellifolium*, revealed more than 5.4 million SNPs representing a divergence of 0.6%.

Tomato is a model species for fruit development and composition and is also a vegetable of high economic importance. It is grown all over the world, and its production has continuously increased over the last 50 years. Tomato originated in South America where all the wild species related to cultivated tomato grow in the Andean region. Domestication probably started in Peru or Ecuador followed by diversification in Mexico or alternatively domestication directly took place in Mexico [12]. Tomato evolved following several bottlenecks that considerably reduced the molecular diversity of the cultivated accessions. This hypothesis is supported by the very low polymorphism rate observed in cultivated species compared to wild relatives [13, 14], but also when analyzing diversity profiles of cherry-type tomato accessions (*S. lycopersicum* cv*. cerasiforme)*, which are intermediate between wild and modern cultivated accessions [15, 16]. In contrast, tomato breeding has led to a wide range of phenotypic adaptations to different environments and different phenotypes for fruit shape, size and color [17]. This was mainly due to introgressions from the related wild species and the discovery of major mutations [18].

As a genetic model for fruit crops, tomato has been used in many QTL mapping and gene cloning studies. Due to the lack of molecular polymorphism, most of the gene and QTL mapping experiments were performed on inter-specific progeny involving a cultivated and a wild species [19]. The use of wild relatives has allowed the discovery of several useful genes and QTLs [20, 21]. Since the first studies of tomato molecular diversity and gene mapping, molecular markers have evolved from RFLP [22] to AFLP [23], then SSR [24] and later SNP. SNPs were first discovered through *in silico* mining of EST [25–27] and amplicon sequencing of conserved ortholog sequences in different varieties [16, 28, 29]. Recently a large EST sequencing effort allowed the building of an Infinium array carrying ≈ 8500 SNPs [30–32].

In this article we present the polymorphisms detected from the resequencing of eight tomato accessions chosen to represent a large range of intraspecific variation. While characterizing the diversity of 360 tomato accessions with 20 SSR and later 275 SNPs, we developed nested core collections representing a maximum of molecular and phenotypic variation [15]. In order to discover SNPs and analyze the distribution of polymorphisms in the tomato genome, we have re-sequenced the whole genomes of eight lines corresponding to the smallest core collection composed of four cherry-type and four cultivated accessions. The genome sequences were then aligned to the reference genome sequence and alignments were screened for SNPs. The distribution and characteristics of the polymorphisms is presented. A set of SNPs was cross validated with results from a genotyping array. The distribution of polymorphisms between accessions and chromosomes is discussed in regard to the recent diversification of tomato.

## Results

We analysed two groups of accessions: a group of four cherry-type tomato accessions whose genomes consist in an admixture between the genomes of *S. lycopersicum* and *S pimpinellifolium*[16] and a group of four large-fruited lines typical of the cultivated accessions or breeding lines used 1950 and 1970. The eight lines were chosen to maximise the molecular diversity detected with 20 SSR markers in a collection of 360 tomato accessions [15]. Following Sanger sequencing of 81 amplicons in 90 accessions (*S. pimpinellifolium,* cherry and cultivated accessions), we showed that 76% of the 275 SNPs identified in the collection were detected in at least one of these eight lines [16]. Furthermore, the 66 SNPs that were not polymorphic among the eight lines were only polymorphic in *S. pimpinellifolium* accessions. We can thus predict that a large fraction of the SNPs present in any accession of the cultivated species were detected in this sample.

### Genome sequencing

Genome sequencing of the eight tomato lines yielded 970 million reads, most of them being 101 bp paired-end reads. After cleaning, 82 to 90% of the reads remained and were mapped to the high-quality genomic reference sequence of Heinz 1706 [11]. A total of 95.4 to 98.8% of the reads mapped onto the genome, depending on the lines. The reads covered 89 to 92% of the reference genome sequence. The average sequence depth of coverage varied from 6.7x to 16.6x depending on the accession, with the average being 11.2x (Table 1).

**Table 1:** **Total number of reads sequenced and mapped onto the Heinz 1706 reference genome after resequencing eight tomato accessions using Illumina Genome Analyser**

Accession	Cervil	Plovdiv	LA1420	Criollo	Stupicke	Ferum	Levovil	LA0147
Nb reads (million)	149.2	124.7	121.9	84.3	123.7	88.4	69.2	208.4
Nb nucleotides (Gigabases)	15.1	12.6	12.3	8.5	12.5	8.9	7.0	20.2
Depth	19.6	16.5	16.2	11.1	16.4	11.7	9.2	26.5
% sequences after cleaning	85.3	87.3	90.1	89.6	88.3	87.5	88.2	81.8
Depth after cleaning	13.3	12.2	12.5	8.1	12.0	8.2	6.7	16.6
% sequences mapped	95.4	97.1	97.1	98.2	98.2	98.5	95.9	98.8
% coverage (depth = 4)	88.8	88.6	88.9	81.1	90.5	82.2	72.7	92.3

**Figure 1:**
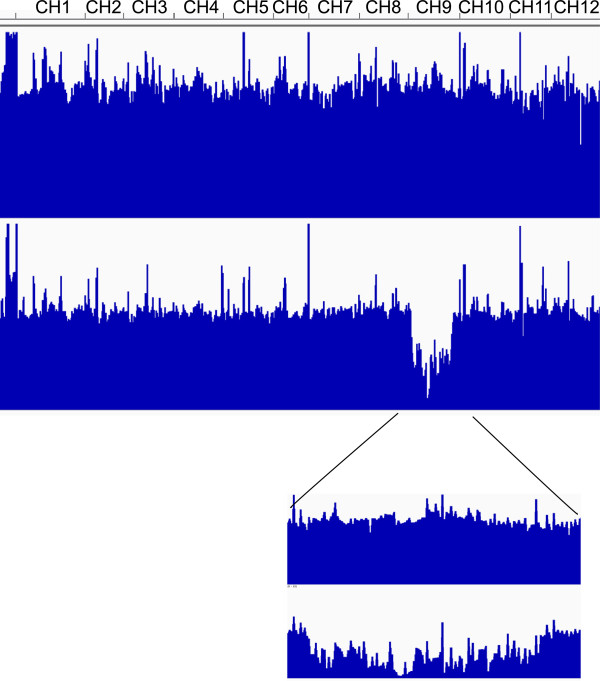
**Genome View of the whole genome sequences (top) and zoom on chromosome 9 (bottom) of two lines (Stupicke top, Levovil Bottom).** The high peaks correspond to sequences with high homology with organelle genomes. The chromosome 9 of Levovil corresponds to the introgression from a wild related species (image obtained with Integrative Genome Viewer IGV software; [52]).

Genome coverage was equivalent for all accessions and chromosomes except for one long region of chromosome 9 from the Levovil accession, which corresponded to an introgression from a distant species (Figure 1). The depth of coverage was also quite similar except for the peaks corresponding to regions with high homology with organelle genomes (predicted from the reference genome [11]). To avoid contamination with chloroplastic and mitochondrial DNA reads, all the reads showing a depth higher than 128x were removed from subsequent analysis, as performed elsewhere [7].

### Polymorphisms in the eight lines

A total of 4,290,679 unique SNPs and 127,913 InDels were detected when comparing each genome separately to the reference sequence, with the parameters defined in the Materials and Methods. For detecting homozygous polymorphisms, we applied two filters: a minimum of 4 reads and a maximum of 128 reads had to be mapped at any position and a minimum allele frequency of 0.9 was required. If we increased the minimal depth to 8, the number of SNPs dropped to 3,173,618 but several polymorphisms previously detected by Sanger sequencing were no longer detected, in particular in the three lines with a depth of coverage lower than 10x (Levovil, Ferum and Criollo).

**Figure 2:**
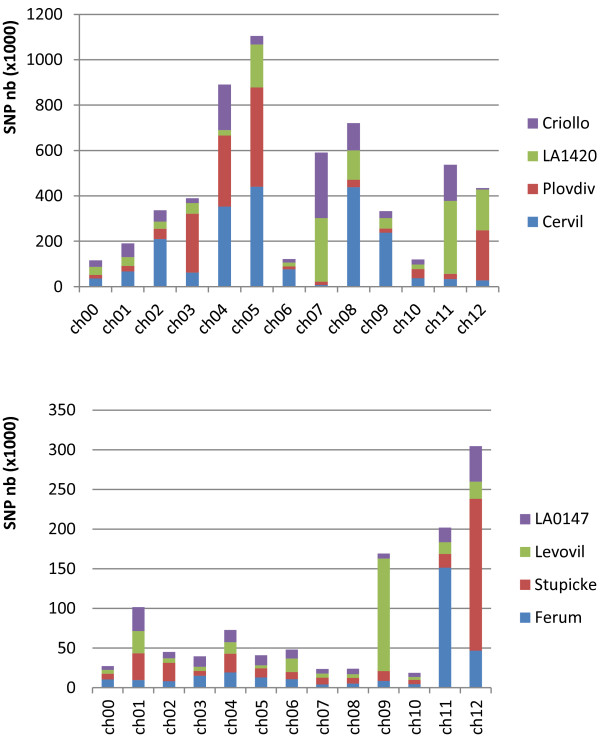
**Distribution of the numbers of homozygous SNPs detected per chromosome and line for the four** ***S. l. cerasiforme*** **lines, Cervil, Plovdiv, LA 1420 and Criollo (top) and the four** ***S. lycopersicum*** **large fruited lines, Stupicke, Ferum, Levovil and LA 0147 (bottom).**

The total number of SNPs varied widely from one line to another, with a range of one to two million in the four *S. l. cerasiforme* accessions and from 180,000 to 350,000 in the four *S. lycopersicum* lines (Additional file 1). The total number of SNPs also varied widely between the different chromosomes (Figure 2). Chromosomes 4, 5, 7, 8, 9, and 11 carried the highest number of SNPs (more than 350,000 unique SNPs per chromosome) and very few SNPs were detected on chromosomes 1, 6, and 10 (less than 150,000 unique SNPs). The range of variation between the chromosomes reached 10-fold on average and 61-fold for the accession the most distant from the reference (Cervil).

**Figure 3:**
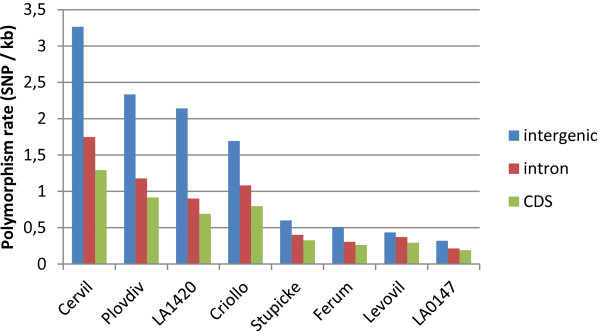
**Distribution of the polymorphism rate (π) in intergenic regions, introns and coding sequences (CDS) in the four** ***S. l. cerasiforme*** **type lines and four** ***S. lycopersicum*** **large fruited lines.**

The nucleotide diversity π (average number of SNPs per nucleotide) varied among the lines from 2.49×10^-4^ to 2.81×10^-3^. In introns, these values ranged from 2.14×10^-4^ (for LA 0147) to 1.75×10^-3^ (for Cervil) and in the coding sequences from 1.90×10^-4^ to 1.29×10^-3^ for the same lines (Figure 3). It also varied from one chromosome to another, with chromosome 10 showing the lowest value (9.80×10^-4^ on average for the eight accessions) and chromosome 5 the highest (9.5×10^-3^). The range of variation in π among the lines was higher than 100-fold for chromosome 5 while it was lower than 10-fold for chromosomes 1 and 6. Within the lines, the range varied from 8-fold for LA 0147 to 63-fold for Cervil.

The contribution of each line to the overall number of SNPs was also highly variable. For instance, for the four *S.l. cerasiforme* accessions, more than 75% of the SNPs detected in Cervil were on chromosomes 2, 4, 5, 8 and 9, while chromosomes 4 and 7 contributed to more than half of the SNPs of Criollo. The *S. lycopersicum* Levovil accession presented an excess of SNPs on chromosome 9 (52% of the SNPs for this accession were found on this chromosome), while this was evident on chromosome 11 for Ferum (50% of the SNPs) and on chromosome 12 for Stupicke (53% of the SNPs).

**Figure 4:**
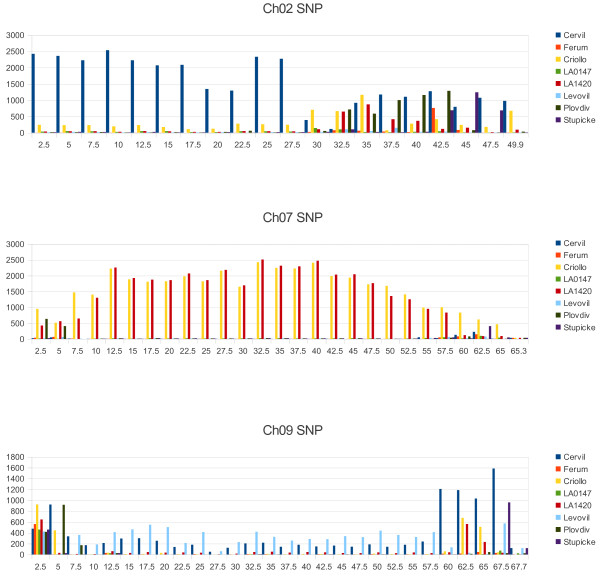
**Distribution of the number of homozygous SNP along the chromosomes 2, 7, and 9 (using a window size of 2.5 Mbp).**

The distribution of the SNPs along each chromosome also showed high variation as illustrated in Figure 4 and Additional file 2 for every chromosome. In general, SNPs were more frequent in the distal parts of chromosomes, which correspond to regions with higher recombination frequency [33] and gene density [11]. Nevertheless some lines also exhibited large number of SNPs (more than 1000 SNPs/Mb) in long regions covering the centromeric region such as on chromosomes 2, 4, 5, 8 and 9 for Cervil, on chromosome 3, 4, 5 and 12 for Plovdiv, on chromosome 5, 7, 8, 11 and 12 for LA 1420 and on chromosome 4, 7, 8 and 11 for Criollo. In the four large fruited lines, such patterns concerned only chromosome 9 for Levovil, 11 for Ferum and 12 for Stupicke. In these lines, a large number of regions were very poor in SNPs (less than 50 SNP/Mb in 93 regions of one megabase). SNP number did not appear to be related to the physical size of the chromosomes. Only 140,000 SNPs were discovered on the longest chromosome (chromosome 1, 90 Mb), while more than 600,000 were detected on chromosomes 4, 5 and 8, covering each around 60 Mb.

### Validation of SNPs with the Infinium SNP array

In order to validate the SNPs detected, we compared the genotypes obtained from the SolCAP SNP array for 7720 SNPs [32] with the SNPs we detected for six of the eight lines. We detected 7430 SNPs (96.2%) that matched perfectly. Among the 290 differences observed between our prediction and the SolCAP genotyping, 43 were different in every line and 166 just in one. Nevertheless 78% of the observed discrepancies were genotyped as heterozygous on the array and may thus correspond to a genotyping error on the array. If we do not take into account the heterozygous SNPs and those that were identical in every line, the rate of discrepancy dropped to below 1%.

### Detection of InDels

**Figure 5:**
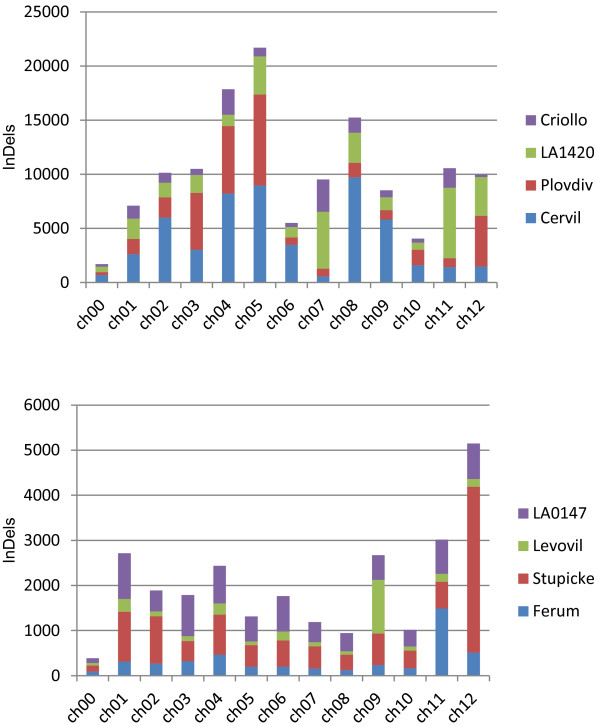
**Distribution of the number of InDels detected per chromosome and line for the four S. l. cerasiforme lines (top) and the four S. lycopersicum lines (bottom).**

A total of 127,913 unique InDels were detected in the eight lines compared to the reference genome. This number varied from 13,898 to 53,222 in cherry tomato lines and from 2,894 to 10,886 in *S. lycopersicum* lines (Additional files 2 and 3). Their distribution across chromosomes was more homogeneous than for SNPs, although a few chromosomes with a high density compared to the average could be detected (chromosome 4, 5, 7 and 8 in the cherry-type accessions and chromosomes 9, 11, and 12 for the cultivated tomatoes, Figure 5). In most cases, the chromosomes carrying a high number of SNPs also exhibited a high number of InDels. The correlation between SNP and InDel numbers on the 12 chromosomes was higher than 0.98 for all lines except for LA 0147 (r = 0.64). The frequency of InDels varied on average from one per 14 kb for Cervil to one per 270 kb for Levovil. At the chromosome level, these values ranged from one indel per 6.4 kb to 717 kb. The majority of InDels corresponded to a unique base modification, but a maximum of 32 bp deletions and 25 bp insertions were detected. The number of insertions was a little higher than the number of deletions (with a ratio varying from 1.05 to 1.35) according to the lines.

### Heterozygous SNPs

Tomato is an autogamous crop and the sequenced accessions were maintained by controlled self pollination. We thus expected a very low rate of residual heterozygosity. An SNP was declared heterozygous when the frequency of both alleles was comprised between 0.4 and 0.6. The total number of unique heterozygous SNPs was 314,560 (Additional file 4). The distribution of heterozygous SNPs was much more homogeneous across lines and chromosomes than the distribution of homozygous SNPs (Additional file 5). The heterozygous SNPs corresponded to a variable fraction of the total SNPs (from 8% for Cervil to 27% for Levovil). A large part of the heterozygous SNPs (14.6%) were assigned to chromosome 0 (corresponding to the sequences which could not be assigned to any of the 12 chromosomes due to the lack of genetic markers [11]) which represents only 2.7% of the reference genome and carries a large amount of repeated sequences. We could hardly identify any chromosome fragment in any line which could represent residual heterozygosity covering several hundreds of kb. This suggested that a large part of the heterozygous SNPs could result from mapping paralog sequences rather than revealing actual residual heterozygozity.

### SNP annotation

Among the SNPs, 57% were in intergenic regions, 34% in upstream or downstream regions of a gene, 5% were intronic and 3.4% in coding sequences. The effect of each SNP was classified according to SNPeff V2.1b software [34] into four classes (1) “modifier”, for the SNPs located outside the genes, in non transcribed regions or in introns, (2) “low effect” for variants in coding regions which do not change the amino acid sequence, (3) “moderate” effect for variants which change the amino acid sequence and (4) “high effect” for variants which modify splice sites, stop or start codons (loss or gain). Table 2 shows the proportion of variants in each class. More than 98% of the SNPs were classified as modifiers. The fraction of moderate variants ranged from 0.93 to 1.5% according to the accessions and the low effect from 0.80 to 1.3%. The high effect variants represented the smallest class, with 184 to 937 SNPs depending on the line.

**Table 2:** **Distribution of the SNP effect per type of effect in the four cherry-type (** ***S. l. cera*** **) and four** ***S. lycopersicum*** **(S. lyc) lines**

		***S. l. cera***	***S. l. cera***	***S. l. cera***	***S. l. cera***	***S. lyc***	***S. lyc***	***S. lyc***	***S. lyc***
Accession		Cervil	Plovdiv	LA 1420	Criollo	Stupicke	Ferum	Levovil	LA 0147
**High effect**	**Total**	**937**	**701**	**648**	**572**	**273**	**240**	**220**	**184**
	Splice site acceptor	109	68	63	50	27	23	25	19
	Splice site donor	104	70	57	64	18	27	17	14
	Stop gained	409	328	294	244	106	76	69	65
	Stop lost	214	169	170	156	86	81	82	65
	Start lost	101	66	64	58	36	33	27	21
**Moderate effect**	**Total**	**25,632**	**18,390**	**14,333**	**15,713**	**6,678**	**5,504**	**5,915**	**4,101**
Non synonymous coding									
**Low effect**	**Total**	**19,681**	**13,698**	**9,700**	**12,244**	**4,750**	**3,624**	**4,304**	**2,537**
	Non synonymous start	18	13	6	17	4	4	6	4
	Start gained	388	198	183	263	80	39	77	40
	Synonymous coding	19,218	13,448	9,481	11,924	4,654	3,564	4,208	2,485
	Synonymous stop	57	39	30	40	12	17	13	8
**Modifier**	**Total**	**2,349,654**	**1,669,629**	**1,516,290**	**1,231,706**	**439,151**	**366,977**	**323,588**	**233,987**
	Downstream	395,731	269,917	214,303	226,489	92,087	70,696	63,259	54,897
	Intergenic	1,377,524	1,019,498	1,000,788	676,540	215,862	200,063	164,291	102,012
	Intragenic	26,634	14,937	10,911	15,335	4,212	2,974	5,292	2,319
	Intron	118,329	79,698	60,972	73,234	27,095	20,573	25,094	14,524
	Upstream	422,540	280,576	225,411	234,350	95,053	71,642	63,800	59,194
	UTR 5 Prime	2,540	1,371	1,110	1,551	507	280	1,383	305
	UTR 3 Prime	6,347	3,632	2,795	4,207	1,335	749	469	736

SNPs were annotated using SNPeff onto the SL2.40 reference genome.

**Figure 6:**
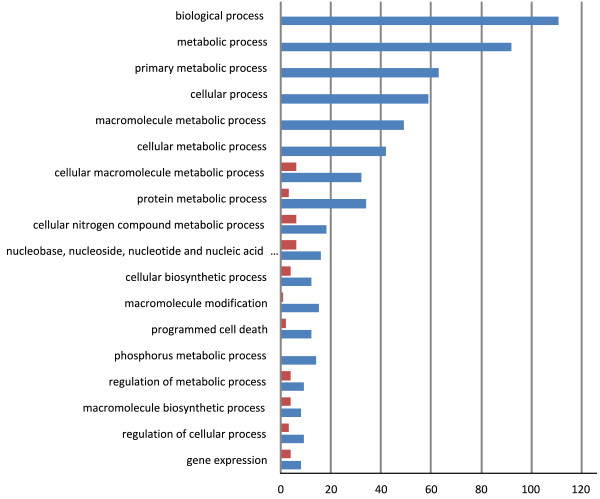
**Distribution of the Gene Ontology of the genes for which SNP with High effect were detected in Cervil (blue, 603 genes) and Levovil (red, 43 genes) tomato lines.**

Among the SNPs detected in coding sequences, 40% led to synonymous amino acid changes, 56% to non synonymous amino acid changes, with 1.7% causing a start or stop loss or gain, 0.4% a change in splice site, 0.1% a stop in the coding sequence and 0.04% a non synonymous start. The percentage of InDel with high effects (0.7%) was higher than for SNPs (0.097%) as an InDel may rapidly cause a frame shift in the sequence (Additional file 6). The SNPs with a high effect impacted 1779 genes. GO annotation of these genes revealed an excess of genes related to apoptosis and tRNA processing. The SNPs with moderate (non synonymous) effects impacted 18,154 genes, corresponding to several functions, with an excess of GO categories related to stress responses. The distribution into functional category of the genes subjected to high effect modifications were quite different for the eight lines, as illustrated in Figure 6 for two distant lines. The genes affected in the lines that are the closest to the reference sequence were mostly related to regulatory processes while in Cervil, the most distant line, they were involved in all categories.

### Copy Number Variant (CNV) identification

Structural variations were detected in the genomes of the five lines with coverage higher than 10x by a global analysis of the read depth variation in 2000 bp-windows. The comparison of read depth along the chromosomes revealed at least 1686 regions where a significant variation in depth in at least one line suggested a CNV. A maximum number of CNV was detected for Cervil (with 641 regions showing a significant lower depth and 234 regions a higher depth (Additional file 7). In contrast, LA 0147 showed an excess of regions with higher depth than the average (416 regions with excess and 125 with default). On average, 527 of the 1686 regions matched with a gene region, and in total 1235 genes were impacted. A significant excess of genes corresponding to cell death processes were detected.

## Discussion

Several experiments have identified SNPs in tomato. A few thousand SNPs have been detected in EST sequences [35] or through RNA-Seq experiments [4]. The comparison of the reference sequence of the cultivated accession Heinz 1706 and the draft genome of *S. pimpinellifolium* accession LA 1589 allowed the discovery of more than 5.4 million polymorphisms [11]. In the present study, a genome wide analysis of eight tomato lines allowed the discovery of more than 4 million SNPs and almost 128,000 InDels heterogeneously distributed across the chromosomes and the lines, which could be utilized for subsequent genetic analysis and for tomato improvement.

### Data quality and conditions of SNP discovery

The whole genome sequences of the eight lines were mapped onto the Heinz 1706 reference sequence for polymorphism discovery. Only 3-5% of the reads could not be mapped in spite of the stringent criteria. This rate is much lower than the ratio of 20% of unmapped reads for *S. pimpinellifolium*[11] or 15% in rice [7]. The low rate of unmapped reads resulted from (i) the high quality of the reference genome sequence and of the sequences produced, (ii) the low percentage of repeated sequences in the tomato genome and (iii) the low polymorphism level in the lines studied. In contrast, a strong reduction of the genome coverage was observed for Levovil on chromosome 9 in the region carrying the Tomato mosaic virus resistance gene (TM2-2), introgressed from a distant species, *S. peruvanium*[36]. The lower coverage observed only for this chromosome suggested that this phenomenon is caused by the high divergence between the species, and not by copy number variation or InDels with respect to the reference genome.

Illumina sequencing allowed the detection of more than 4 million SNPs. The error rate for Illumina sequencing is low (0.5 to 0.8 errors per 100 bp; [37]) and we applied a stringent selection criterion on read quality and retained only the SNPs that reached a minimum of 4x coverage per individual. When we increased the threshold to a minimum coverage of 8x, the number of SNPs dropped to about 3 million (75% remained), but several SNPs previously detected by Sanger sequencing [16] were no longer detected. We thus preferred a less stringent threshold. Finally the cross validation with the SNP array data gives a high level of confidence in the SNPs.

### Polymorphism detection is now possible in closely related accessions

Most of the SNPs were detected in one of the cherry tomato lines. Cherry tomato genome was shown to consist in an admixture between the genomes of *S. lycopersicum* and *S pimpinellifolium*[16], resulting in regions with high polymorphism compared to the reference genome (corresponding to introgressions) and regions with low polymorphism. The percentage of unique SNPs provided by the four *S. lycopersicum* were on average lower than 10% with the exception of chromosome 12, for which Stupicke provided 65% of the unique SNPs. This is in agreement with the distances among the lines (Additional file 8).

We assessed the number of common polymorphisms between lines in a pairwise approach including the SNPs detected in *S. pimpinellifolium* LA 1589 (Table 3). When comparing the two lines most distant from the reference genome, Cervil and Plovdiv (carrying 2.02 million and 1.45 million SNPs, respectively), 828,000 SNPs were common to both lines, and thus 1.19 and 0.62 million SNPs were specific to each line. If we compare these two lines to the *S. pimpinellifolium* genome, we detected 1.53 and 1.06 million SNPs common to the wild species, respectively. Thus each line carried around 500,000 SNP not detected when comparing LA 1589 and Heinz 1706. This suggested that there is still a high number of SNPs to be discovered in *S. pimpinellifolium* and cherry-type accessions.

**Table 3:** **Number of common SNP (upper diagonal) and InDel (lower diagonal) in all the pairs of comparisons (SNP defined with a depth higher than 4 in both accessions, except for LA 1589,** ***S. pimpinellifollium*** **)**

		***S. lyc***	***S. lyc***	***S. lyc***	***S. lyc***	***S. l. cera***	***S. l. cera***	***S. l. cera***	***S. l. cera***	***S. pim***
SNP InDel	Nb vs Ref.	LA0147	Levovil	Ferum	Stupicke	Criollo	LA1420	Plovdiv	Cervil	LA 1589
Nb vs Ref.		182,371	271,458	306,083	356,655	1,042,928	1,358,257	1,457,098	2,028,568	4,524,892
LA 0147	7,969		82,460	85,695	116,904	63,915	79,616	76,642	87,389	76,628
Levovil	2,894	517		49,318	80,009	54,538	49,482	53,472	78,907	67,886
Ferum	4,532	715	353		71,995	122,094	116,987	68,448	64,689	207,309
Stupicke	10,886	1,544	540	738		70,024	217,565	244,284	111,531	193,353
Criollo	13,898	612	336	601	727		458,908	164,449	260,234	501,982
LA 1420	30,927	1,298	468	910	2,366	2,666		310,635	222,517	537,839
Plovdiv	33,966	1,227	460	722	2,621	1,262	3,106		828,296	1,065,584
Cervil	53,522	1,521	534	807	1,746	1,811	2,532	8,441		1,538,643
LA 1589	201,502	304	771	328	591	910	1,519	3,273	5,707	

Accessions consist in four *S. lycopersicum* (S. lyc), four cherry-type (*S. l. cera*) and one *S. pimpinellifolium* (*S. pim*) accessions. The first line and column indicate the number of SNP and InDel detected when compared to the reference genome [11].

In cultivated tomato, the scarcity of polymorphisms at the molecular level hampered the construction of saturated intraspecific maps until SNP discovery. Interestingly, even in the two lines that are the closest to the reference genome (LA 0147 and Levovil), one half to two-thirds of the SNPs remained specific to each line. Even the chromosomes with the lowest SNP number exhibited more than 3,000 SNPs. It is thus now possible to build genetic maps of almost any cross and address genetic questions at the intraspecific level, which was not possible before the availability of resequencing approaches.

New rapid and low-cost techniques based on next-generation sequencing platforms have been proposed to identify SNPs among lines. They consist either in a first genome reduction before sequencing or in low coverage whole genome resequencing such as Genotyping by Sequencing (GBS) [38]. In tomato, depending on the distance between the lines, genome reduction may lead to a low number of SNPs and GBS may be preferred in intraspecific crosses.

### Non random distribution of polymorphisms

**Figure 7:**
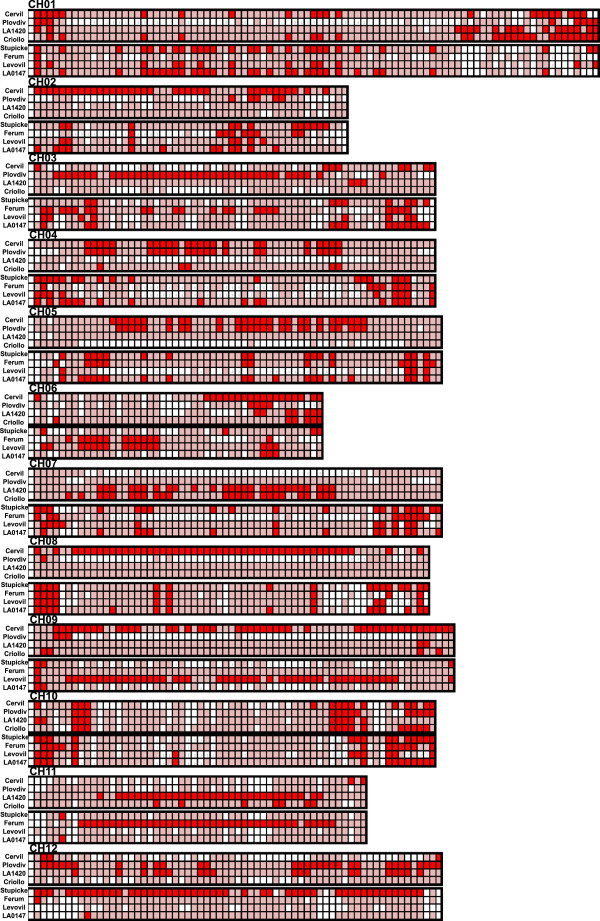
**Single nucleotide polymorphisms (SNP) variation across the genome in the two groups of four cherry-type tomato lines (Cervil, Plovdiv, LA 1420, Criollo from top to bottom) followed by the four cultivated lines (Stupicke, Ferum, Levovil and LA 0147 from top to bottom).** The *x*-axis represents the physical distance along the chromosomes, in which each tick-mark is one megabase. For each chromosome, the regions with extremely low SNP frequencies (less than 20% of the SNP from the group of four lines) are shown in white, and the regions with the 20% highest density of the SNPs (per group of four lines) are shown as red blocks.

The SNPs and InDels appeared non-randomly distributed between different chromosomes, but also within each chromosome (Figure 7). For instance, the overall number of SNPs detected on chromosome 10 was 10-fold lower than that on chromosome 5. Despite good coverage, a few regions appeared with a low SNP density in every line, for example: a few Mb in the middle of chromosomes 6 and 10 (although these regions were well covered). Such SNP “deserts” are also reported in other species [7] and must be confirmed in a larger sample. The SNP numbers were not related to the length of chromosomes or to gene density. Some regions, particularly at the distal ends of the chromosomes, carried a large proportion of the polymorphisms (Figure 7 and Additional file 2). The four *S. lycopersicum* lines also showed some regions poor in SNPs compared to the four cherry-type tomato lines, notably on chromosome 1, 5, 7 and 8. The most striking feature is the occurrence of large regions covering more than 10 Mb, present in one or two lines, and carrying large number of SNPs. This kind of pattern appeared on chromosome 2 and 8 for Cervil, on chromosome 3 for Plovdiv, on chromosome 9 for Levovil, on chromosome 11 for LA 1420 and Ferum and on chromosome 12 for Stupicke. Cervil and Plovdiv presented the same profiles for chromosome 4 and 5, with regions of low SNP density spread over regions of higher SNP density. Charles Rick, a pioneer in tomato genetics, underlined the role of natural hybridization in tomato, particularly in South America where cultivated accessions may grow close to wild relatives [39]: this phenomenon could have resulted in large introgressions, as shown here, particularly in the cherry tomato accessions.

Since the early 20th Century, tomato breeders have crossed cultivars with wild species in order to transfer resistance genes [17]. This has resulted first in the introgression of large DNA fragments of the wild species surrounding the resistance gene, inducing linkage drag. Subsequent backcrosses reduced the introgression size with more or less success [36]. The introgression of disease resistance genes in many cultivars has strongly influenced the SNP patterns. The reference genome of Heinz 1706 carries several fragments introgressed from *S. pimpinellifolium*[11], notably the resistance genes against *Verticilllium* (*Ve* gene on the top of chromosome 9) and *Fusarium* (*I2* gene on the bottom of chromosome 11). Other introgression events from *S. pimpinellifolium* in the Heinz 1706 genome have been reported, particularly a large one on chromosome 4 [11]. Among the resequenced lines, Ferum carried the *Ve* gene, Ferum and Criollo carried the *I2* resistance gene, but it was not possible to relate the presence/absence of these genes with variations in polymorphism rate. Cervil carried the resistance gene to *Fusarium radicis* on chromosome 9 (position not yet identified). Chromosome 9 of Levovil carried the TMV resistance gene introgressed from *S. peruvianum* (Tm2-2 gene, position 13,622,689). This introgression from a distant species reduced the coverage depth in the region, but the number of SNPs detected with the mapped reads was higher than in the rest of the genome for this line. For the other regions it is more difficult to identify any known introgressed gene. These regions often cover the centromeric regions where the recombination rate is lower [33] and thus an introgressed fragment may cover a large part of the chromosome. Our results confirmed the observations based on the SNP array showing that variable polymorphism rates from one chromosome to another reveal the breeding history [32].

### Structural modification

In *S. pimpinellifolium*, 3,423 genome regions were lacking when compared to Heinz 1706 with large regions missing on chromosome 1 and 10 [11]. We detected around 1,700 CNV in the five lines with a coverage depth higher than 10x. This number is much lower than in allogamous species like maize where structural variations are much more frequent [10]. The frequency of CNV could be related to the SNP frequency, except for LA 0147 which presented an excess of InDels and CNV compared to its SNP number.

### SNP annotation

Annotation of SNPs and InDels in the eight lines showed that less than 5% of the polymorphisms occurred in coding regions. The 55,337 unique polymorphisms with significant effects (non synonymous, splice site, start or stop site variation) affected 20,959 genes. Non synonymous to synonymous ratios ranged from 1.34 on average in the four cherry tomato lines to 1.48 on average in the four cultivated lines. These values are close to those detected in soybean (1.36 and 1.38 in wild and cultivated accessions, respectively [8]), and in rice (1.2; [7]). The nucleotide diversity decreased in the coding sequences in every line, as expected. The four cultivated lines exhibited a lower overall diversity compared to the four cherry-type accessions, but also a lower ratio of SNP between non coding and coding sequences, reflecting the purifying effect of breeding selection. SNPs with large effects are often detected at higher frequencies in stress related genes as shown in maize [9] or in *Arabidopsis thaliana*[5]. An excess of genes related to cell death and regulator genes was also detected in the polymorphisms with high effects detected between *S. lycopersicum* and *S. pimpinellifolium*[11]. In the present study, we observed the same trend for the 2,012 and 887 genes showing high effect SNPs and InDels, as well as in the 1,235 genes affected by CNVs.

### A catalogue of variations useful for genetic studies

For years, we have studied the progeny of the cross between two of the studied lines, Cervil and Levovil. We identified several QTLs for fruit quality traits [40] and fine mapped some of them [41]. The availability of the reference genome allowed us to rapidly positionally clone a QTL controlling locule number [42]. The availability of the annotated sequences of both lines considerably facilitates the identification of the genes and alleles underlying the QTLs. Recently we constructed a Multi Allelic Genetic Intercross (MAGIC) population derived from the intercross of the eight lines. With a broad genetic basis and higher recombination fraction than bi-parental populations, the MAGIC population is particularly interesting for QTL identification [43]. Based on our resequencing effort, a set of SNPs regularly spaced along the chromosomes was identified in order to construct a genetic map of the population and for QTL mapping. Genome wide association is a complementary approach to identify QTLs. The admixture state of cherry tomato accessions is particularly adapted to such analysis [16, 44]. Once a region carrying a QTL is identified using an SNP array, the availability of the catalogue of SNPs present in that region and their annotation will be very useful for the identification of the putative SNP responsible for the QTL. Beyond providing a highly valuable resource in terms of polymorphism, this catalogue allows a look at the past, revisiting and interpreting the breeding history of accessions and foreseeing the future through the use of high density mapping and detection of fine haplotypes and imputation of SNPs on large accessions panels.

## Conclusion

Next generation sequencing has provoked a revolution in plant research and genetics and offers a wide range of applications [45]. In the present study, we used eight very diverse lines to detect more than 4 million SNPs, around 128,000 InDels and 1,700 CNVs. We showed that it was possible to detect thousands of SNPs even in closely related lines like Heinz 1706 and Levovil, offering new perspectives for tomato breeding. The distribution of SNPs was heterogeneous and revealed traces of ancient introgressions or breeding efforts. These data are particularly useful for the identification of QTLs and new alleles. Today several projects resequencing tomato accessions are underway [46]. The number of SNPs available will thus rapidly increase, allowing the identification of new introgressions and regions of the genome under selection.

## Methods

### Materials and library construction and sequencing

DNA was extracted from young leaves of four *Solanum lycopersicum* lines (Levovil, Stupicke Polni Rane – herein Stupicke, LA 0147 and Ferum) with large fruits and four cherry-type accessions, *S. l. var cerasiforme* lines (Cervil, Criollo, Plovdiv24A –herein Plovdiv, and LA 1420). LA 0147 and LA 1420 were kindly provided by the Tomato Genetics Resource Center, Davis, California. Cervil and Levovil were provided by Vilmorin Seed Company. The other lines are conserved in the Genetic Resource Center in INRA, Avignon (France). Genomic DNA quality control, Illumina libraries construction and sequencing on GAIIx (Genome Analyser, Illumina corporation Inc.) were performed at Unité Etude du Polymorphisme des Génomes Végétaux, INRA, using the Bank service and Illumina sequencers facilities of CEA-Institut de Génomique/CNG, Evry (France). All the DNA samples went through quality control successfully. Non-indexed paired-ends (PE) libraries were carried out with an initial input DNA of 3 μg by following the Illumina Paired-End DNA Sample Prep protocol (Part # 1005063 Rev.D, February 2010) with some modifications: 3 μg of Genomic DNA were submitted to fragmentation by using Adaptive Focused Acoustics (AFA) process from Covaris technology (S2 Focused-Ultrasonicator). After end-repairing and adapters ligation, a 400-bp size selection of DNA fragments was performed by band excision after gel electrophoresis. The steps of fragmentation, ligation and PCR were validated on Agilent 2100 BioAnalyser. One lane per library was originally loaded on several flow cells, Clusters amplification was performed either on a Clustering Station or a Cbot, then sequencing was performed as a PE 76b/101b run length on GAIIx, following technological improvements. Data from a total of 14 sequencing runs were collected, 3 single 101 bp, (not paired-end because sequencing failed for the read-2), one 76-bp long and all others 101 bp-long. A first analysis was conducted by applying the process of quality control and cleaning for validation of the sequencing data.

### Sequence processing, mapping and SNP/InDel calling

Before the mapping step, sequences were cleaned and filtered with Python home-made scripts (available upon request to the authors). First, duplicated sequences were removed. Then low quality regions (phred score lower than 28) were cleaned, and sequences shorter than 30 nucleotides, or containing more than two N were removed. After the cleaning step, single and paired-end sequences were kept in different files. Cleaned reads were mapped onto the total Tomato reference genome (Sol Genomics Network, build 2.40; [11]) with the BWA algorithm (version 0.5.9; [47]) with mismatch penalty 3 and gap open penalty 5. The obtained BAM files were processed and adapted for the SNP calling program with SAMtools (version 1.1.18; [48]). Finally, SNP and InDel calling was performed using VarScan2 software (version 2.2.8; [49]) with a minimum depth of coverage of 4 per individual, a minimum quality of 30 per position and an allelic frequency of 0.9 for homozygous SNP/InDel and between 0.4 and 0.6 for heterozygous SNPs. In the last step, we removed the variants where the reference allele was an N or that were supported for more than 90% sequences in the same strand. The polymorphisms detected were also compared to the list of polymorphisms detected in the *S. pimpinellifolium* LA 1589 draft genome [11].

For the identification of copy number variation regions, the BAM files were analysed with the cn.Mops bioconductor package [50]. Only the five accessions with an average sequence depth greater than 10x were compared. Copy numbers were calculated and normalized for 2000 bp-windows. Calling of varying regions was done with the cn.Mops package default parameters.

### SNPs and InDels annotation

The VarScan2 output files (VCF) containing the homozygous SNPs and InDels were annotated based on their genomic location with the SnpEff software (version 2.1b; [34]). A tomato reference database, including the Tomato reference genome and the genome annotation (Sol Genomics Network, ITAG2.3), was created and used to categorize the effects of the allelic variants. Effects were classified by impact (High, Moderate, Low and Modifier) and effect (synonymous or non-synonymous amino acid replacement, start codon gain or loss, stop codon gain or loss or frame shifts). A GO term annotation file was created from the GFF file of genome annotation (Sol Genomics Network, ITAG2.3). Based on that file, a functional classification of the genes with allelic variants for each accession and impact category was performed. The enrichment in GO terms for each group was determined with a Fisher's Exact Test. All functional analyses were performed using the Blast2GO software [51].

### Validation of SNPs

To validate the identified homozygous SNPs, we compared the predicted genotypes and the genotypes obtained using the Infinium SolCAP’s Illumina Bead Chips [33] for six of the studied lines. Genomic DNA was extracted from young leaves of Cervil, Criollo, Ferum, LA 0147, Levovil, Stupicke and Heinz 1706. The samples were genotyped using SolCAP’s Illumina Bead Chips (Illumina, San Diego, California, USA) developed by the SolCAP project [31]. Genotyping was performed according to the manufacturer’s instructions for Illumina Infinium assay (Illumina Inc., San Diego, CA, USA). Intensity data was processed using the Illumina GenomeStudio v.2011.1 software.

### Data availability

This study is recorded in the European Nucleotide Archive (ENA) with the project number PRJEB4395 (http://www.ebi.ac.uk/ena/data/view/PRJEB4395). Raw sequences, i.e. 11 fastq files, have been deposited in ENA with accession numbers ERR327646 to ERR327656. Files containing the SNPs and INDELs identified for the eight accessions, i.e. 16 vcf files, have been deposited in ENA with accession numbers ERZ015686 to ERZ015701. BAM files and SNP characteristics are available upon request to the corresponding author and on the SolGenomics ftp site (ftp://ftp.solgenomics.net/projects/causse_tomato_snp8lines). Detailed information on CNV is available in Additional file 9 (CNV).

## Electronic supplementary material

Additional file 1: **Table listing the number of homozygous SNPs per chromosome and line.** (DOC 51 KB)

Additional file 2: **Figure showing the distribution of genes, homozygous and heterozygous SNPs and InDels along each chromosome over the 8 accessions and for each accession (using a window size of 100 kb).** (PDF 14 MB)

Additional file 3: **Table listing the number of homozygous InDels per chromosome and line.** (DOC 52 KB)

Additional file 4: **Table listing the number of heterozygous SNPs in genomic DNA of the eight accessions (0.4> allelic frequency > 0.6).** (DOC 52 KB)

Additional file 5: **Figure showing the distribution of the number of heterozygous SNPs.** (DOC 74 KB)

Additional file 6: **Table listing the classification of InDels in coding sequences according to their effects (snpEff version 2.1b).** (DOC 60 KB)

Additional file 7: **Table listing the number of regions showing a significant copy number variant (+: excess, -: default of copy number compared to the reference genome).** (DOC 54 KB)

Additional file 8: **Phylogenetic tree representing the 8 accessions, Heinz 1706 and LA 1589, constructed with the set of 7200 SNP positions common to the SolCap array.** (PDF 6 KB)

Additional file 9: **Table listing the position and characteristics of all the CNVs.** (ODS 56 KB)

## Abbreviations

SNP: Single nucleotide polymorphism
CNV: Copy number variation
InDel: Insertion deletion
SSR: Single sequence repeat
QTL: Quantitative trait locus.
