# Supplementary material for: Whole genome resequencing in tomato reveals variation associated with introgression and breeding events
Source: BMC Genomics. 2013 Nov 14;14(1):791. doi: 10.1186/1471-2164-14-791 (PMC4046683; doi:10.1186/1471-2164-14-791)
Supplement: Supplementary file 1 — Additional file 1: Table listing the number of homozygous SNPs per chromosome and line. (DOC 51 KB) [file 12864_2013_5531_MOESM1_ESM.doc]

| **Accession** | | Heinz 1706 Genome size (bp) | Cervil | Plovdiv | LA1420 | Criollo | Stupicke | Ferum | Levovil | LA0147 | Total unique |
| --- | --- | --- | --- | --- | --- | --- | --- | --- | --- | --- | --- |
|  | |  | *S.l.cera* | *S.l.cera* | *S.l.cera* | *S.l.cera* | *S. lyc* | *S. lyc* | *S. lyc* | *S. lyc* |  |
| **Chromosome / Totale** | 781 666 411 | | 2028568 | 1457098 | 1358257 | 1042928 | 356655 | 306083 | 271458 | 182371 | 4290679 |
| **ch00** | 21 805 821 | | 36505 | 15504 | 34967 | 29444 | 7156 | 10419 | 4956 | 4635 | 92 350 |
| **ch01** | 90 304 244 | | 66635 | 24541 | 39454 | 60542 | 33868 | 9623 | 27970 | 30101 | 140 192 |
| **ch02** | 49 918 294 | | 210477 | 45148 | 30960 | 50622 | 23219 | 8130 | 5768 | 7923 | 274 273 |
| **ch03** | 64 840 714 | | 62125 | 259566 | 46631 | 21216 | 5939 | 15069 | 5381 | 13026 | 357 900 |
| **ch04** | 64 064 312 | | 352954 | 313457 | 23427 | 200707 | 23631 | 19225 | 14586 | 15407 | 505 272 |
| **ch05** | 65 021 438 | | 440867 | 436825 | 189606 | 37615 | 11703 | 12751 | 3903 | 12423 | 616 803 |
| **ch06** | 46 041 636 | | 76610 | 12376 | 17516 | 14912 | 9158 | 10557 | 17073 | 11253 | 109 945 |
| **ch07** | 65 268 621 | | 7193 | 14614 | 280208 | 288626 | 8521 | 4166 | 5094 | 5889 | 385 516 |
| **ch08** | 63 032 657 | | 438657 | 32893 | 128650 | 120226 | 7215 | 5182 | 4636 | 6817 | **540 631** |
| **ch09** | 67 662 091 | | 238029 | 18049 | 46225 | 30310 | 12290 | 8615 | 142022 | 6339 | **411 088** |
| **ch10** | 64 834 305 | | 37593 | 40205 | 19735 | 22294 | 5331 | 4396 | 3630 | 5302 | 63 524 |
| **ch11** | 53 386 025 | | 33644 | 23213 | 320889 | 159749 | 17224 | 151279 | 14801 | 18463 | **499 546** |
| **ch12** | 65 486 253 | | 27279 | 220707 | 179989 | 6665 | 191400 | 46671 | 21638 | 44793 | 293 639 |

**Supplemental data S1 : Number of homozygous SNP in genomic DNA of the eight accessions.**

Accessions consist in four *S. lycopersicum* (S. lyc) and four cherry type (*S. l. cera*) accessions.
