# Supplementary material for: Whole genome resequencing in tomato reveals variation associated with introgression and breeding events
Source: BMC Genomics. 2013 Nov 14;14(1):791. doi: 10.1186/1471-2164-14-791 (PMC4046683; doi:10.1186/1471-2164-14-791)

Genes

SL2.40ch01

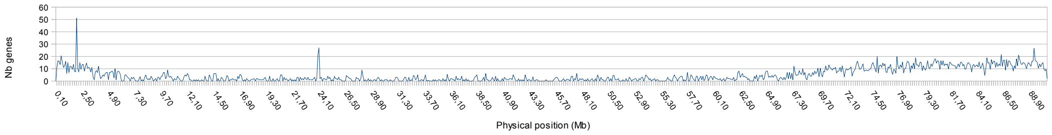

Homozygous SNP

SL2.40ch01

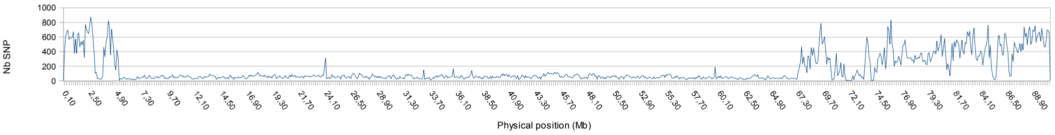

Heterozygous SNP

SL2.40ch01

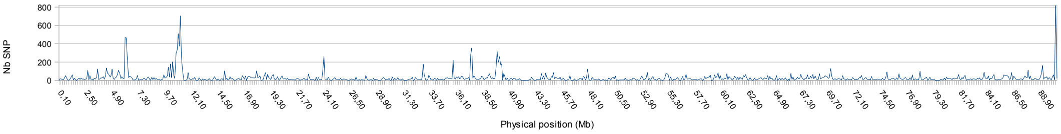

Homozygous InDel

SL2.40ch01

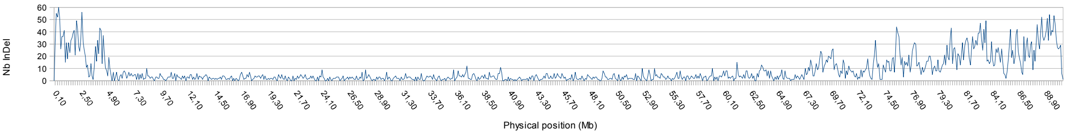

Cervil - Homozygous SNP

SL2.40ch01

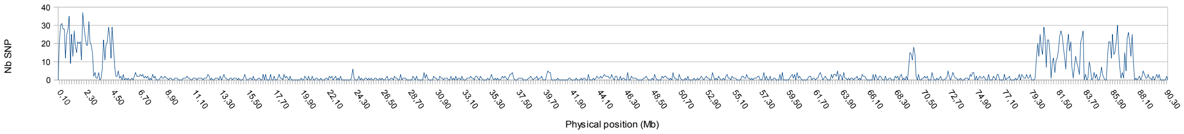

Criollo_new - Homozygous SNP

SL2.40ch01

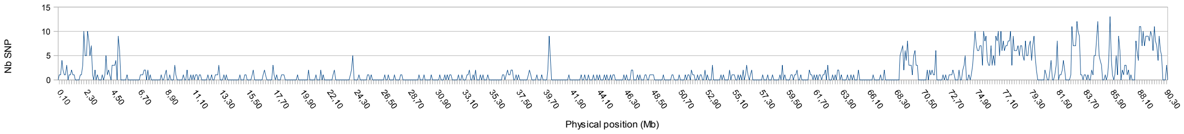

Ferum_new - Homozygous SNP

SL2.40ch01

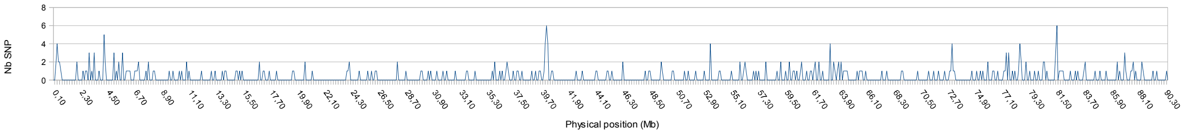

LA0147 - Homozygous SNP

SL2.40ch01

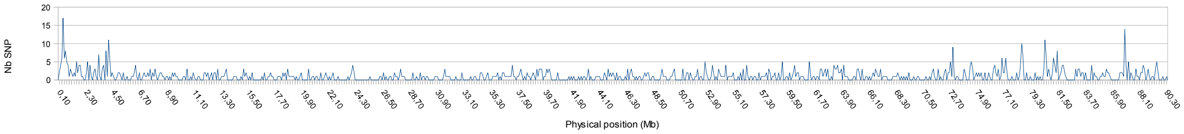

LA1420 - Homozygous SNP

SL2.40ch01

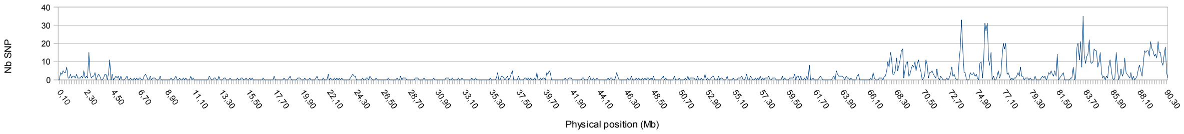

Levovil - Homozygous SNP

SL2.40ch01

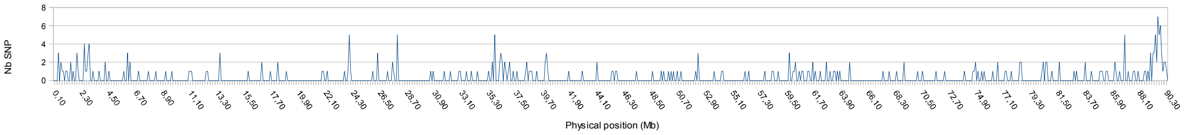

Plovdiv - Homozygous SNP

SL2.40ch01

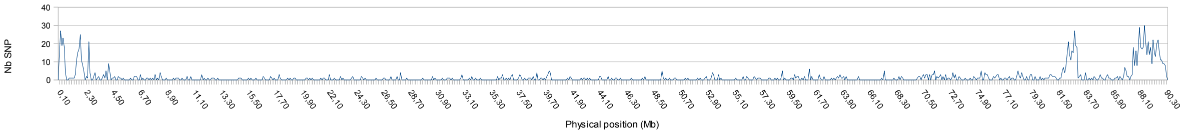

Stupicke - Homozygous SNP

SL2.40ch01

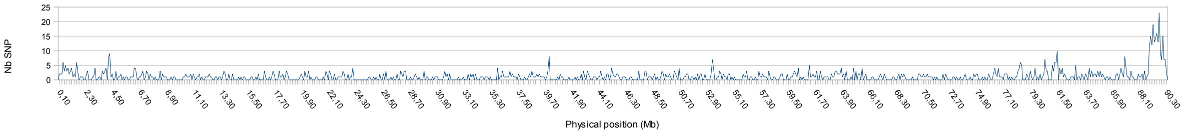

SNP hmz

Cervil - Homozygous SNP

SL2.40ch01

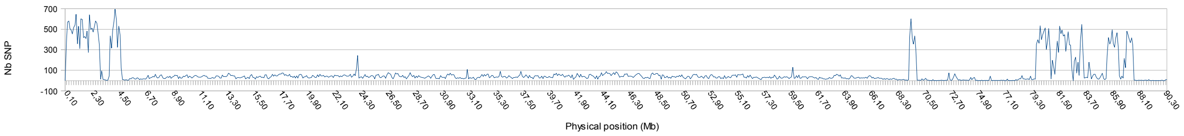

Criollo_new - Homozygous SNP

SL2.40ch01

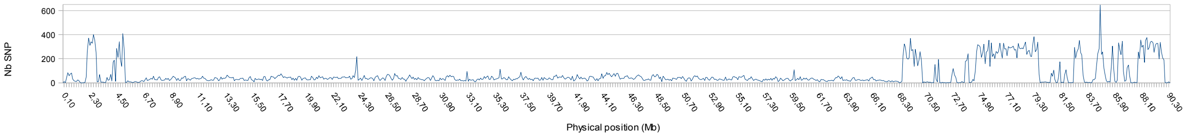

Ferum_new - Homozygous SNP

SL2.40ch01

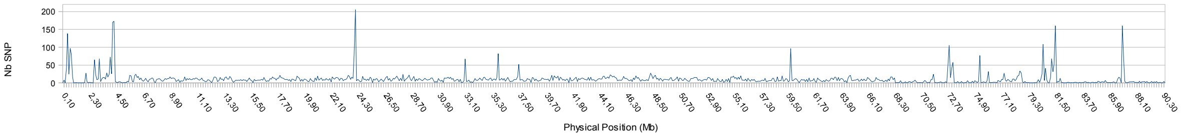

LA0147 - Homozygous SNP

SL2.40ch01

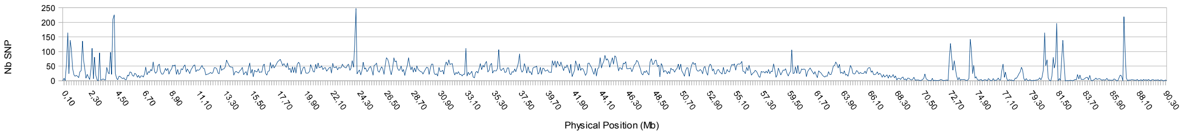

LA1420 - Homozygous SNP

SL2.40ch01

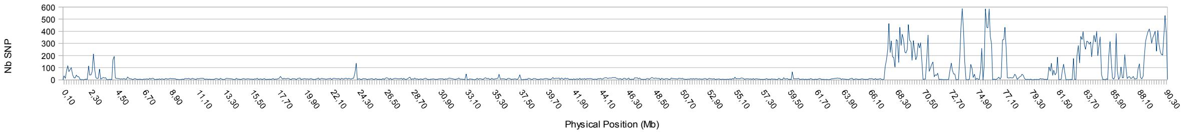

Levovil - Homozygous SNP

SL2.40ch01

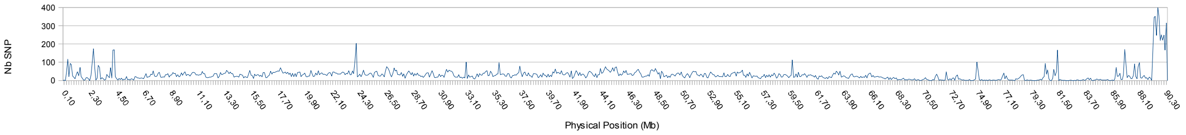

Plovdiv - Homozygous SNP

SL2.40ch01

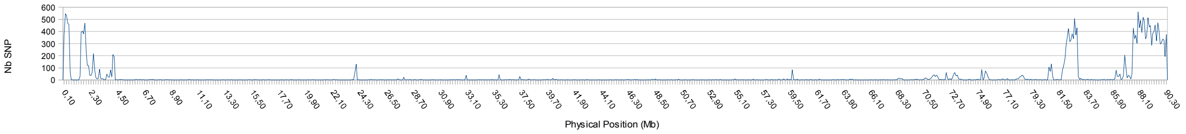

Stupicke - Homozygous SNP

SL2.40ch01

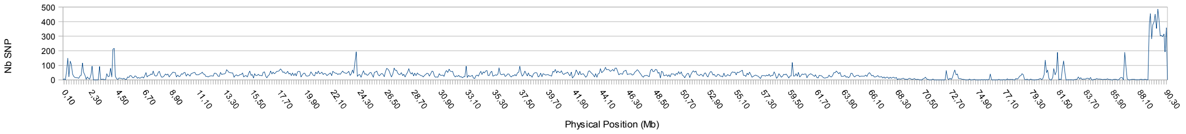

Cervil - Heterozygous SNP

SL2.40ch01

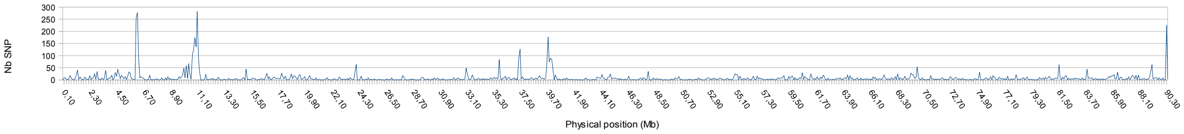

Criollo_new - Heterozygous SNP

SL2.40ch01

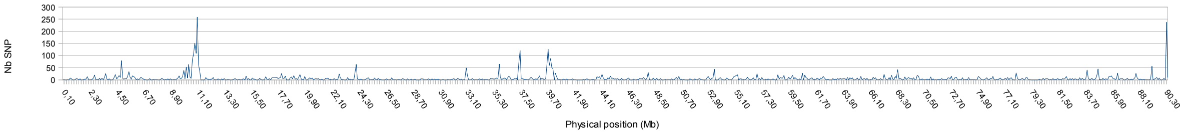

Ferum_new - Heterozygous SNP

SL2.40ch01

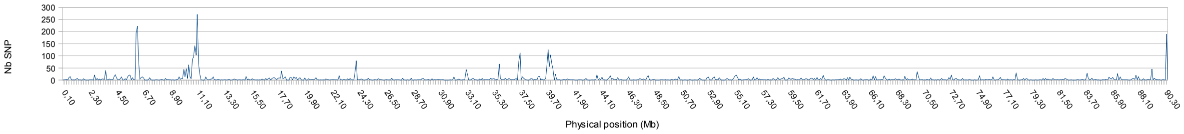

LA0147 - Heterozygous SNP

SL2.40ch01

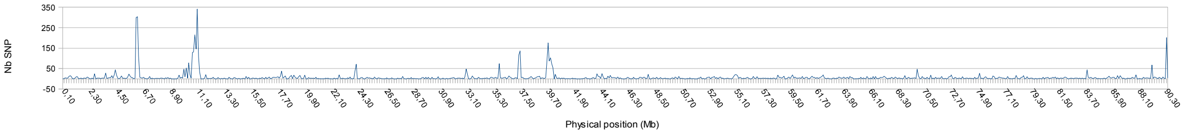

LA1420 - Heterozygous SNP

SL2.40ch01

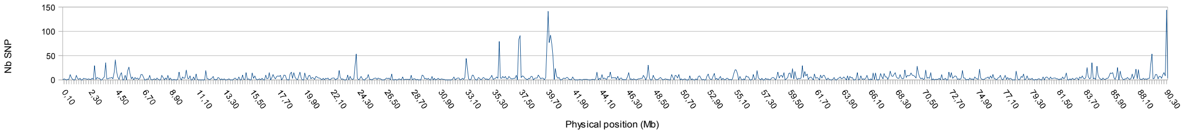

Levovil - Heterozygous SNP

SL2.40ch01

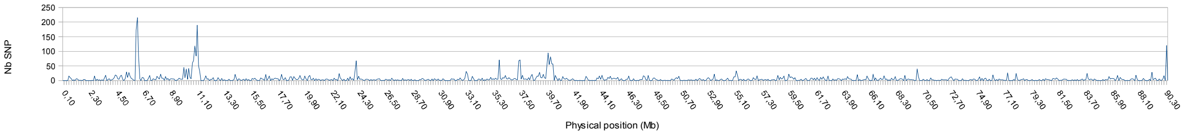

Plovdiv - Heterozygous SNP

SL2.40ch01

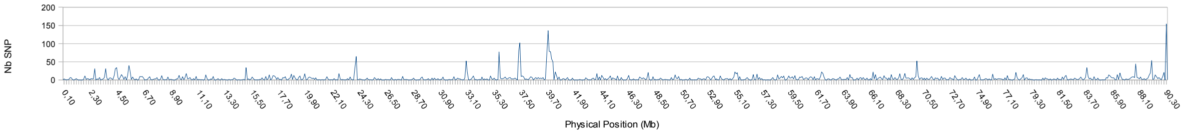

Stupicke - Heterozygous SNP

SL2.40ch01

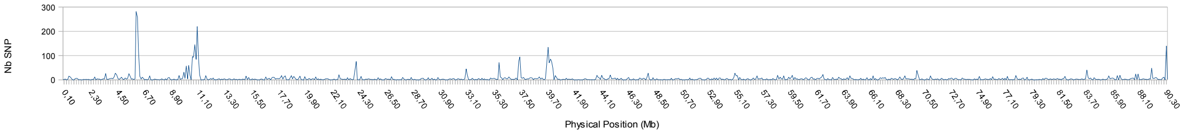

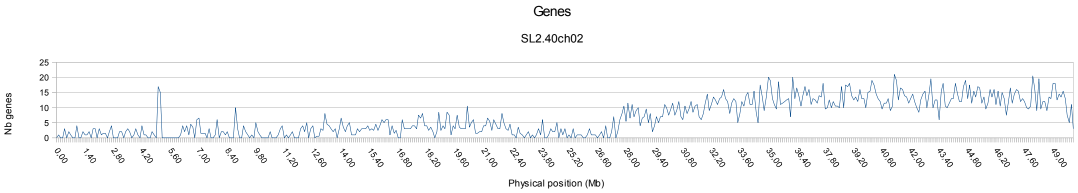
Genes
SL2.40ch02
Nb genes
0
5
10
15
20
25
0.00 1.40 2.80 4.20 5.60 7.00 8.40 9.80 11.20 12.60 14.00 15.40 16.80 18.20 19.60 21.00 22.40 23.80 25.20 26.60 28.00 29.40 30.80 32.20 33.60 35.00 36.40 37.80 39.20 40.60 42.00 43.40 44.80 46.20 47.60 49.00
Physical position (Mb)

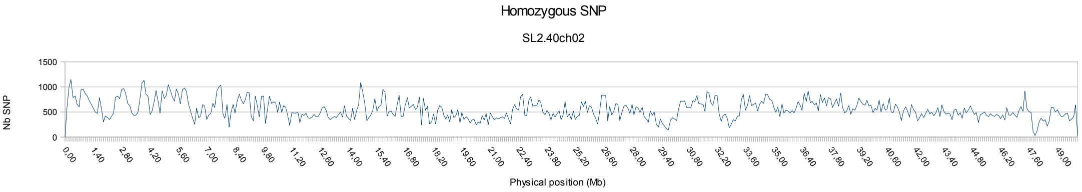
Homozygous SNP
SL2.40ch02
Nb SNP
0
500
1000
1500
0.00 1.40 2.80 4.20 5.60 7.00 8.40 9.80 11.20 12.60 14.00 15.40 16.80 18.20 19.60 21.00 22.40 23.80 25.20 26.60 28.00 29.40 30.80 32.20 33.60 35.00 36.40 37.80 39.20 40.60 42.00 43.40 44.80 46.20 47.60 49.00
Physical position (Mb)

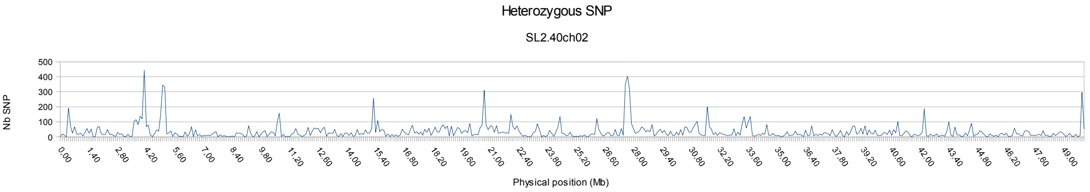
Heterozygous SNP
SL2.40ch02
Nb SNP
0
100
200
300
400
500
0.00 1.40 2.80 4.20 5.60 7.00 8.40 9.80 11.20 12.60 14.00 15.40 16.80 18.20 19.60 21.00 22.40 23.80 25.20 26.60 28.00 29.40 30.80 32.20 33.60 35.00 36.40 37.80 39.20 40.60 42.00 43.40 44.80 46.20 47.60 49.00
Physical position (Mb)

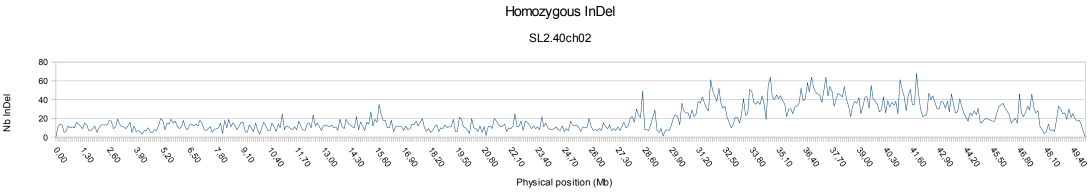
Homozygous InDel
SL2.40ch02
Nb InDel
0
20
40
60
80
0.00 1.30 2.60 3.90 5.20 6.50 7.80 9.10 10.40 11.70 13.00 14.30 15.60 16.90 18.20 19.50 20.80 22.10 23.40 24.70 26.00 27.30 28.60 29.90 31.20 32.50 33.80 35.10 36.40 37.70 39.00 40.30 41.60 42.90 44.20 45.50 46.80 48.10 49.40
Physical position (Mb)

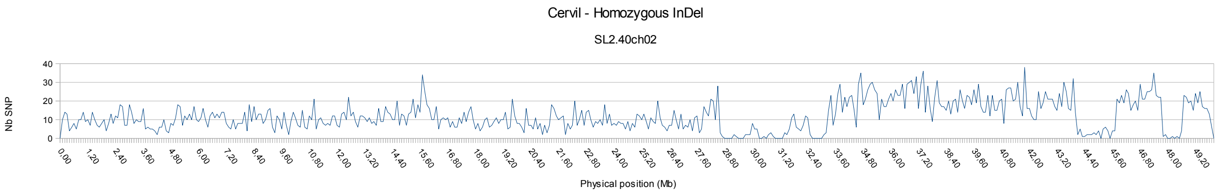
Cervil - Homozygous InDel
SL2.40ch02
Nb SNP
Physical position (Mb)

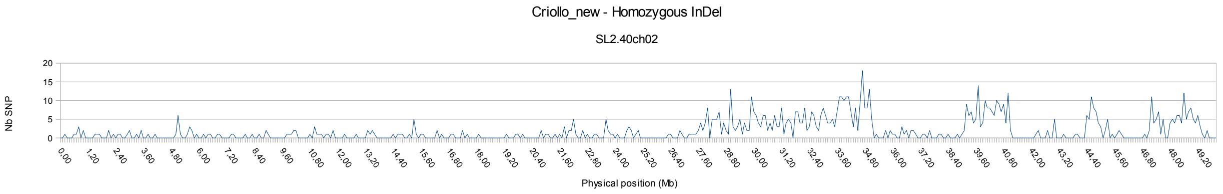
Criollo_new - Homozygous InDel
SL2.40ch02
Nb SNP
Physical position (Mb)

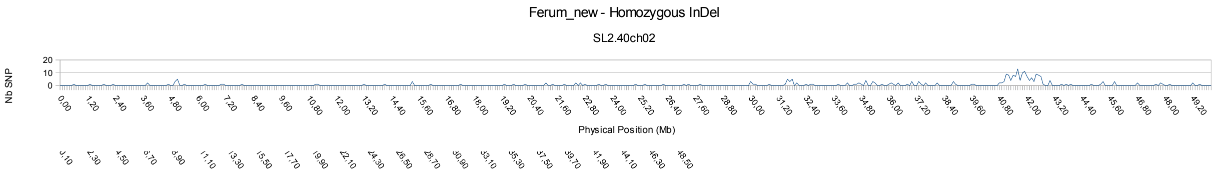
Ferum_new - Homozygous InDel
SL2.40ch02
Nb SNP
Physical Position (Mb)

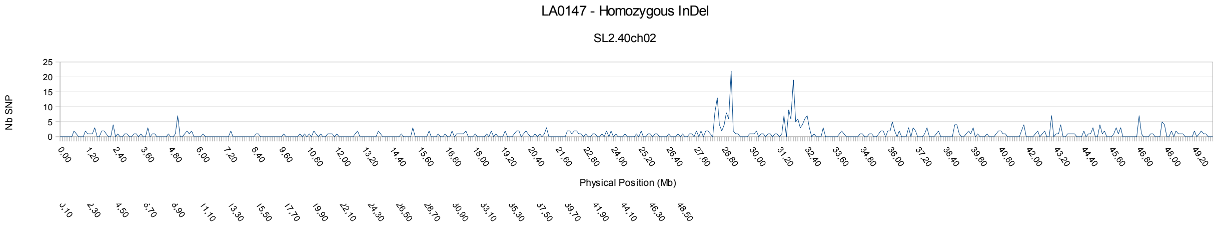
LA0147 - Homozygous InDel
SL2.40ch02
Nb SNP
Physical Position (Mb)

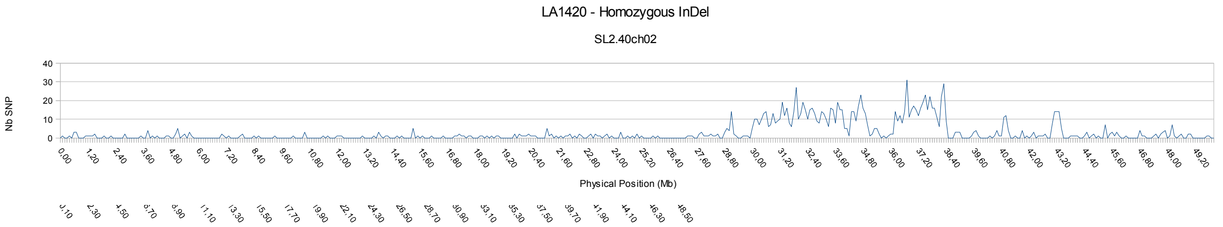
LA1420 - Homozygous InDel
SL2.40ch02
Nb SNP
Physical Position (Mb)

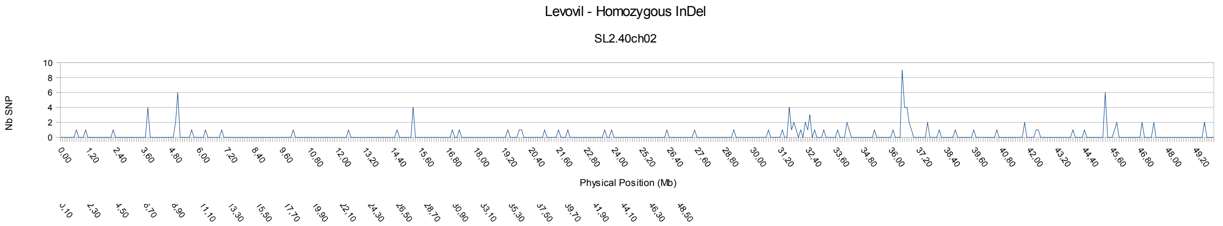
Levovil - Homozygous InDel
SL2.40ch02
Nb SNP
Physical Position (Mb)

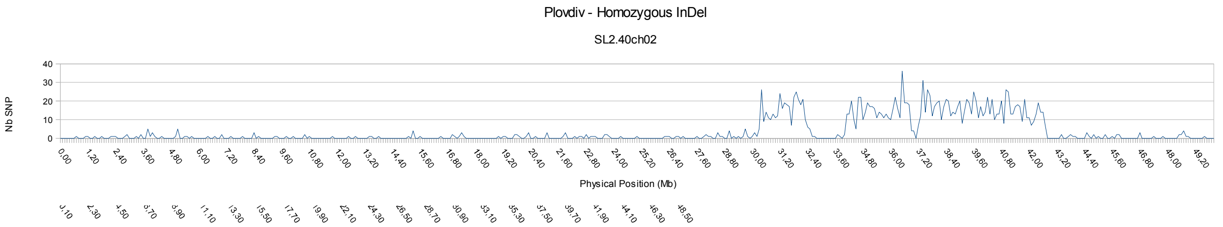
Plovdiv - Homozygous InDel
SL2.40ch02
Nb SNP
Physical Position (Mb)

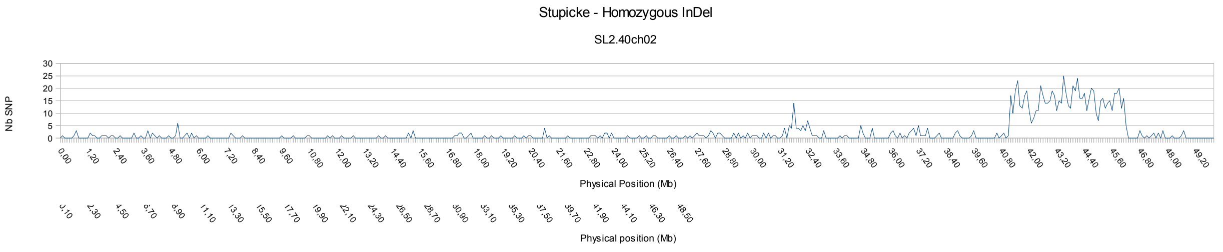
Stupicke - Homozygous InDel
SL2.40ch02
Nb SNP
Physical Position (Mb)
Physical position (Mb)

Cervil - Homozygous SNP

SL2.40ch02

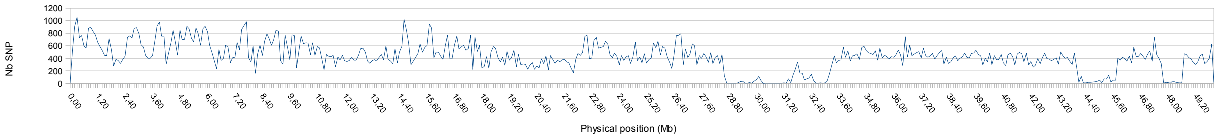

Criollo_new - Homozygous SNP

SL2.40ch02

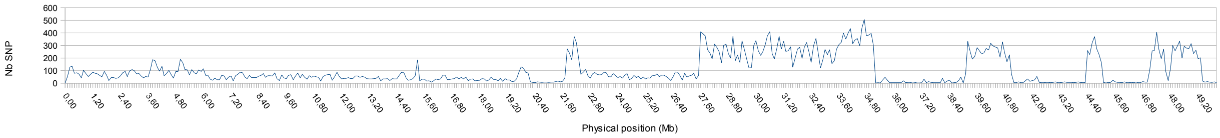

Ferum_new - Homozygous SNP

SL2.40ch02

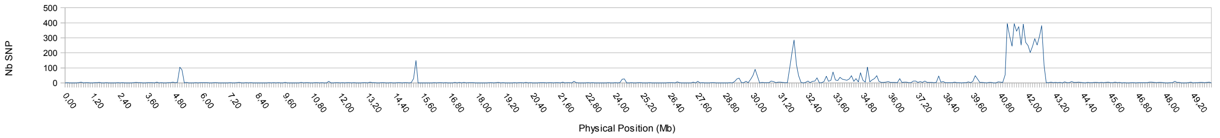

LA0147 - Homozygous SNP

SL2.40ch02

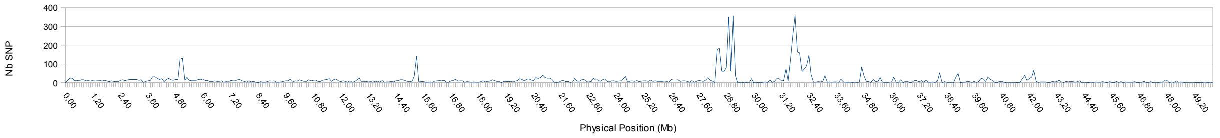

LA1420 - Homozygous SNP

SL2.40ch02

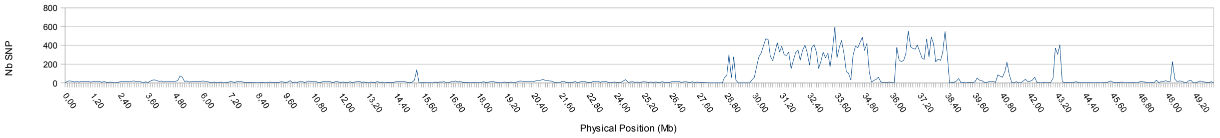

Levovil - Homozygous SNP

SL2.40ch02

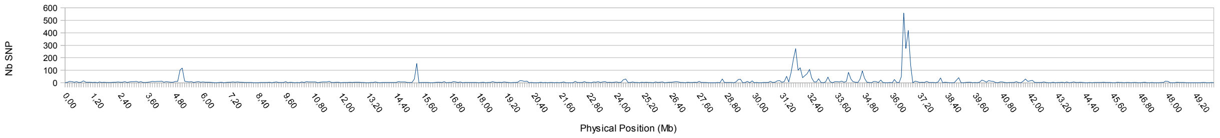

Plovdiv - Homozygous SNP

SL2.40ch02

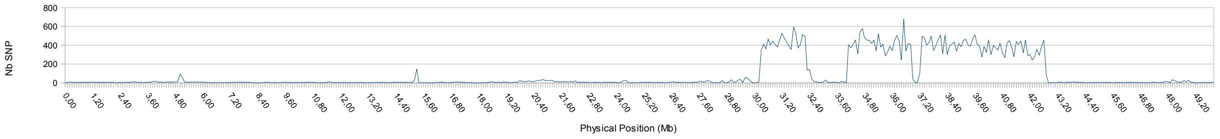

Stupicke - Homozygous SNP

SL2.40ch02

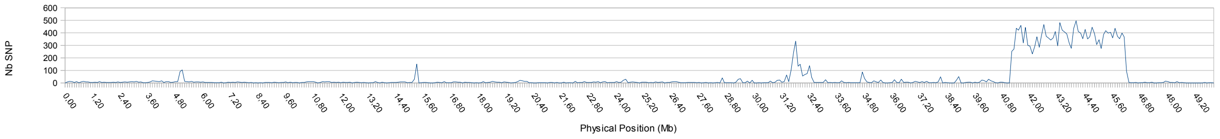

Cervil - Heterozygous SNP

SL2.40ch02

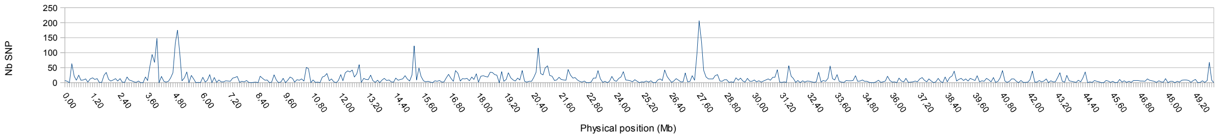

Criollo_new - Heterozygous SNP

SL2.40ch02

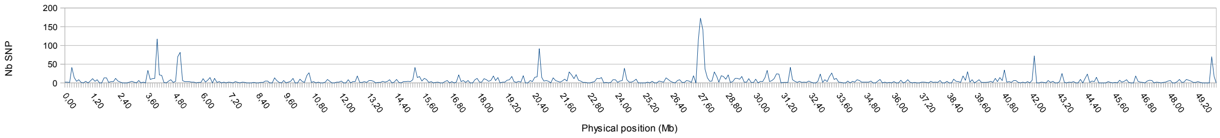

Ferum_new - Heterozygous SNP

SL2.40ch02

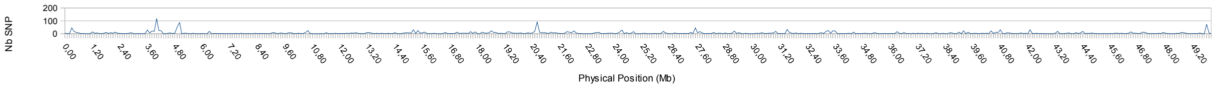

LA0147 - Heterozygous SNP

SL2.40ch02

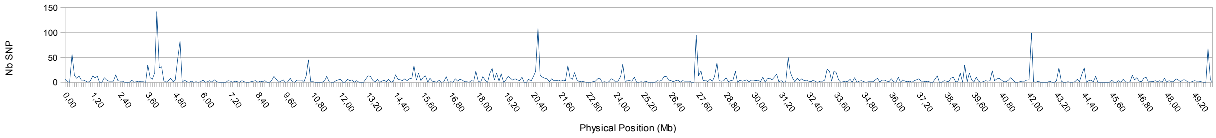

LA1420 - Heterozygous SNP

SL2.40ch02

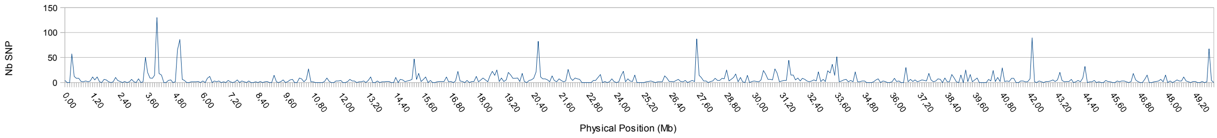

Levovil - Heterozygous SNP

SL2.40ch02

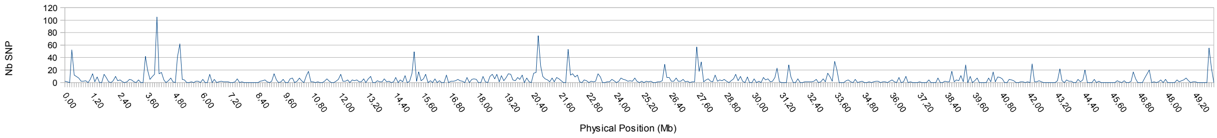

Plovdiv - Heterozygous SNP

SL2.40ch02

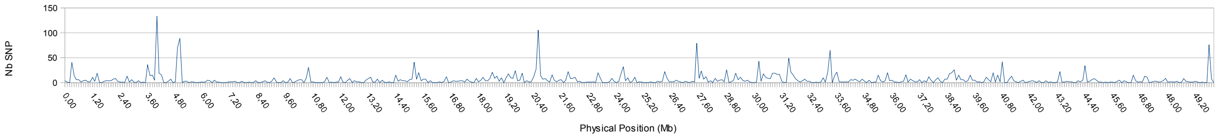

Stupicke - Heterozygous SNP

SL2.40ch02

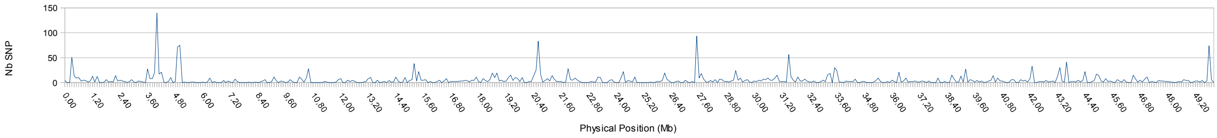

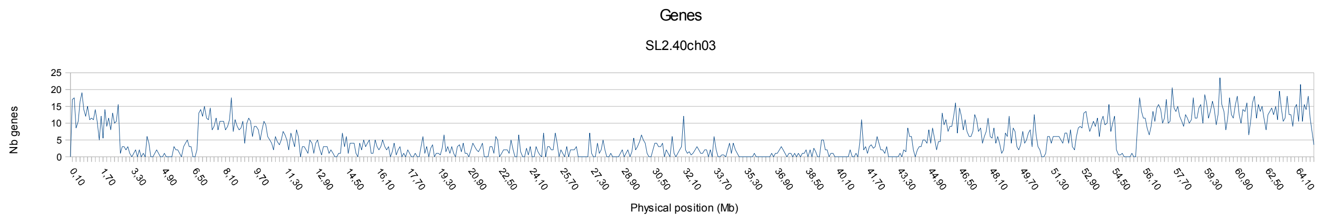
Genes
SL2.40ch03
Nb genes
Physical position (Mb)

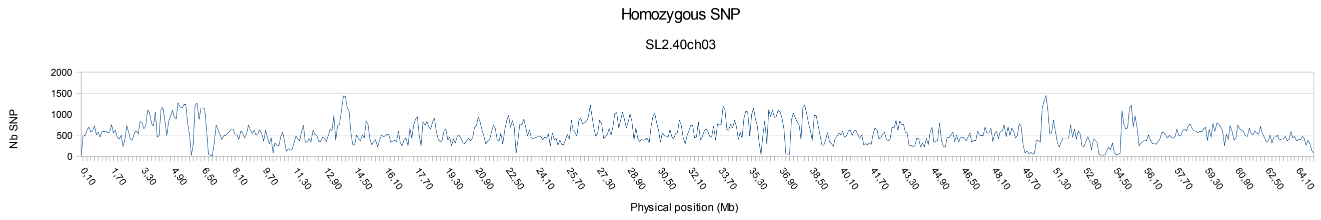
Homozygous SNP
SL2.40ch03
Nb SNP
Physical position (Mb)

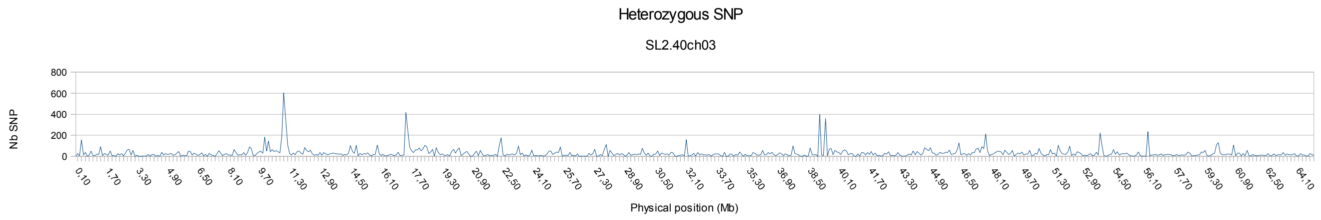
Heterozygous SNP
SL2.40ch03
Nb SNP
Physical position (Mb)

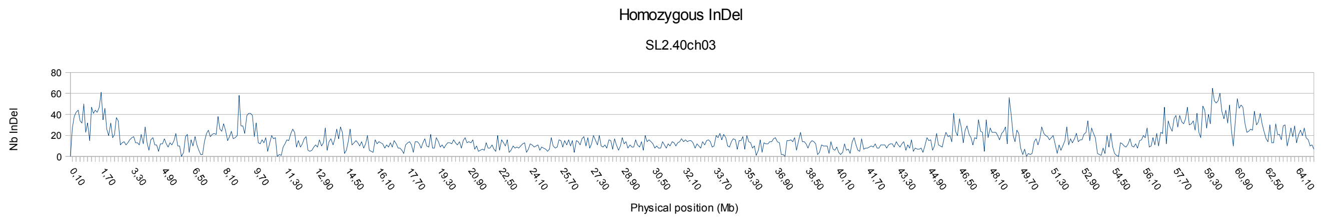
Homozygous InDel
SL2.40ch03
Nb InDel
Physical position (Mb)

Cervil - Homozygous InDel

SL2.40ch03

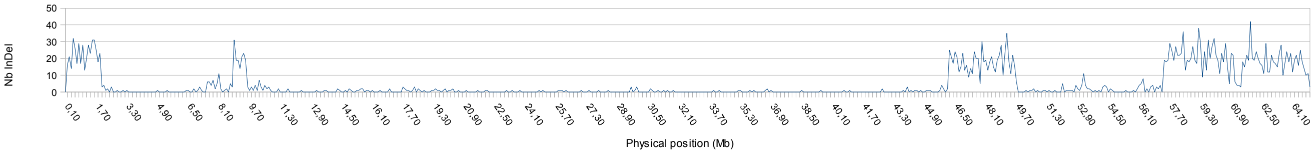

Criollo_new - Homozygous InDel

SL2.40ch03

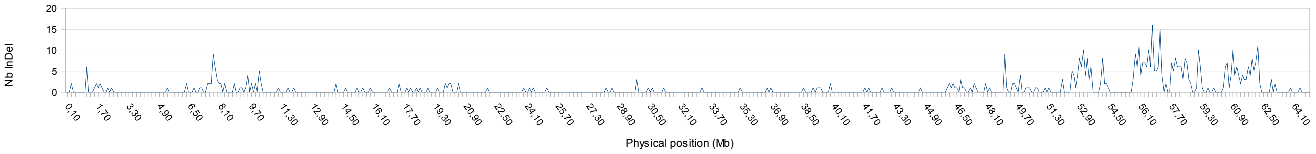

Ferum_new - Homozygous InDel

SL2.40ch03

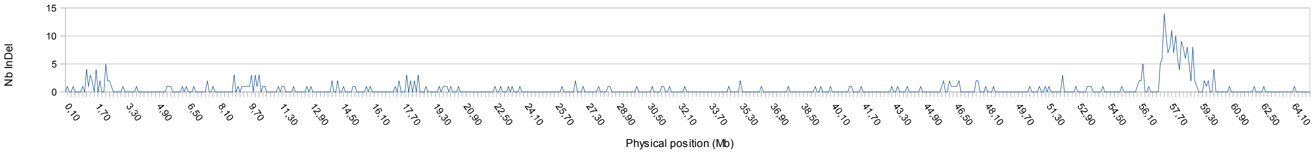

LA0147 - Homozygous InDel

SL2.40ch03

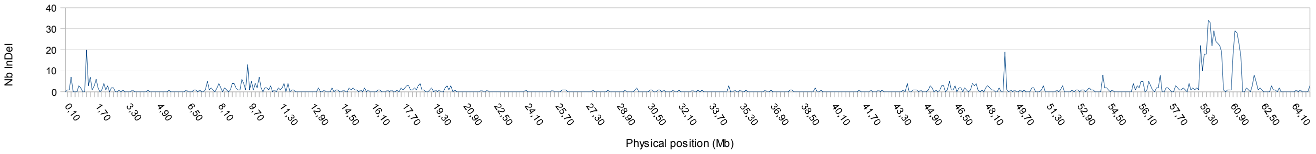

LA1420 - Homozygous InDel

SL2.40ch03

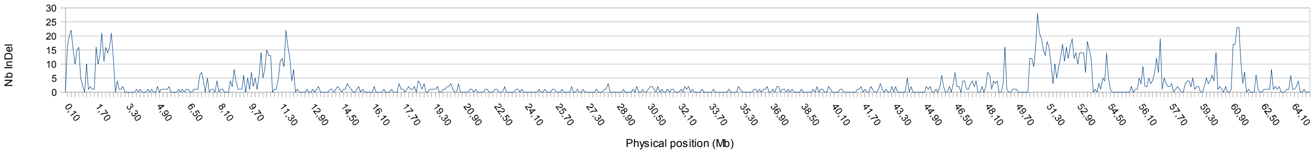

Levovil - Homozygous InDel

SL2.40ch03

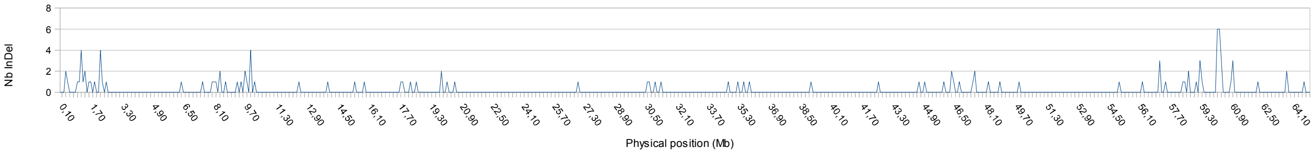

Plovdiv - Homozygous InDel

SL2.40ch03

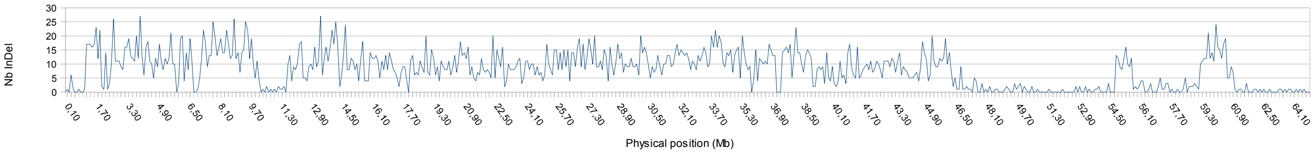

Stupicke - Homozygous InDel

SL2.40ch03

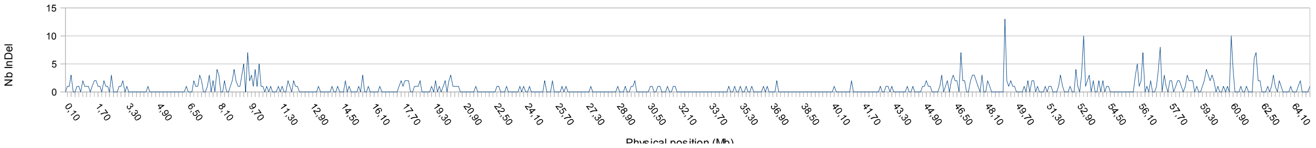

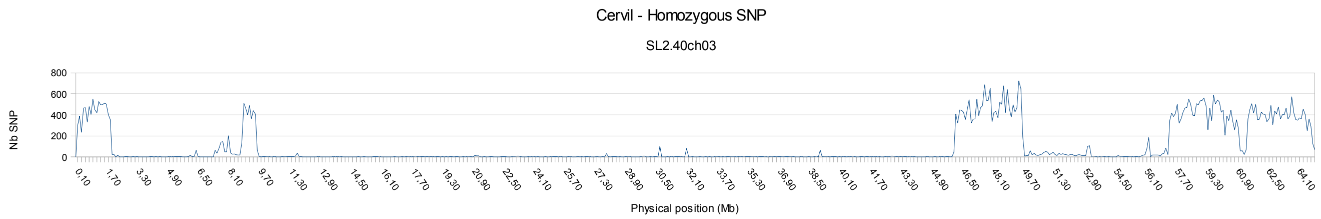
Cervil - Homozygous SNP
SL2.40ch03
Nb SNP
0 200 400 600 800
0.10 1.70 3.30 4.90 6.50 8.10 9.70 11.30 12.90 14.50 16.10 17.70 19.30 20.90 22.50 24.10 25.70 27.30 28.90 30.50 32.10 33.70 35.30 36.90 38.50 40.10 41.70 43.30 44.90 46.50 48.10 49.70 51.30 52.90 54.50 56.10 57.70 59.30 60.90 62.50 64.10
Physical position (Mb)

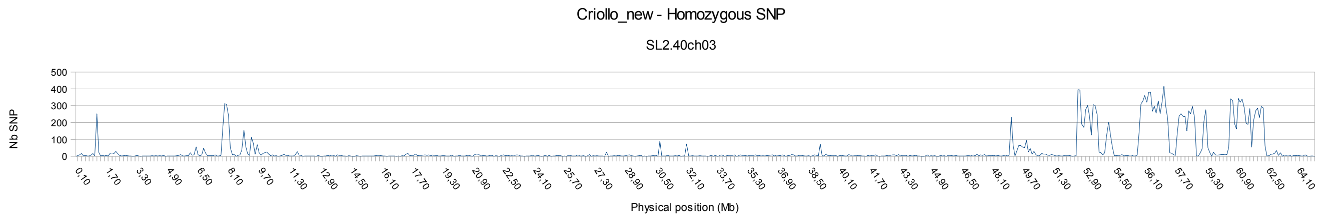
Criollo_new - Homozygous SNP
SL2.40ch03
Nb SNP
0 100 200 300 400 500
0.10 1.70 3.30 4.90 6.50 8.10 9.70 11.30 12.90 14.50 16.10 17.70 19.30 20.90 22.50 24.10 25.70 27.30 28.90 30.50 32.10 33.70 35.30 36.90 38.50 40.10 41.70 43.30 44.90 46.50 48.10 49.70 51.30 52.90 54.50 56.10 57.70 59.30 60.90 62.50 64.10
Physical position (Mb)

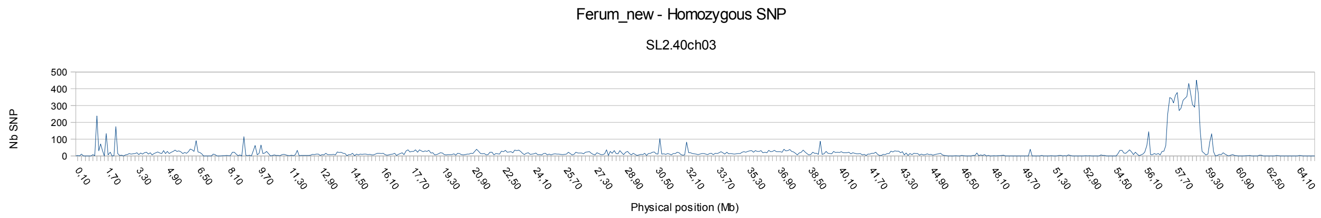
Ferum_new - Homozygous SNP
SL2.40ch03
Nb SNP
0 100 200 300 400 500
0.10 1.70 3.30 4.90 6.50 8.10 9.70 11.30 12.90 14.50 16.10 17.70 19.30 20.90 22.50 24.10 25.70 27.30 28.90 30.50 32.10 33.70 35.30 36.90 38.50 40.10 41.70 43.30 44.90 46.50 48.10 49.70 51.30 52.90 54.50 56.10 57.70 59.30 60.90 62.50 64.10
Physical position (Mb)

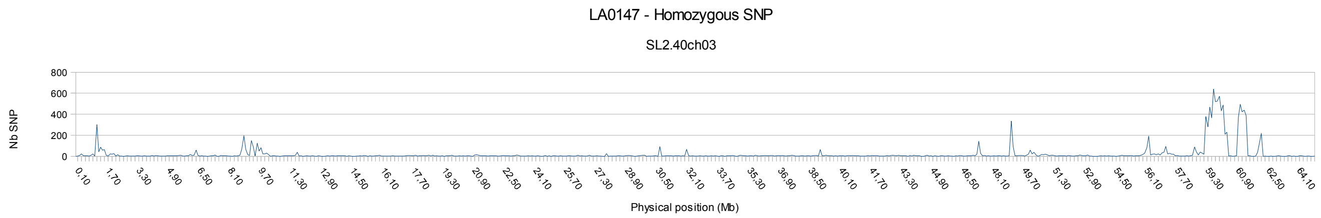
LA0147 - Homozygous SNP
SL2.40ch03
Nb SNP
0 200 400 600 800
0.10 1.70 3.30 4.90 6.50 8.10 9.70 11.30 12.90 14.50 16.10 17.70 19.30 20.90 22.50 24.10 25.70 27.30 28.90 30.50 32.10 33.70 35.30 36.90 38.50 40.10 41.70 43.30 44.90 46.50 48.10 49.70 51.30 52.90 54.50 56.10 57.70 59.30 60.90 62.50 64.10
Physical position (Mb)

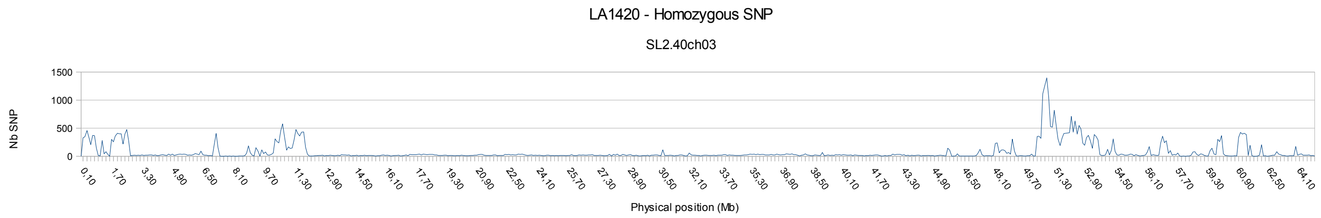
LA1420 - Homozygous SNP
SL2.40ch03
Nb SNP
0 500 1000 1500
0.10 1.70 3.30 4.90 6.50 8.10 9.70 11.30 12.90 14.50 16.10 17.70 19.30 20.90 22.50 24.10 25.70 27.30 28.90 30.50 32.10 33.70 35.30 36.90 38.50 40.10 41.70 43.30 44.90 46.50 48.10 49.70 51.30 52.90 54.50 56.10 57.70 59.30 60.90 62.50 64.10
Physical position (Mb)

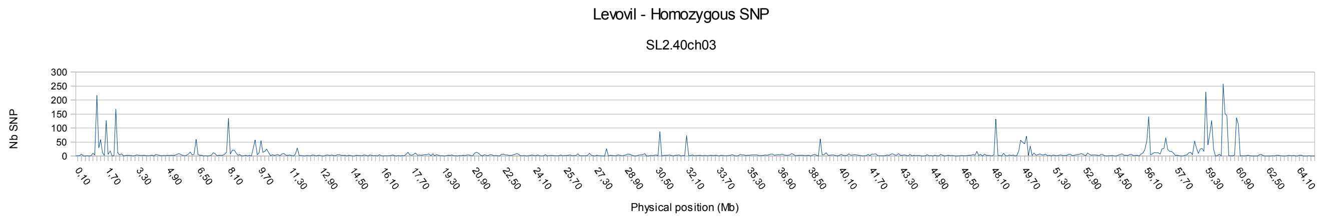
Levovil - Homozygous SNP
SL2.40ch03
Nb SNP
0 50 100 150 200 250 300
0.10 1.70 3.30 4.90 6.50 8.10 9.70 11.30 12.90 14.50 16.10 17.70 19.30 20.90 22.50 24.10 25.70 27.30 28.90 30.50 32.10 33.70 35.30 36.90 38.50 40.10 41.70 43.30 44.90 46.50 48.10 49.70 51.30 52.90 54.50 56.10 57.70 59.30 60.90 62.50 64.10
Physical position (Mb)

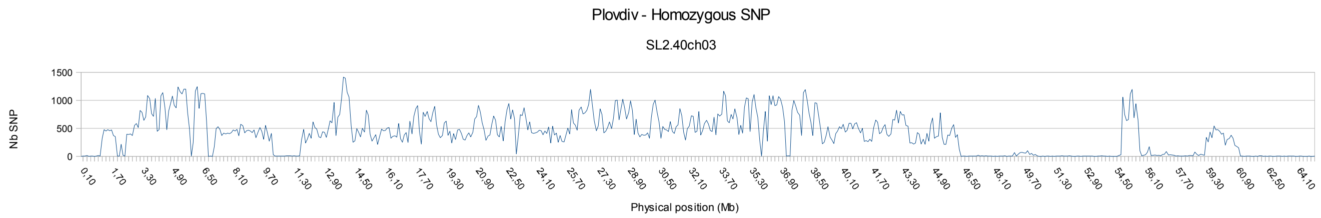
Plovdiv - Homozygous SNP
SL2.40ch03
Nb SNP
0 500 1000 1500
0.10 1.70 3.30 4.90 6.50 8.10 9.70 11.30 12.90 14.50 16.10 17.70 19.30 20.90 22.50 24.10 25.70 27.30 28.90 30.50 32.10 33.70 35.30 36.90 38.50 40.10 41.70 43.30 44.90 46.50 48.10 49.70 51.30 52.90 54.50 56.10 57.70 59.30 60.90 62.50 64.10
Physical position (Mb)

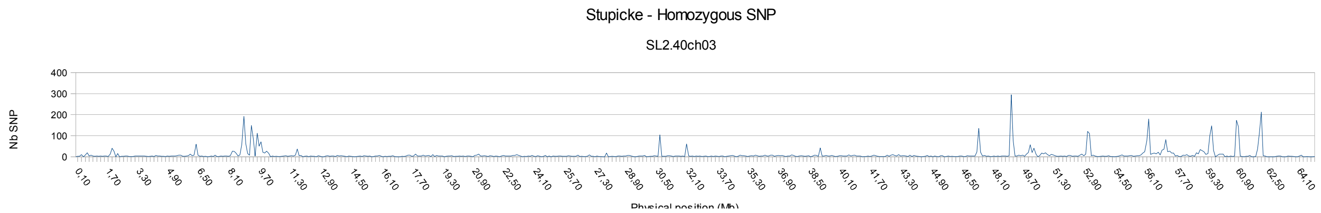
Stupicke - Homozygous SNP
SL2.40ch03
Nb SNP
0 100 200 300 400
0.10 1.70 3.30 4.90 6.50 8.10 9.70 11.30 12.90 14.50 16.10 17.70 19.30 20.90 22.50 24.10 25.70 27.30 28.90 30.50 32.10 33.70 35.30 36.90 38.50 40.10 41.70 43.30 44.90 46.50 48.10 49.70 51.30 52.90 54.50 56.10 57.70 59.30 60.90 62.50 64.10

Cervil - Heterozygous SNP

SL2.40ch03

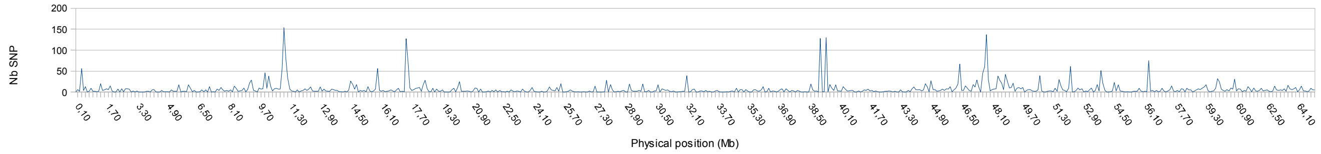

Criollo_new - Heterozygous SNP

SL2.40ch03

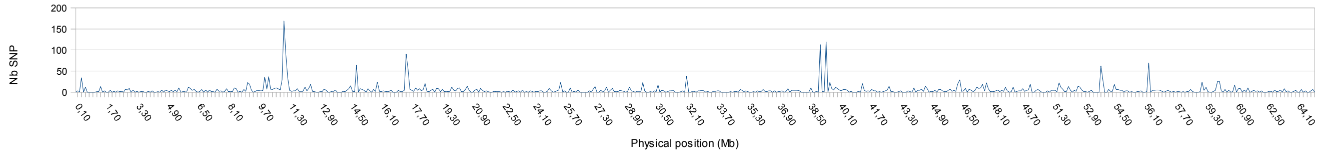

Ferum_new - Heterozygous SNP

SL2.40ch03

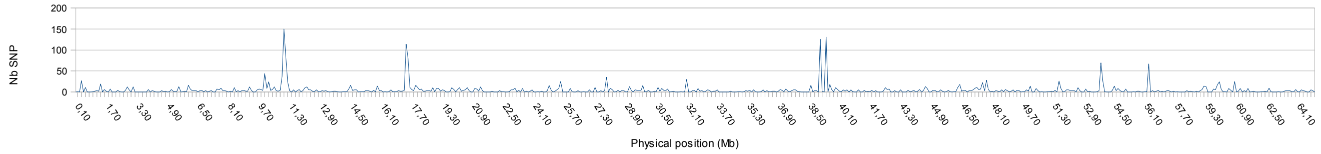

LA0147 - Heterozygous SNP

SL2.40ch03

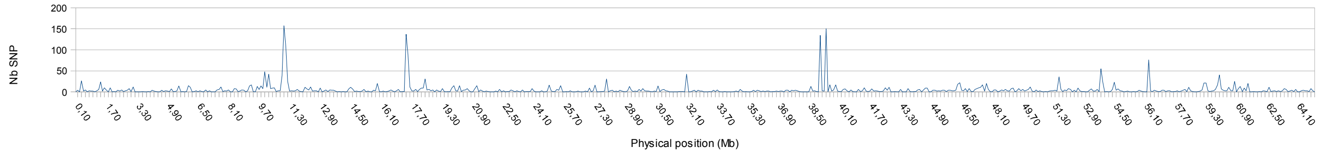

LA1420 - Heterozygous SNP

SL2.40ch03

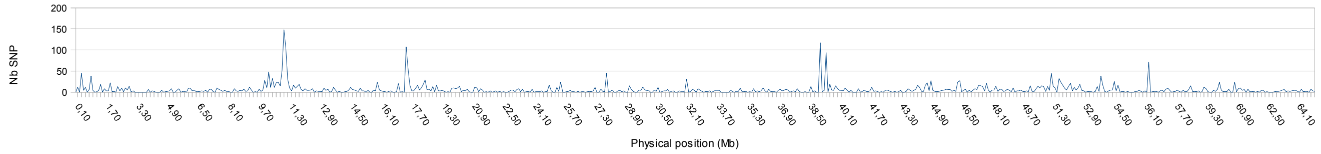

Levovil - Heterozygous SNP

SL2.40ch03

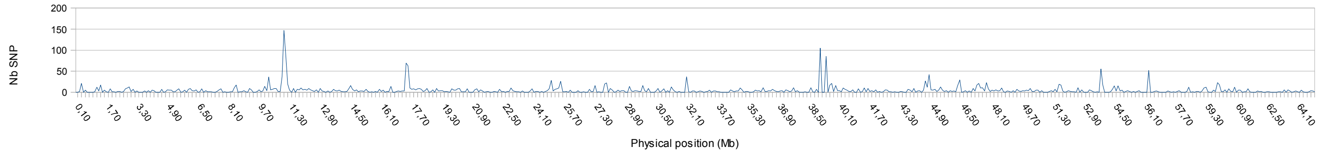

Plovdiv - Heterozygous SNP

SL2.40ch03

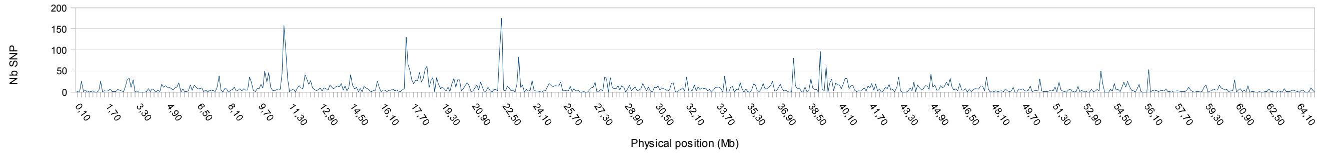

Stupicke - Heterozygous SNP

SL2.40ch03

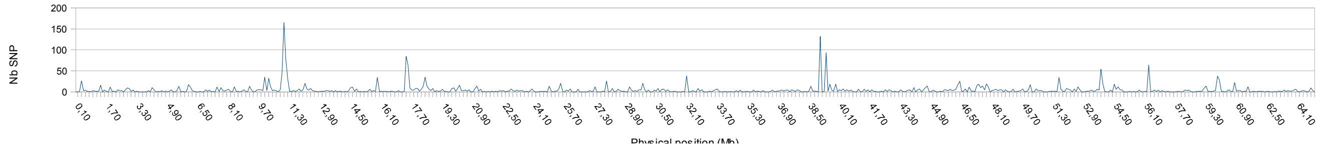

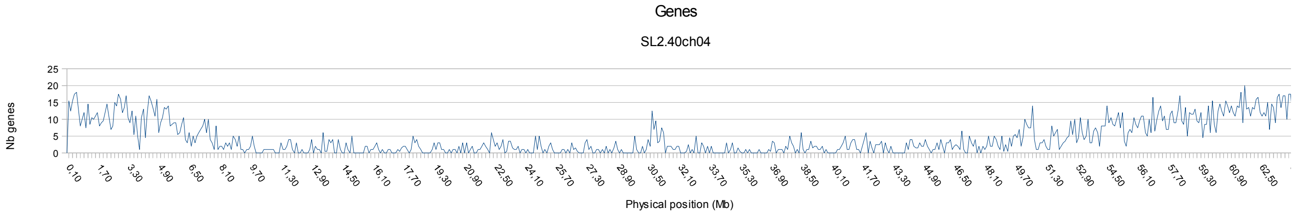
Genes
SL2.40ch04
Nb genes
Physical position (Mb)

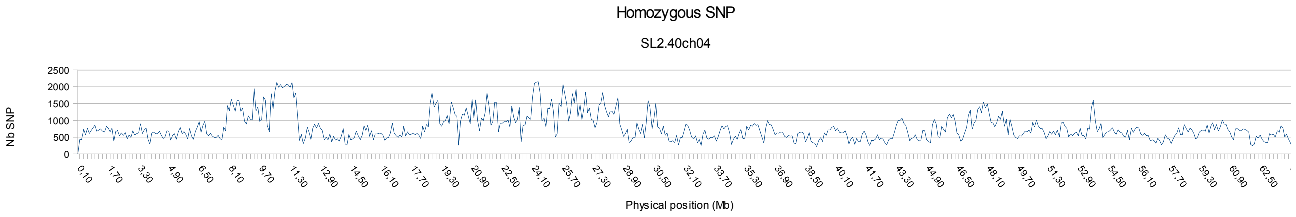
Homozygous SNP
SL2.40ch04
Nb SNP
Physical position (Mb)

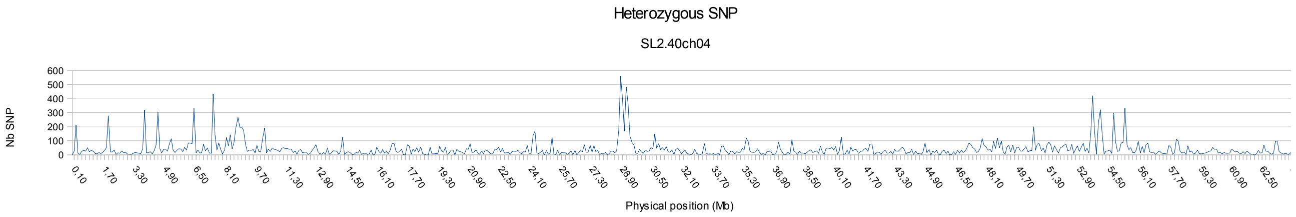
Heterozygous SNP
SL2.40ch04
Nb SNP
Physical position (Mb)

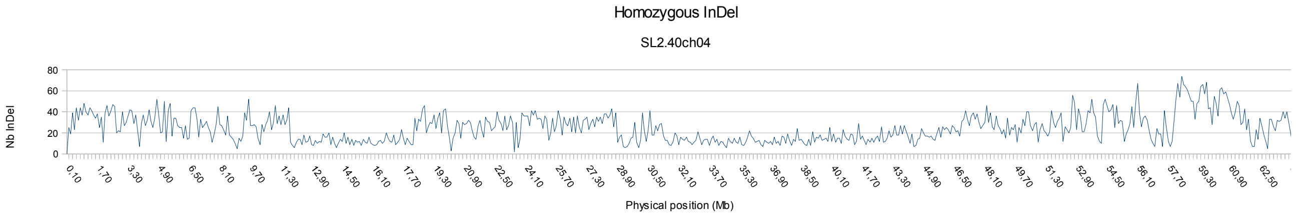
Homozygous InDel
SL2.40ch04
Nb InDel
Physical position (Mb)

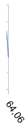
64.06

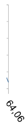
64.06

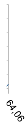
64.06

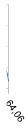
64.06

Cervil - Homozygous InDel

SL2.40ch04

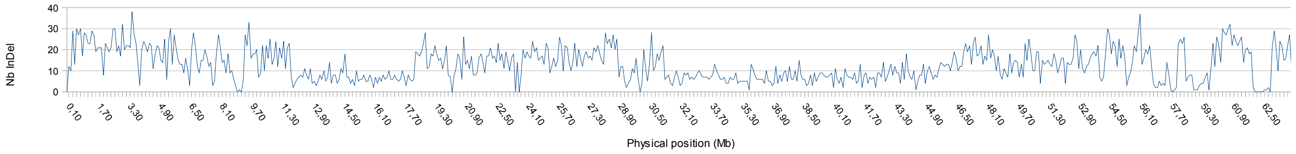

Criollo_new - Homozygous InDel

SL2.40ch04

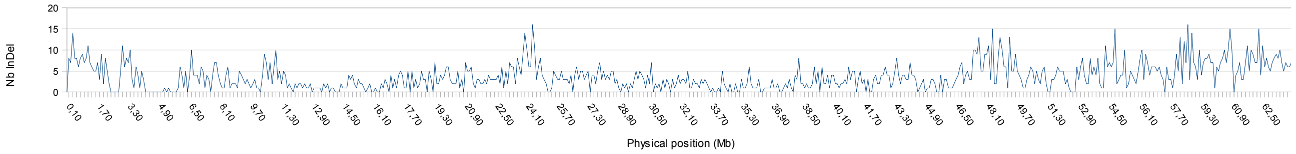

Ferum_new - Homozygous InDel

SL2.40ch04

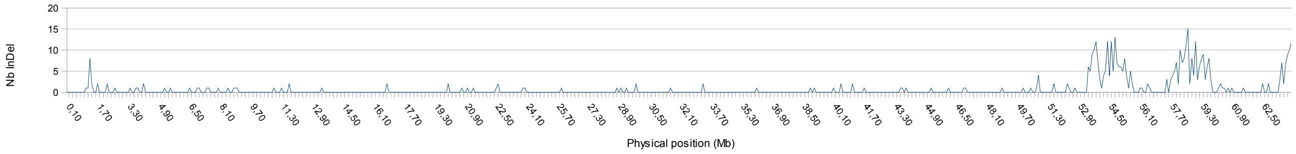

LA0147 - Homozygous InDel

SL2.40ch04

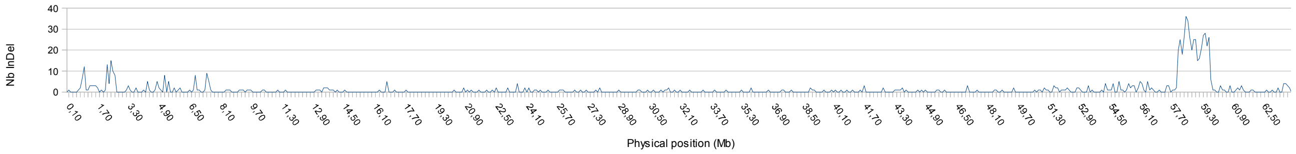

LA1420 - Homozygous InDel

SL2.40ch04

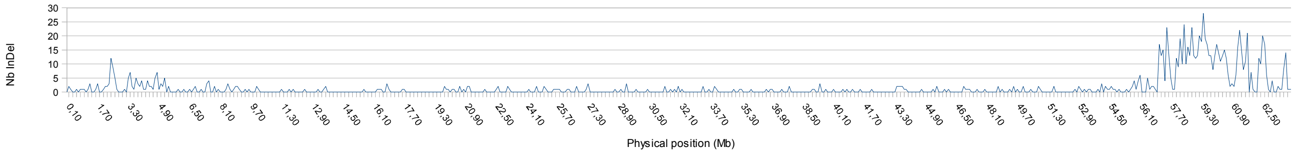

Levovil - Homozygous InDel

SL2.40ch04

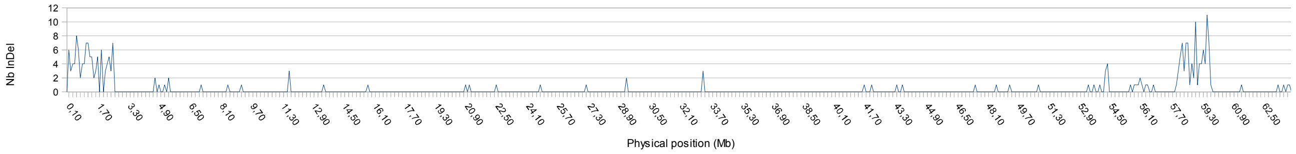

Plovdiv - Homozygous InDel

SL2.40ch04

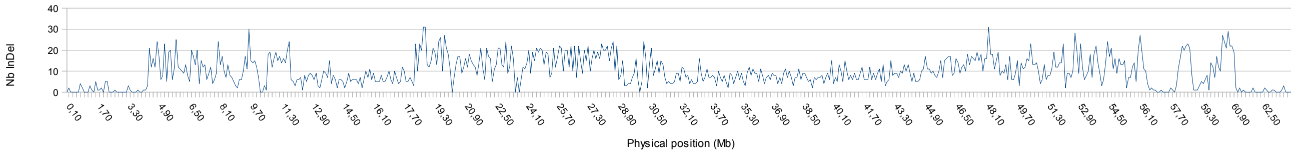

Stupicke - Homozygous InDel

SL2.40ch04

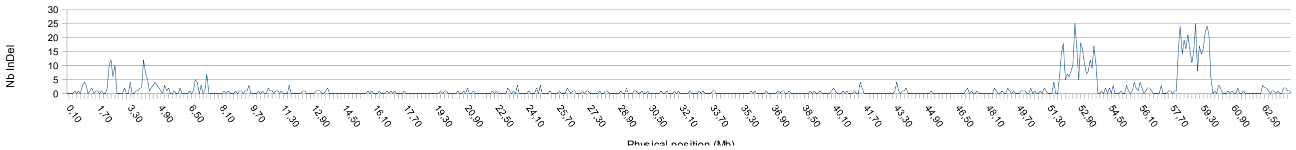

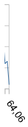
64,06

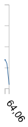
64,06

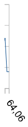
64,06

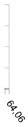
64,06

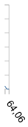
64,06

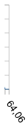
64,06

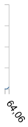
64,06

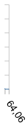
64,06

SNP hmz

Stupicke	0	2	3	4	2	3	1	22	155	191	30	2	21	9	1	32	35	24	29

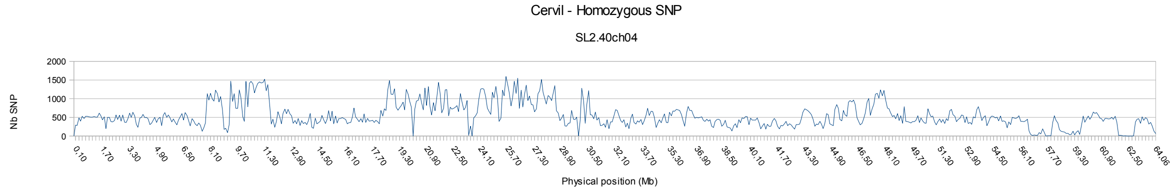

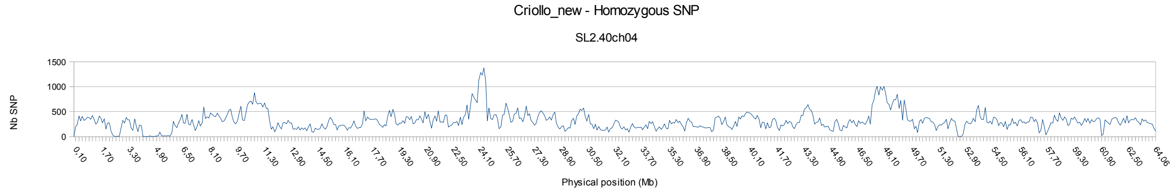

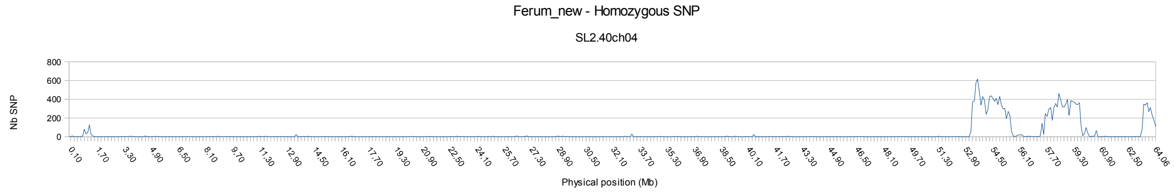

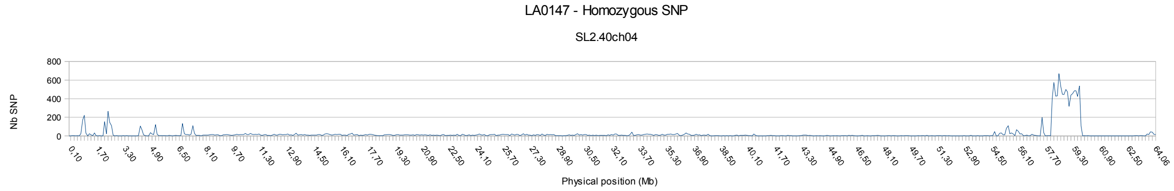

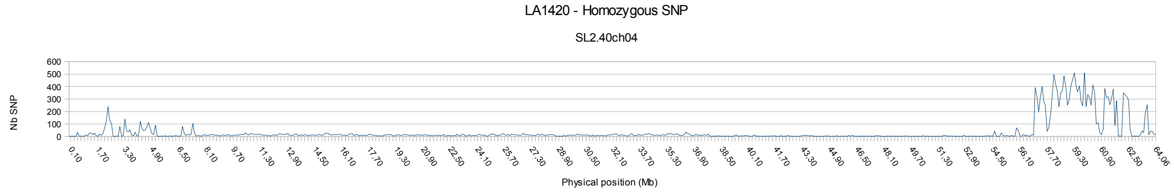

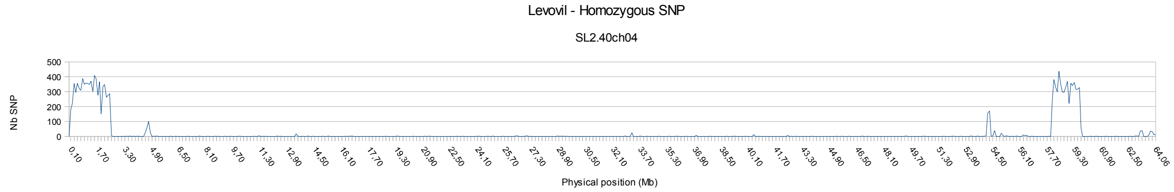

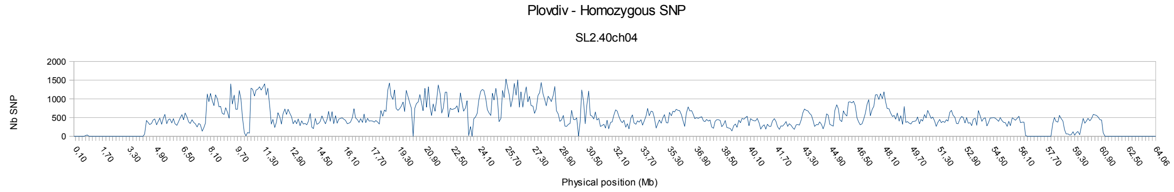

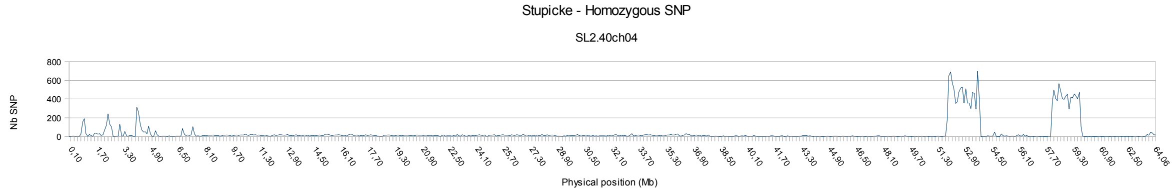

Cervil - Heterozygous SNP

SL2.40ch04

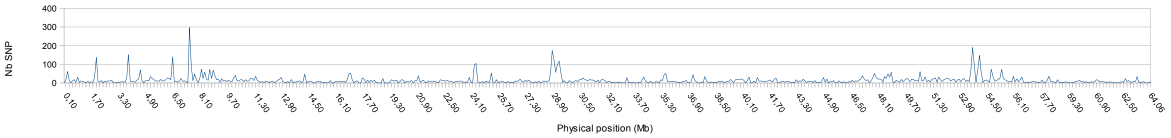

Criollo_new - Heterozygous SNP

SL2.40ch04

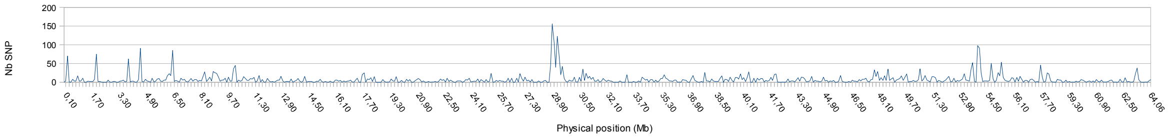

Ferum_new - Heterozygous SNP

SL2.40ch04

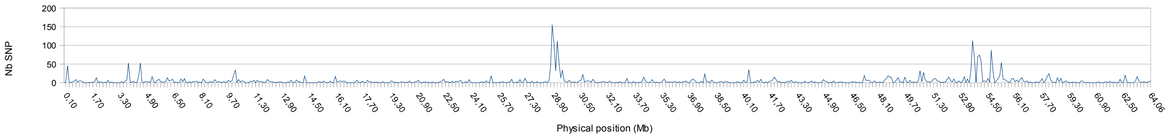

LA0147 - Heterozygous SNP

SL2.40ch04

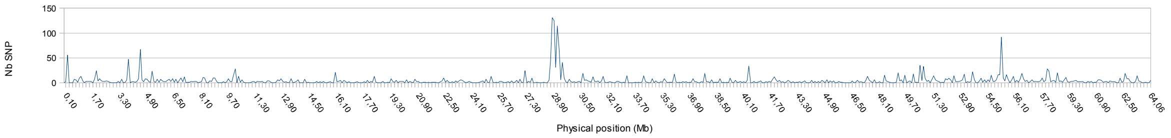

LA1420 - Heterozygous SNP

SL2.40ch04

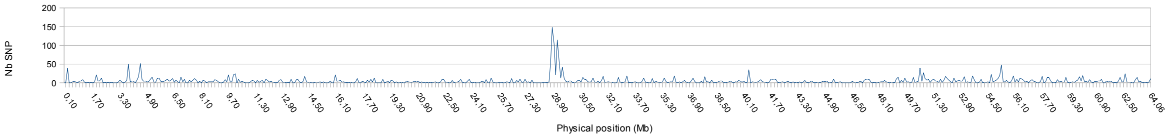

Levovil - Heterozygous SNP

SL2.40ch04

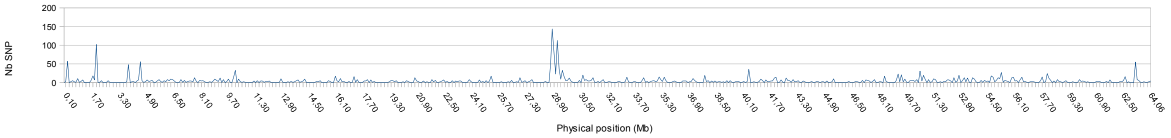

Plovdiv - Heterozygous SNP

SL2.40ch04

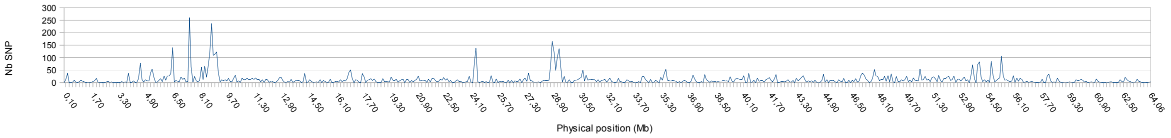

Stupicke - Heterozygous SNP

SL2.40ch04

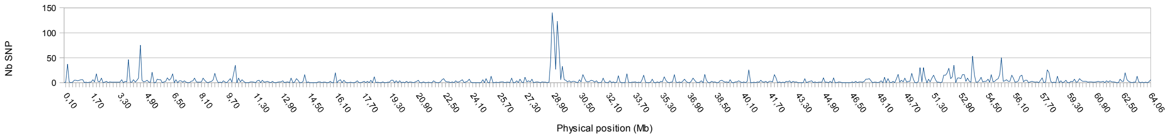

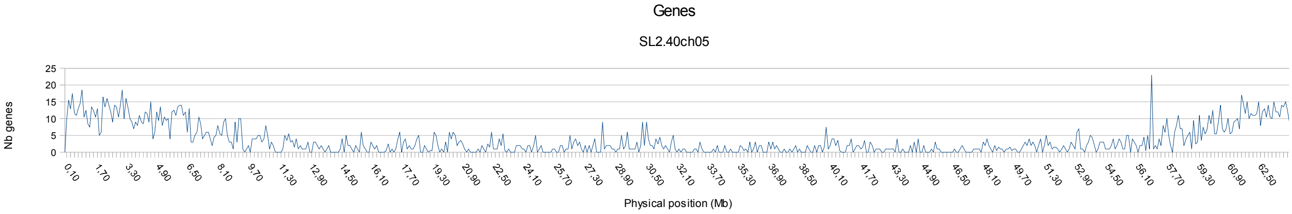
Genes
SL2.40ch05
Nb genes
Physical position (Mb)

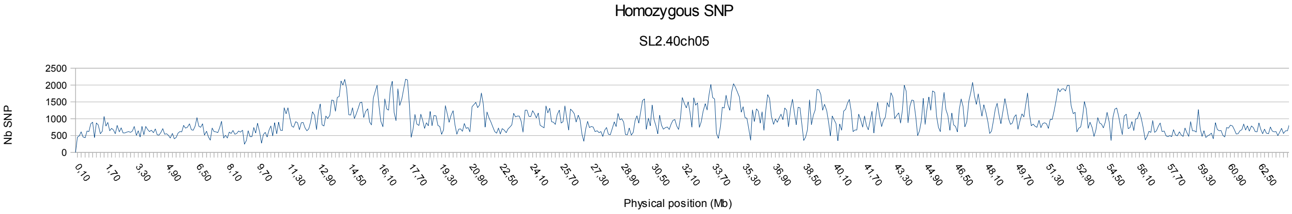
Homozygous SNP
SL2.40ch05
Nb SNP
Physical position (Mb)

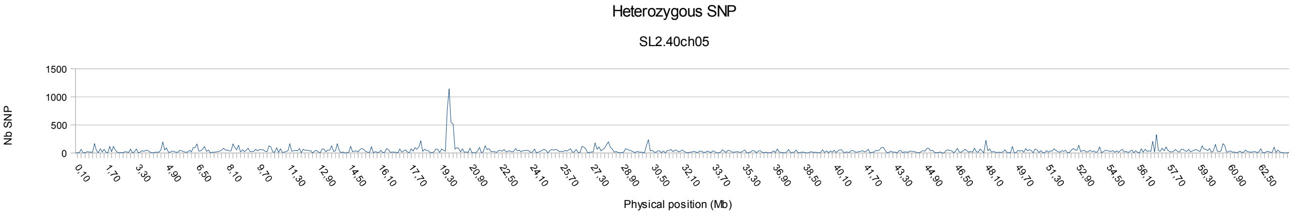
Heterozygous SNP
SL2.40ch05
Nb SNP
Physical position (Mb)

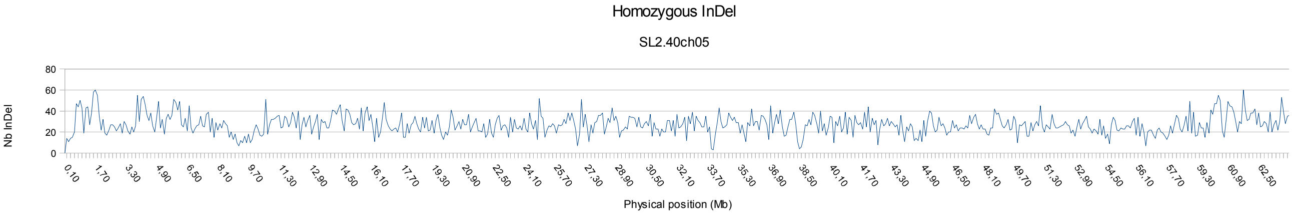
Homozygous InDel
SL2.40ch05
Nb InDel
Physical position (Mb)

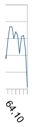
64.10

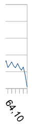
64.10

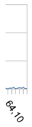
64.10

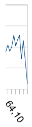
64.10

Cervil - Homozygous InDel

SL2.40ch04

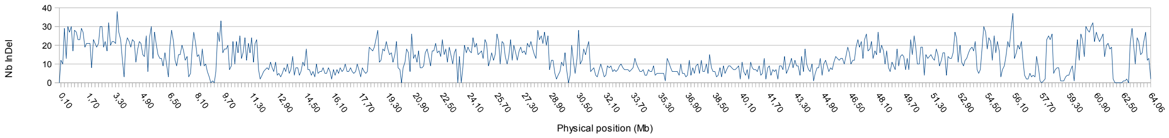

Criollo_new - Homozygous InDel

SL2.40ch04

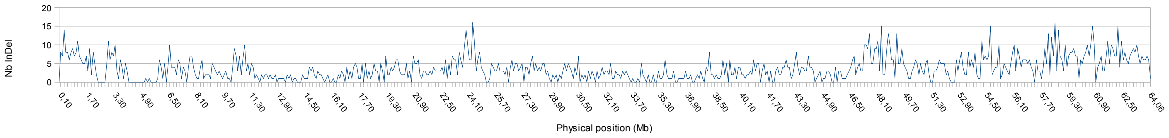

Ferum_new - Homozygous InDel

SL2.40ch04

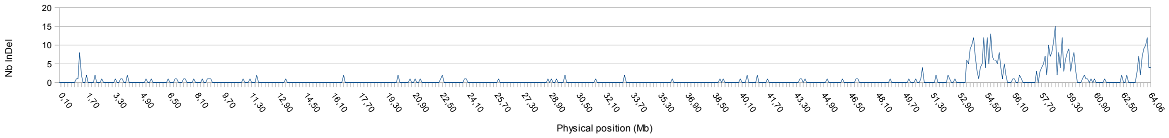

LA0147 - Homozygous InDel

SL2.40ch04

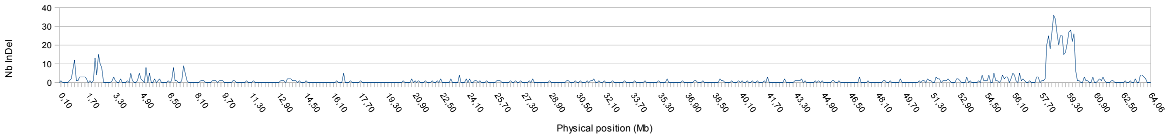

LA1420 - Homozygous InDel

SL2.40ch04

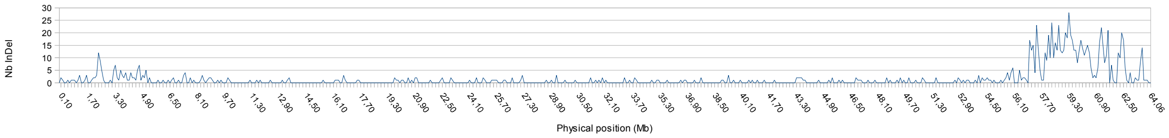

Levovil - Homozygous InDel

SL2.40ch04

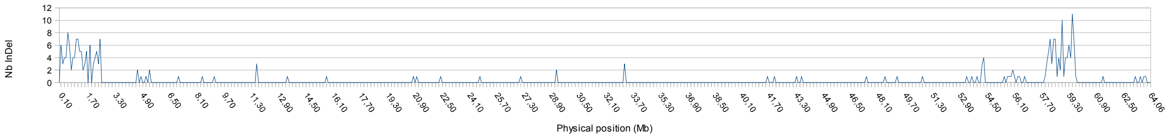

Plovdiv - Homozygous InDel

SL2.40ch04

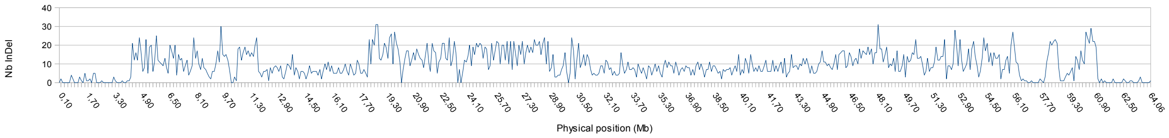

Stupicke - Homozygous InDel

SL2.40ch04

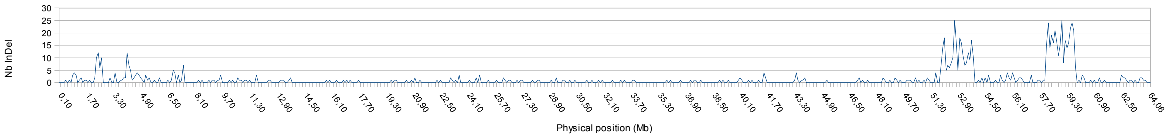

Cervil - Homozygous SNP

SL2.40ch05

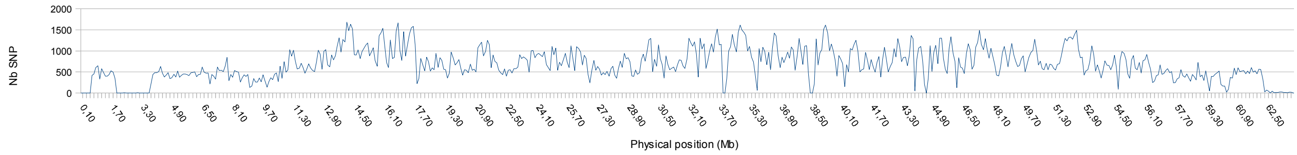

Plovdiv - Homozygous SNP

SL2.40ch05

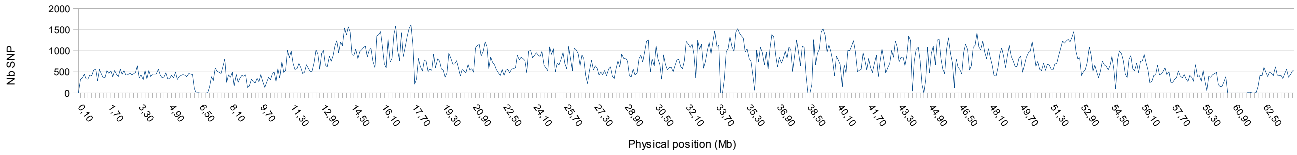

LA1420 - Homozygous SNP

SL2.40ch05

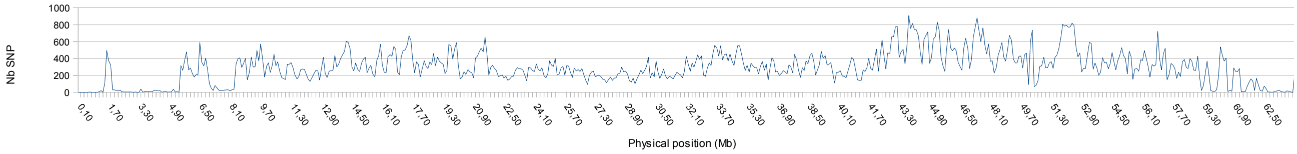

Criollo_new - Homozygous SNP

SL2.40ch05

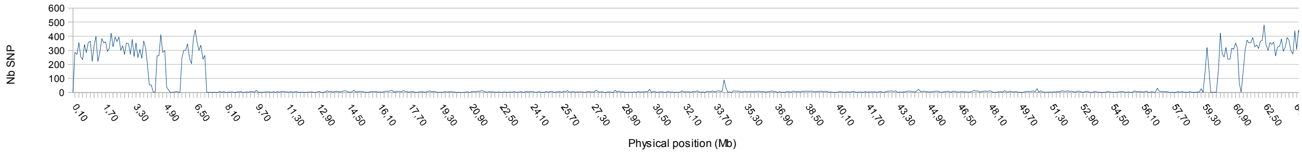

Stupicke - Homozygous SNP

SL2.40ch05

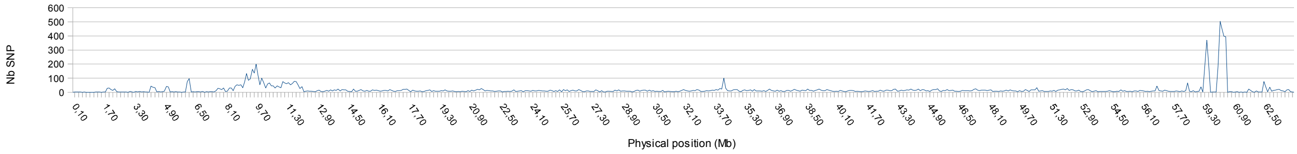

Ferum_new - Homozygous SNP

SL2.40ch05

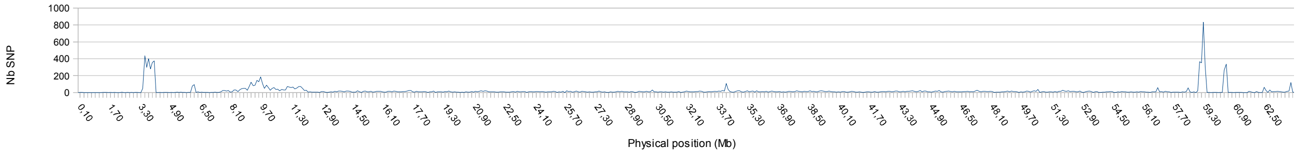

Levovil - Homozygous SNP

SL2.40ch05

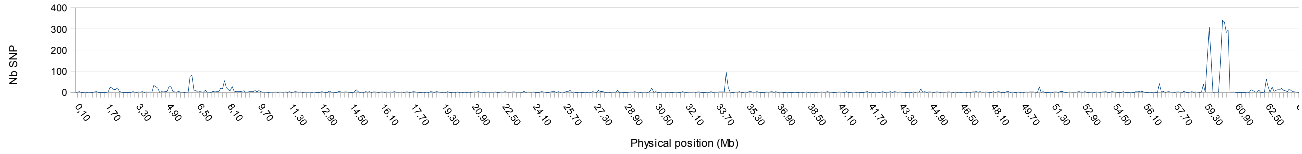

LA0147 - Homozygous SNP

SL2.40ch05

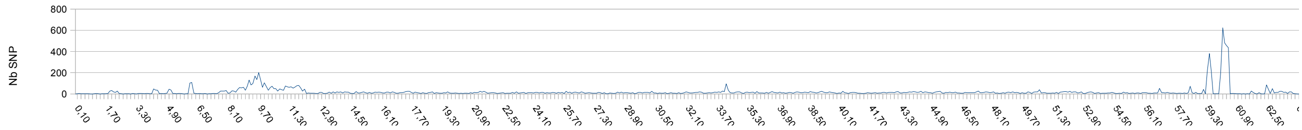

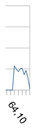
64,10

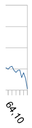
64,10

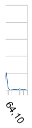
64,10

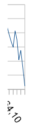

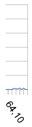
64,10

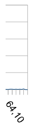
64,10

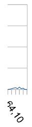

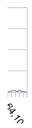

Cervil - Heterozygous SNP

SL2.40ch05

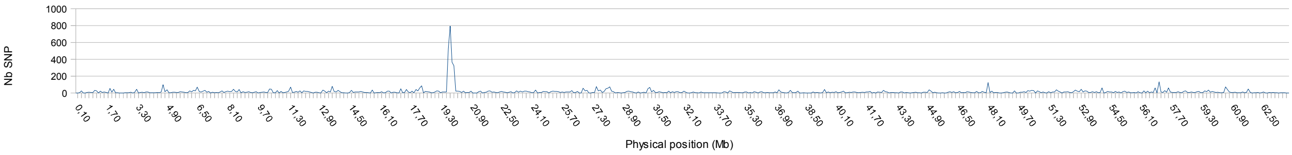

Criollo_new - Heterozygous SNP

SL2.40ch05

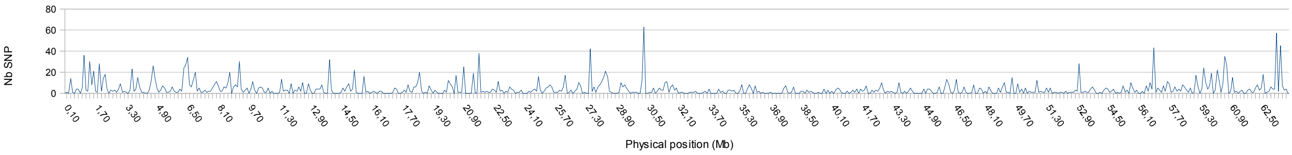

Ferum_new - Heterozygous SNP

SL2.40ch05

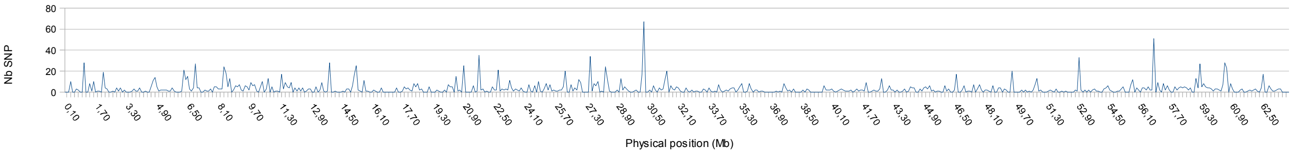

LA0147 - Heterozygous SNP

SL2.40ch05

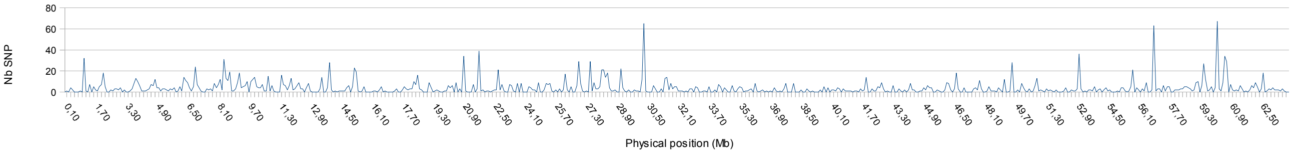

LA1420 - Heterozygous SNP

SL2.40ch05

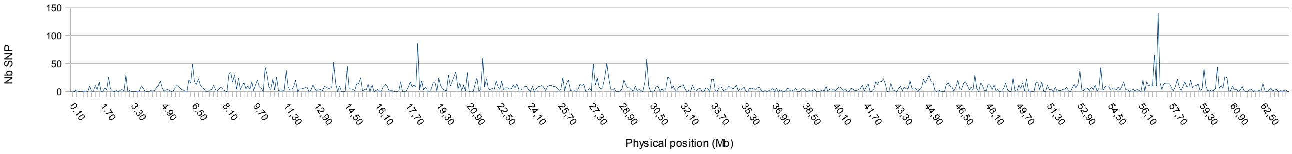

Levovil - Heterozygous SNP

SL2.40ch05

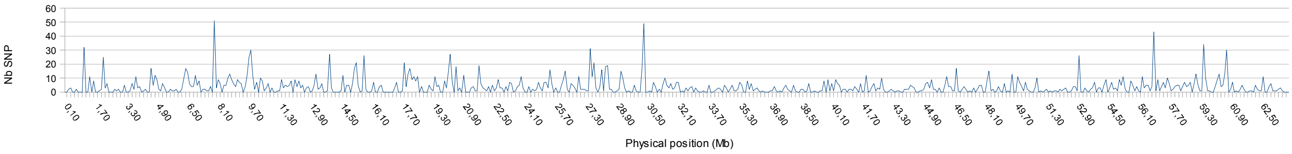

Plovdiv - Heterozygous SNP

SL2.40ch05

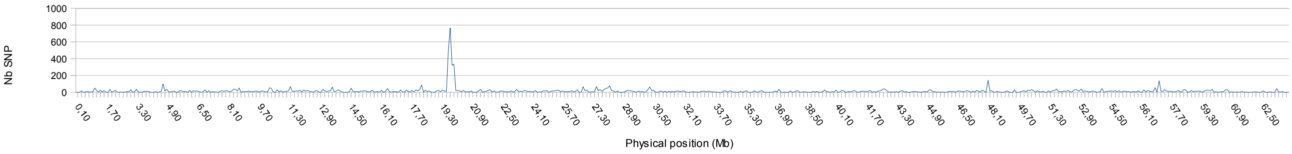

Stupicke - Heterozygous SNP

SL2.40ch05

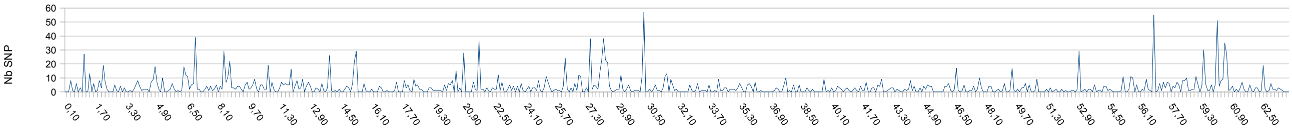

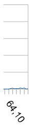
64,10

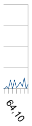
64,10

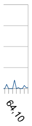
64,10

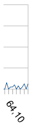
64,10

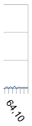
64,10

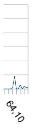
64,10

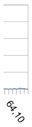
64,10

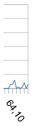
64,10

All_100kb

Genes

SL2.40ch06

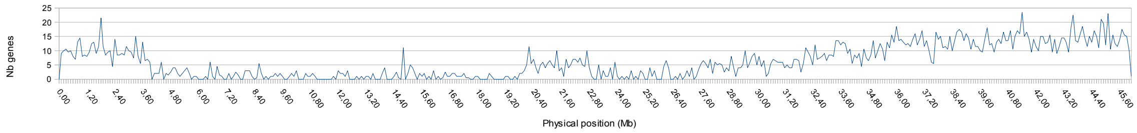

Homozygous SNP

SL2.40ch06

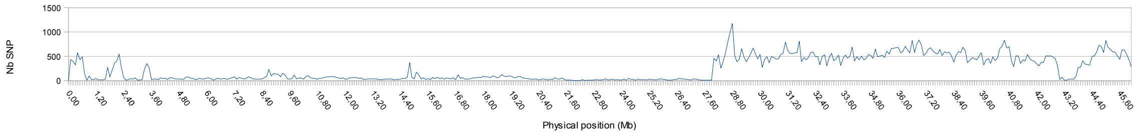

Heterozygous SNP

SL2.40ch06

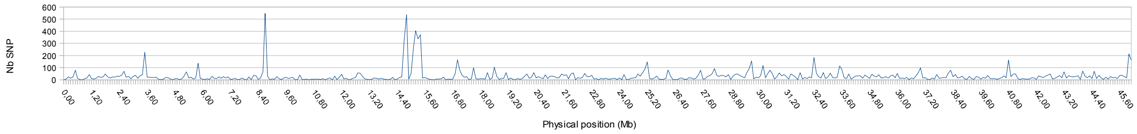

Homozygous InDel

SL2.40ch06

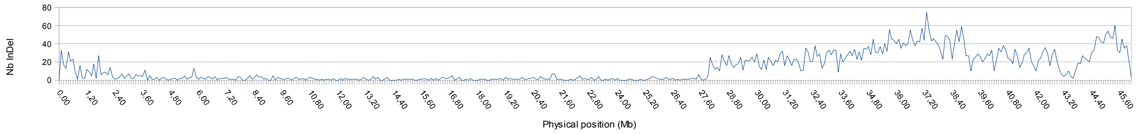

Cervil - Homozygous InDel

SL2.40ch06

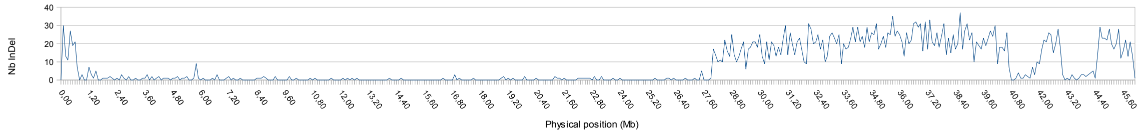

Criollo_new - Homozygous InDel

SL2.40ch06

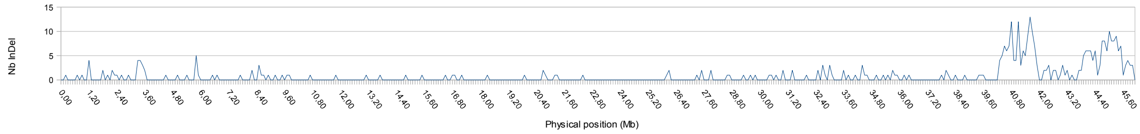

Ferum_new - Homozygous InDel

SL2.40ch06

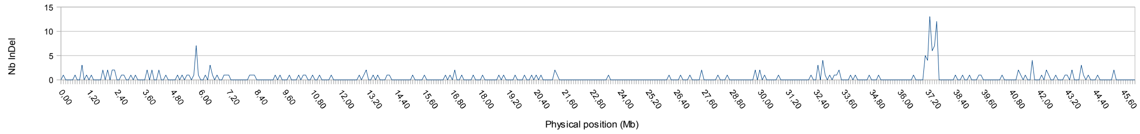

LA0147 - Homozygous InDel

SL2.40ch06

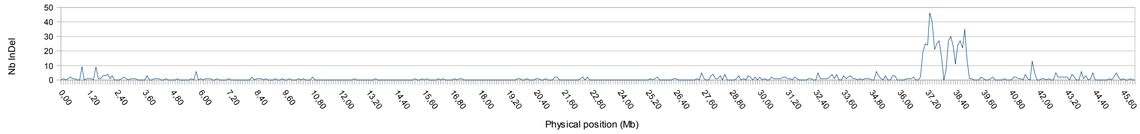

LA1420 - Homozygous InDel

SL2.40ch06

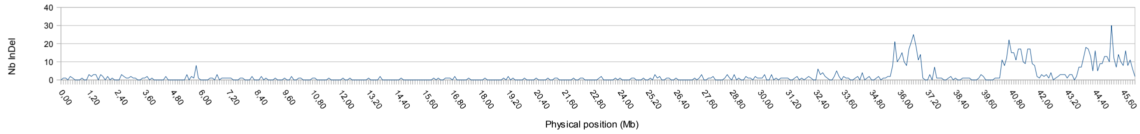

Levovil - Homozygous InDel

SL2.40ch06

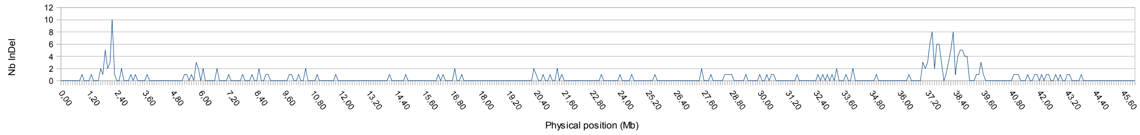

Plovdiv - Homozygous InDel

SL2.40ch06

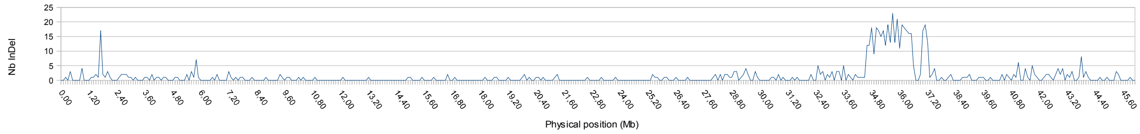

Stupicke - Homozygous InDel

SL2.40ch06

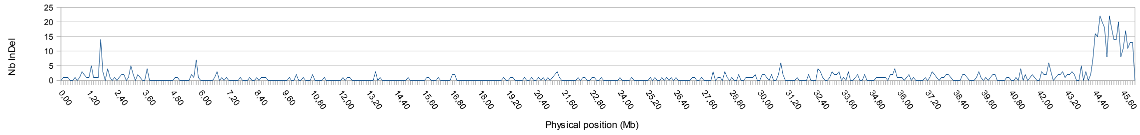

Cervil - Homozygous SNP

SL2.40ch06

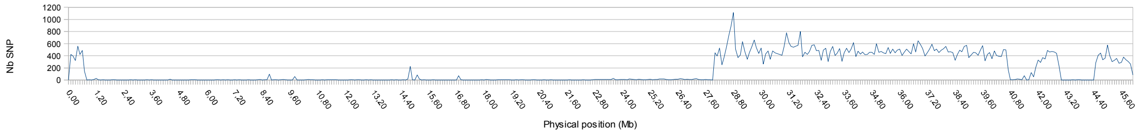

Criollo_new - Homozygous SNP

SL2.40ch06

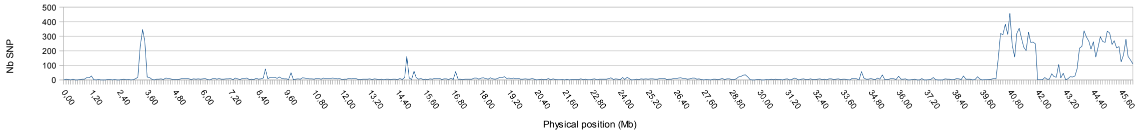

Ferum_new - Homozygous SNP

SL2.40ch06

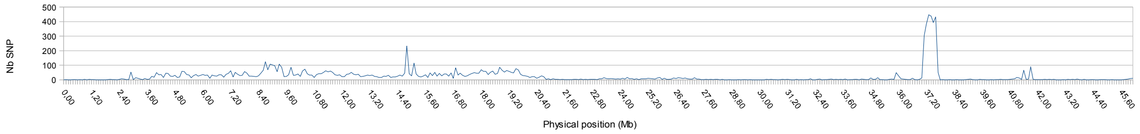

LA0147 - Homozygous SNP

SL2.40ch06

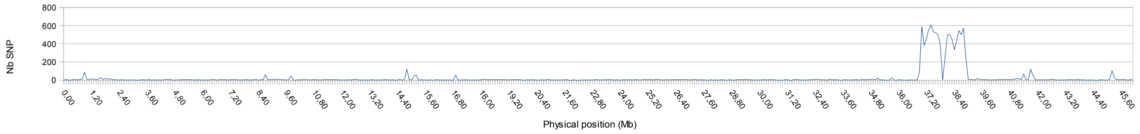

LA1420 - Homozygous SNP

SL2.40ch06

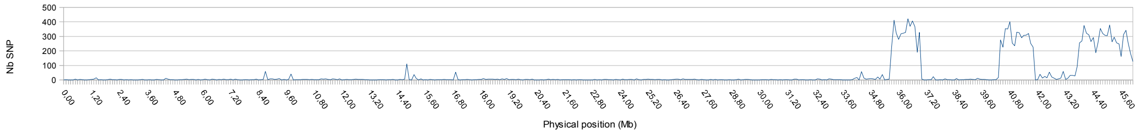

Levovil - Homozygous SNP

SL2.40ch06

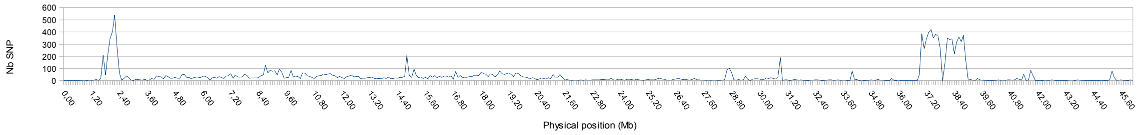

Plovdiv - Homozygous SNP

SL2.40ch06

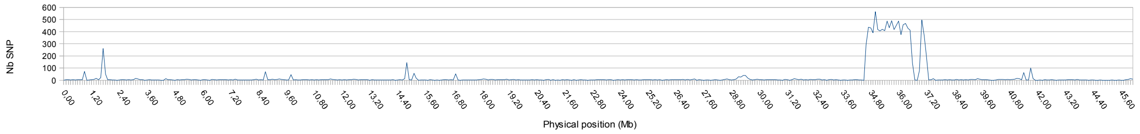

Stupicke - Homozygous SNP

SL2.40ch06

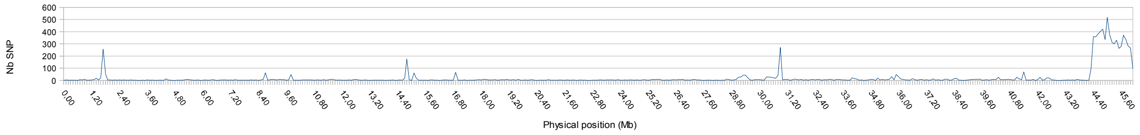

Cervil - Heterozygous SNP

SL2.40ch06

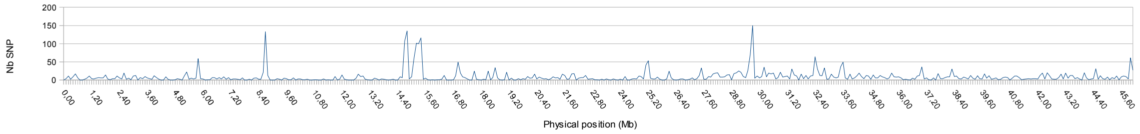

Criollo_new - Heterozygous SNP

SL2.40ch06

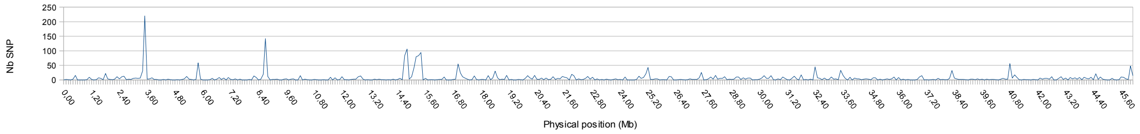

Ferum_new - Heterozygous SNP

SL2.40ch06

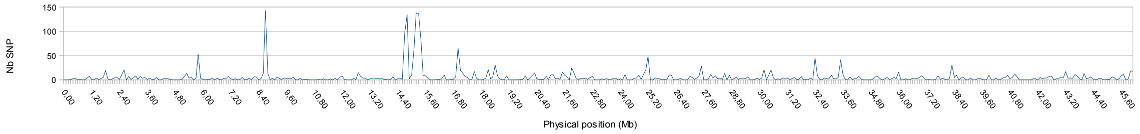

LA0147 - Heterozygous SNP

SL2.40ch06

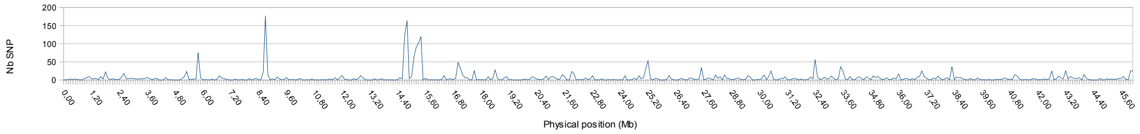

LA1420 - Heterozygous SNP

SL2.40ch06

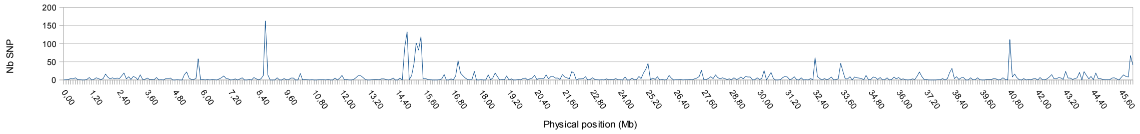

Levovil - Heterozygous SNP

SL2.40ch06

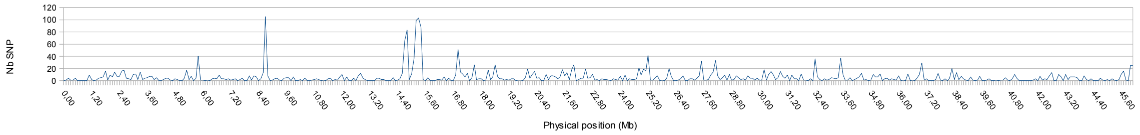

Plovdiv - Heterozygous SNP

SL2.40ch06

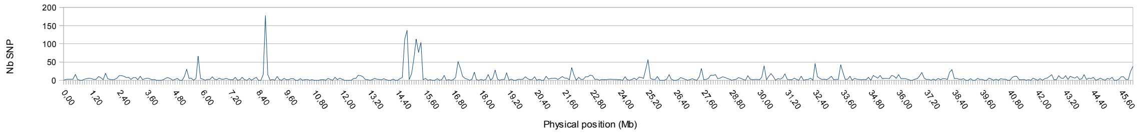

Stupicke - Heterozygous SNP

SL2.40ch06

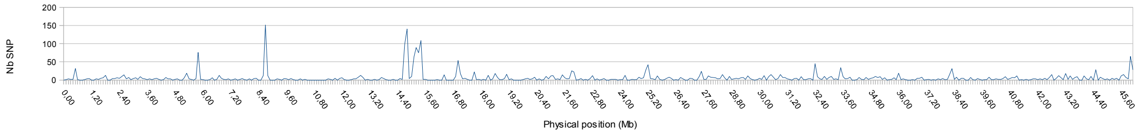

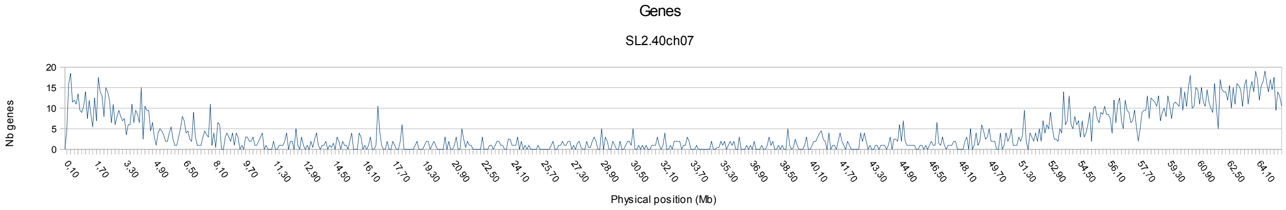
Genes
SL2.40ch07
Nb genes
Physical position (Mb)

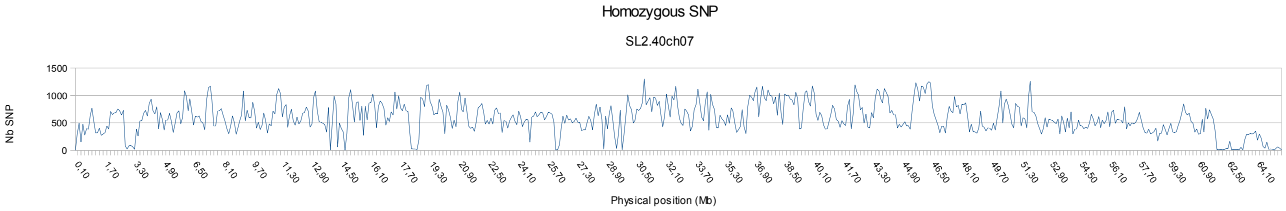
Homozygous SNP
SL2.40ch07
Nb SNP
Physical position (Mb)

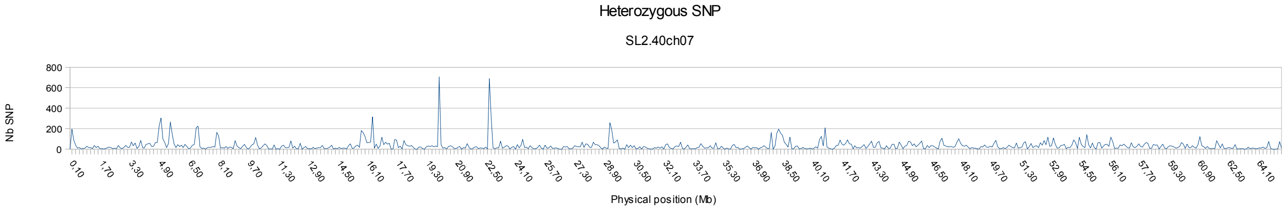
Heterozygous SNP
SL2.40ch07
Nb SNP
Physical position (Mb)

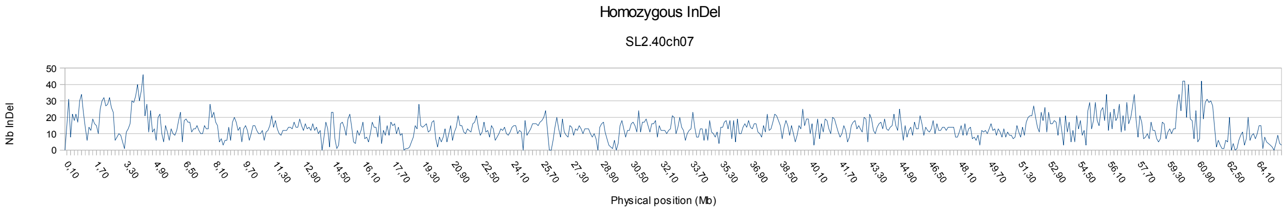
Homozygous InDel
SL2.40ch07
Nb InDel
Physical position (Mb)

Cervil - Homozygous InDel

SL2.40ch07

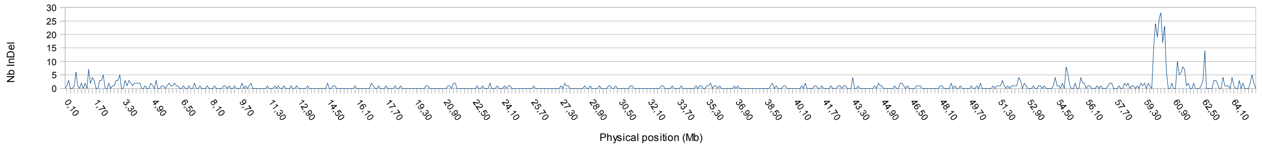

Criollo_new - Homozygous InDel

SL2.40ch07

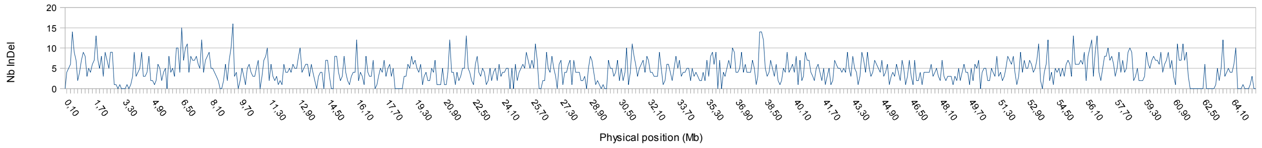

Ferum_new - Homozygous InDel

SL2.40ch07

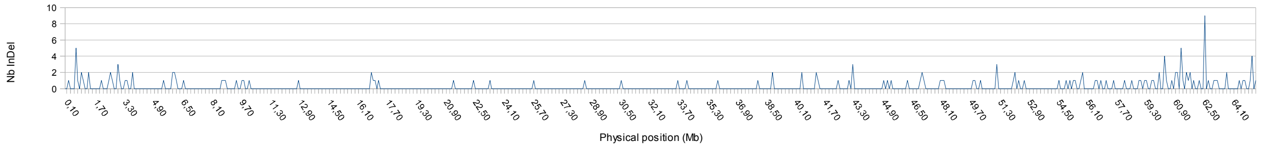

LA0147 - Homozygous InDel

SL2.40ch07

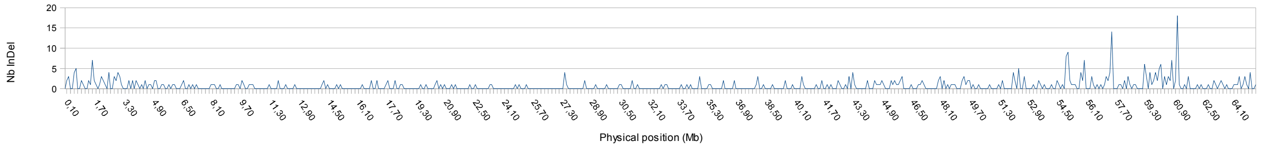

LA1420 - Homozygous InDel

SL2.40ch07

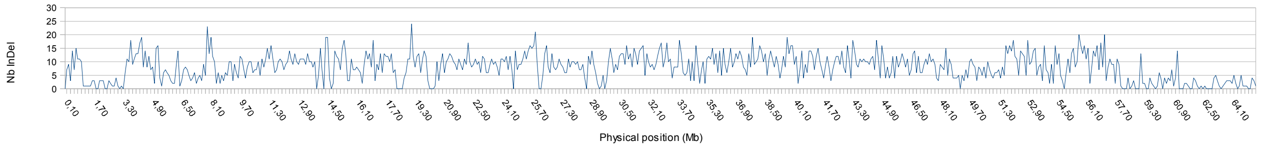

Levovil - Homozygous InDel

SL2.40ch07

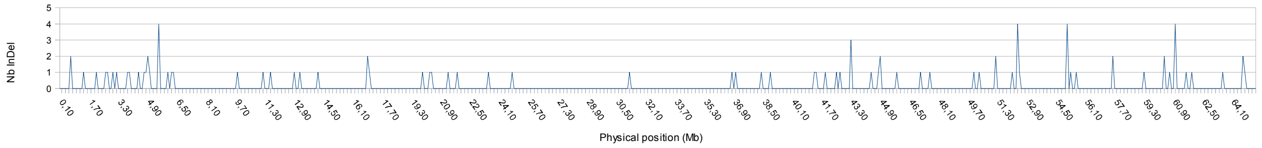

Plovdiv - Homozygous InDel

SL2.40ch07

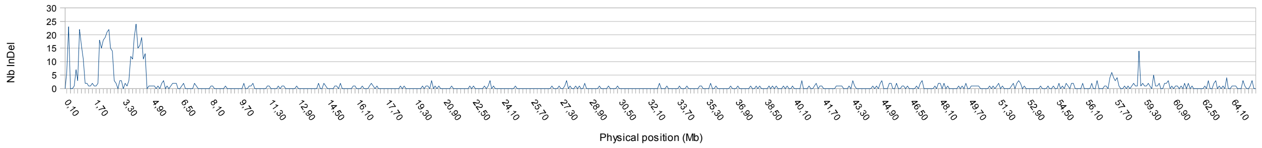

Stupicke - Homozygous InDel

SL2.40ch07

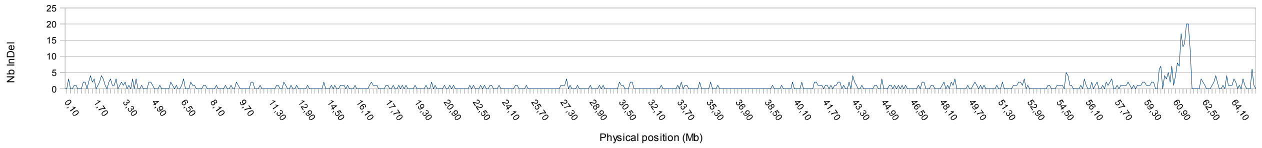

Cervil - Homozygous SNP

SL2.40ch07

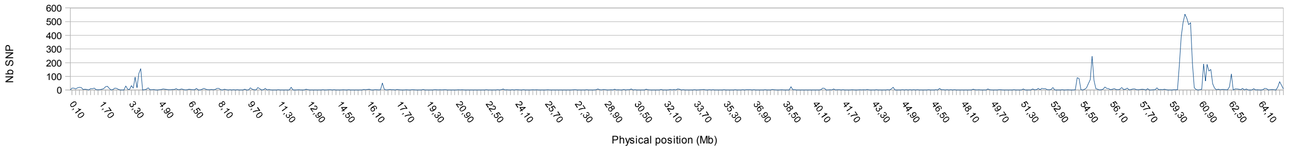

Criollo_new - Homozygous SNP

SL2.40ch07

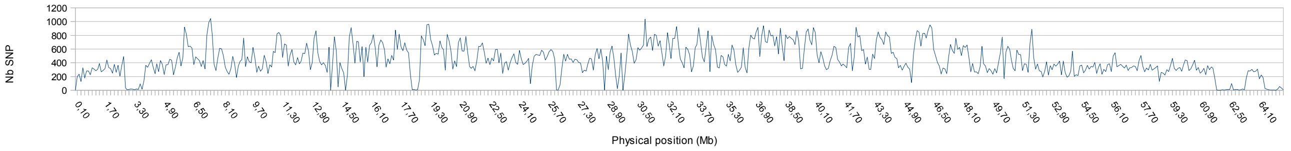

Ferum_new - Homozygous SNP

SL2.40ch07

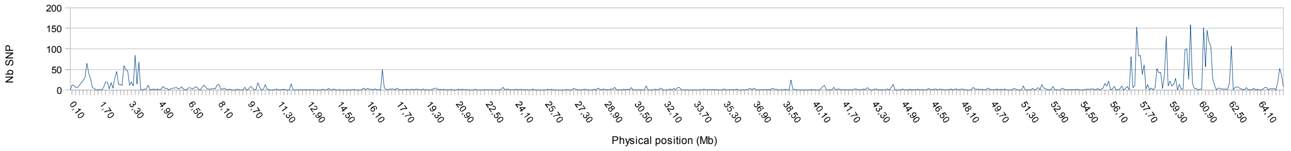

LA0147 - Homozygous SNP

SL2.40ch07

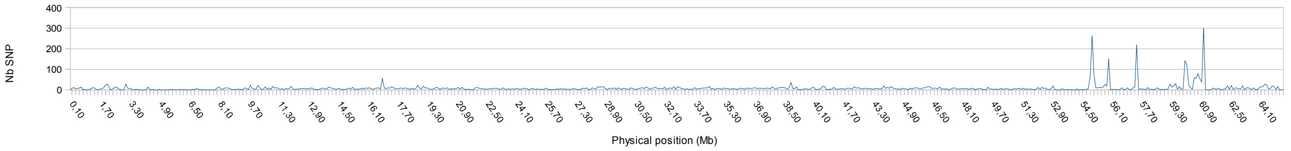

LA1420 - Homozygous SNP

SL2.40ch07

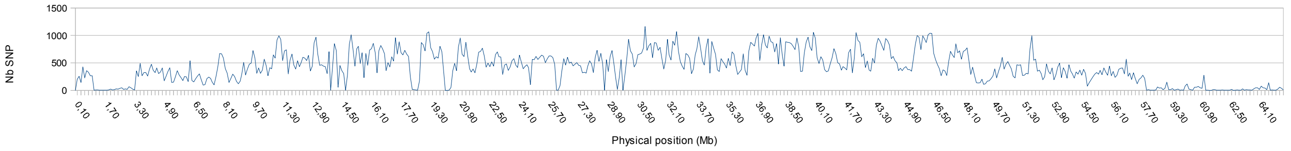

Levovil - Homozygous SNP

SL2.40ch07

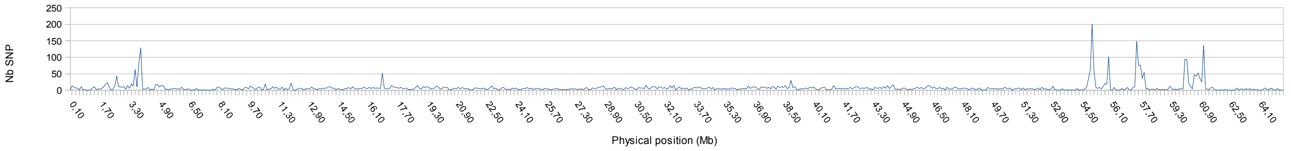

Plovdiv - Homozygous SNP

SL2.40ch07

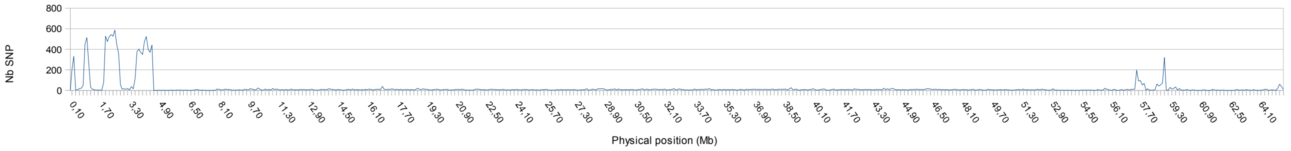

Stupicke - Homozygous SNP

SL2.40ch07

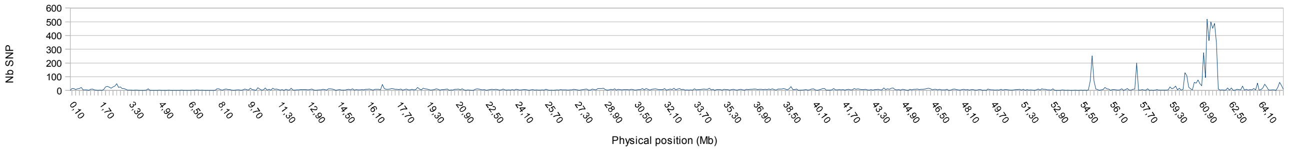

Cervil - Heterozygous SNP

SL2.40ch07

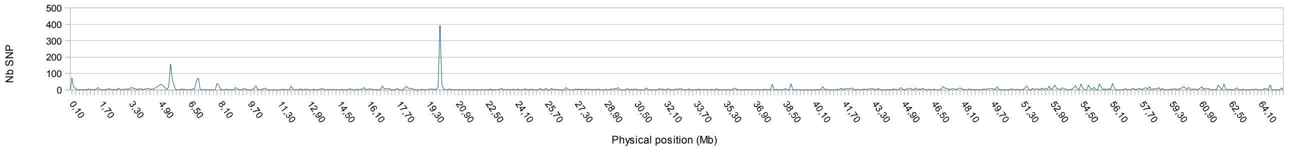

Criollo_new - Heterozygous SNP

SL2.40ch07

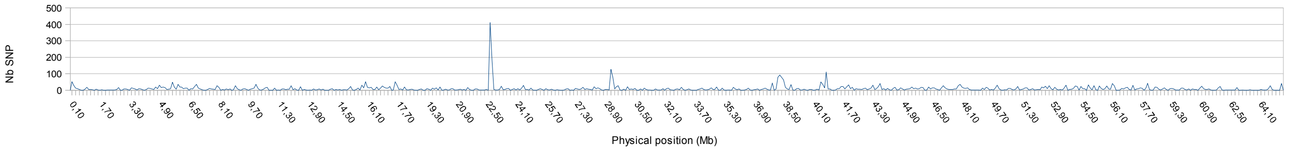

Ferum_new - Heterozygous SNP

SL2.40ch07

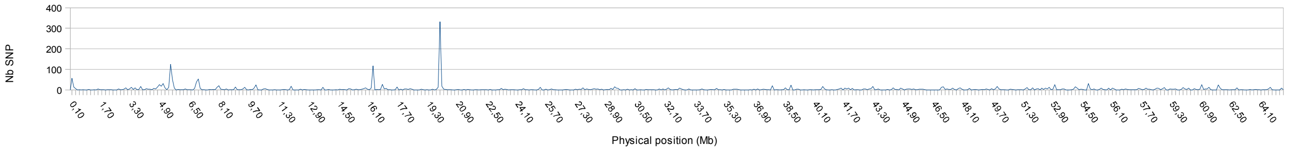

LA0147 - Heterozygous SNP

SL2.40ch07

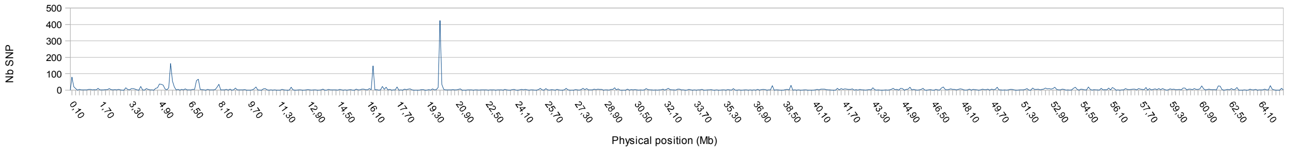

LA1420 - Heterozygous SNP

SL2.40ch07

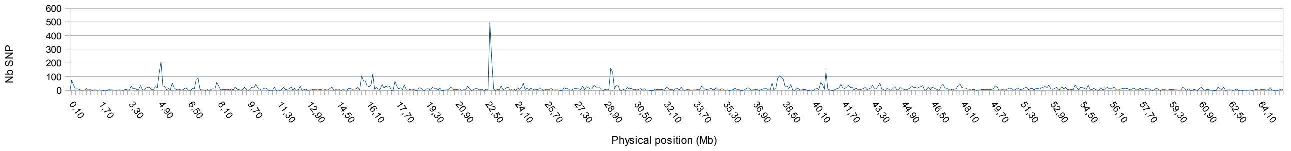

Levovil - Heterozygous SNP

SL2.40ch07

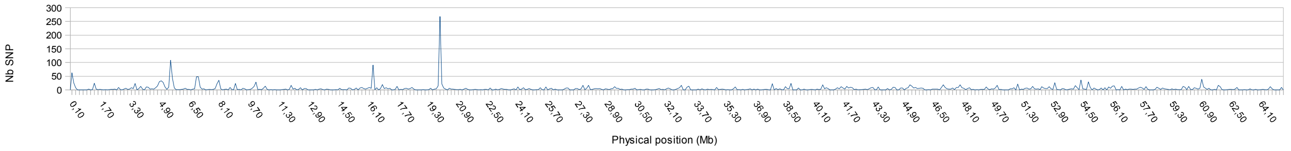

Plovdiv - Heterozygous SNP

SL2.40ch07

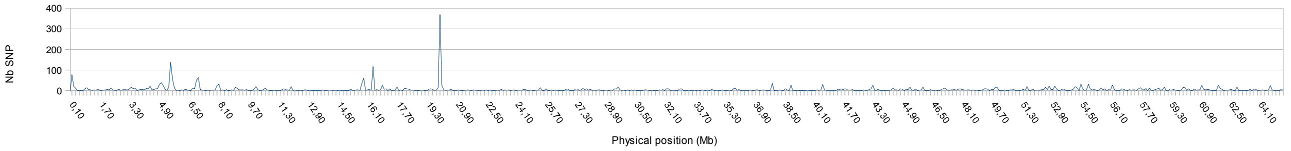

Stupicke - Heterozygous SNP

SL2.40ch07

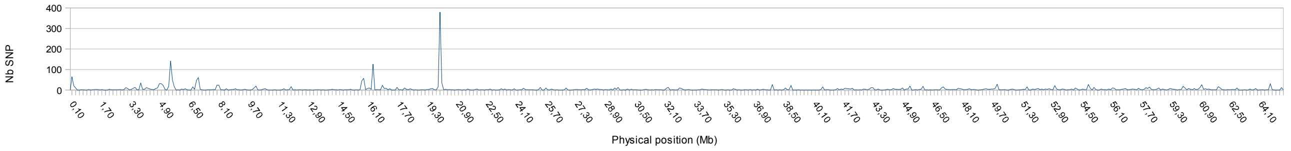

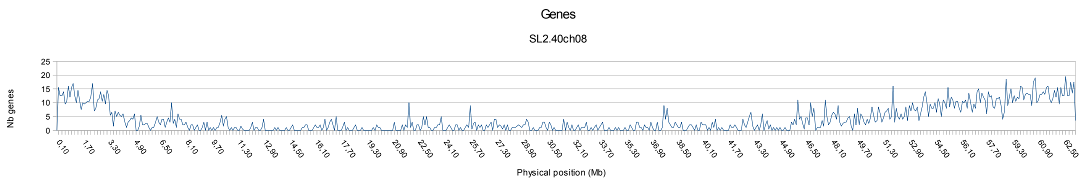
Genes
SL2.40ch08
Nb genes
0
5
10
15
20
25
Physical position (Mb)

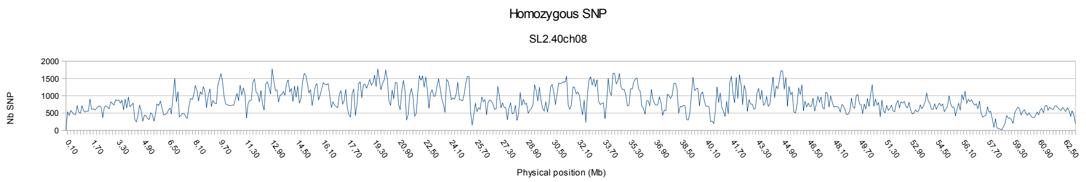
Homozygous SNP
SL2.40ch08
Nb SNP
0
500
1000
1500
2000
Physical position (Mb)

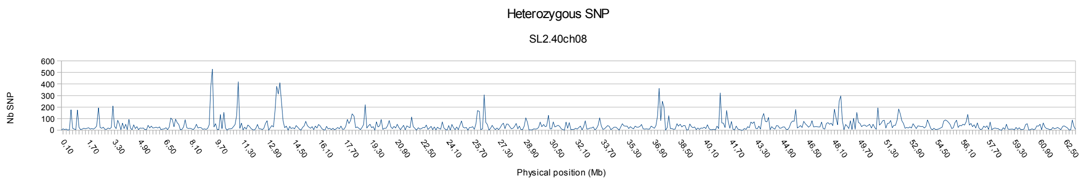
Heterozygous SNP
SL2.40ch08
Nb SNP
0
100
200
300
400
500
600
Physical position (Mb)

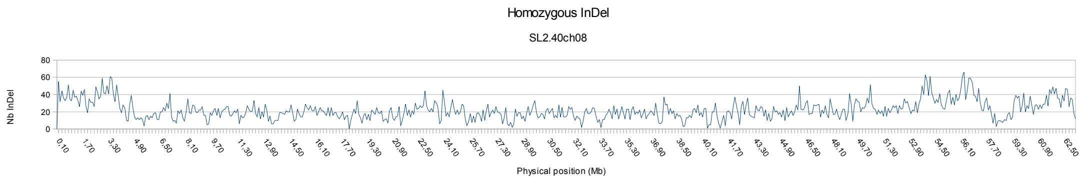
Homozygous InDel
SL2.40ch08
Nb InDel
0
20
40
60
80
Physical position (Mb)

Cervil - Homozygous InDel

SL2.40ch08

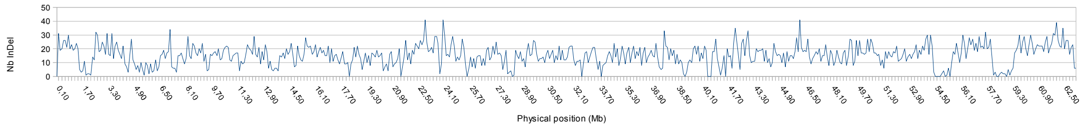

Criollo_new - Homozygous InDel

SL2.40ch08

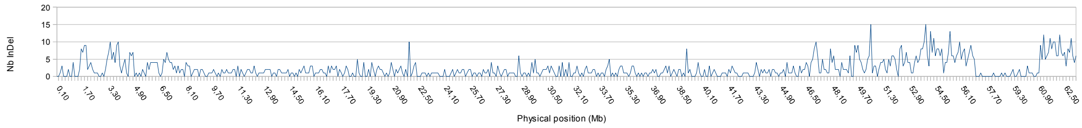

Ferum_new - Homozygous InDel

SL2.40ch08

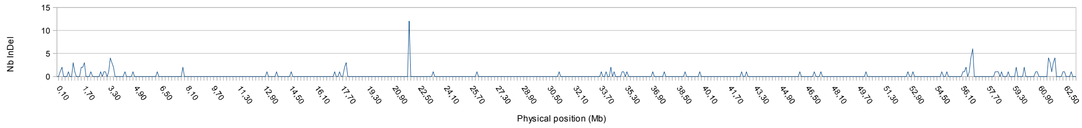

LA0147 - Homozygous InDel

SL2.40ch08

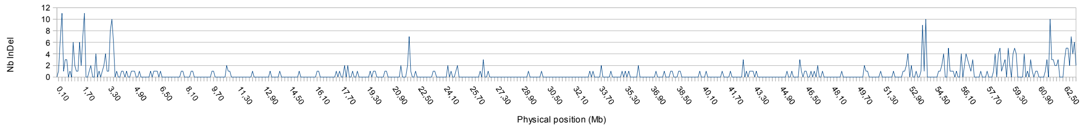

LA1420 - Homozygous InDel

SL2.40ch08

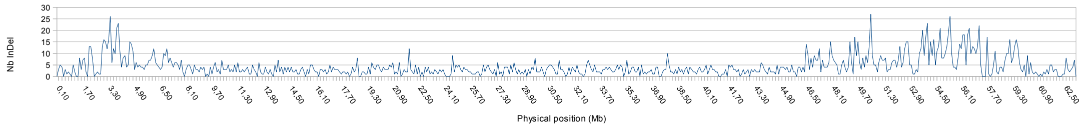

Levovil - Homozygous InDel

SL2.40ch08

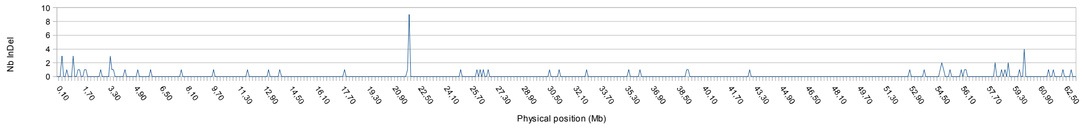

Plovdiv - Homozygous InDel

SL2.40ch08

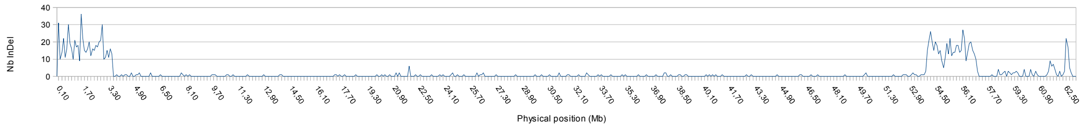

Stupicke - Homozygous InDel

SL2.40ch08

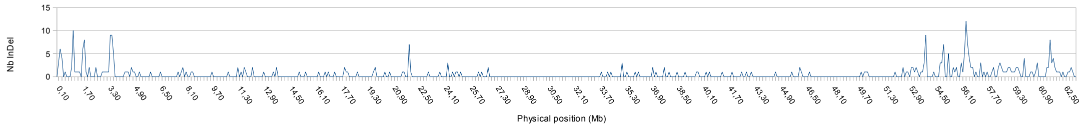

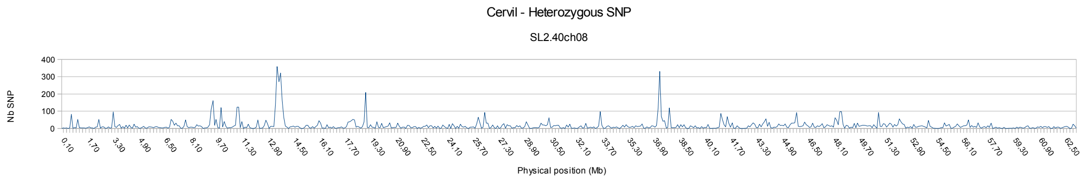
Cervil - Heterozygous SNP
SL2.40ch08
Nb SNP
0
100
200
300
400
0.10 1.70 3.30 4.90 6.50 8.10 9.70 11.30 12.90 14.50 16.10 17.70 19.30 20.90 22.50 24.10 25.70 27.30 28.90 30.50 32.10 33.70 35.30 36.90 38.50 40.10 41.70 43.30 44.90 46.50 48.10 49.70 51.30 52.90 54.50 56.10 57.70 59.30 60.90 62.50
Physical position (Mb)

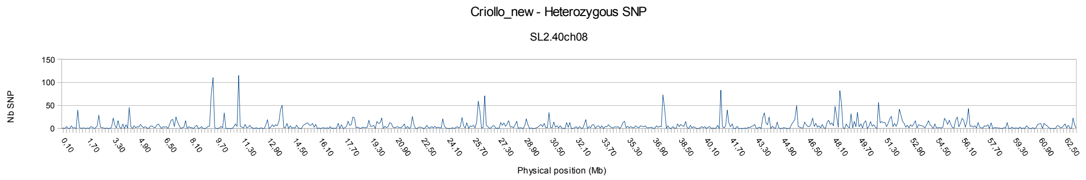
Criollo_new - Heterozygous SNP
SL2.40ch08
Nb SNP
0
50
100
150
0.10 1.70 3.30 4.90 6.50 8.10 9.70 11.30 12.90 14.50 16.10 17.70 19.30 20.90 22.50 24.10 25.70 27.30 28.90 30.50 32.10 33.70 35.30 36.90 38.50 40.10 41.70 43.30 44.90 46.50 48.10 49.70 51.30 52.90 54.50 56.10 57.70 59.30 60.90 62.50
Physical position (Mb)

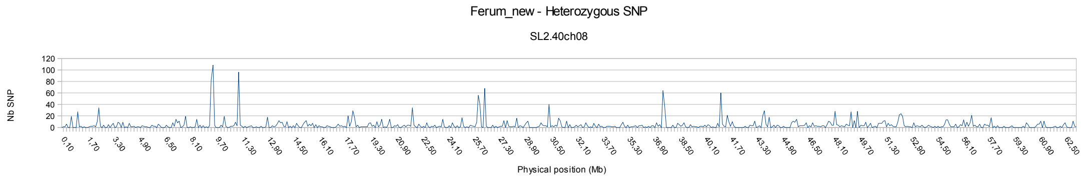
Ferum_new - Heterozygous SNP
SL2.40ch08
Nb SNP
0
20
40
60
80
100
120
0.10 1.70 3.30 4.90 6.50 8.10 9.70 11.30 12.90 14.50 16.10 17.70 19.30 20.90 22.50 24.10 25.70 27.30 28.90 30.50 32.10 33.70 35.30 36.90 38.50 40.10 41.70 43.30 44.90 46.50 48.10 49.70 51.30 52.90 54.50 56.10 57.70 59.30 60.90 62.50
Physical position (Mb)

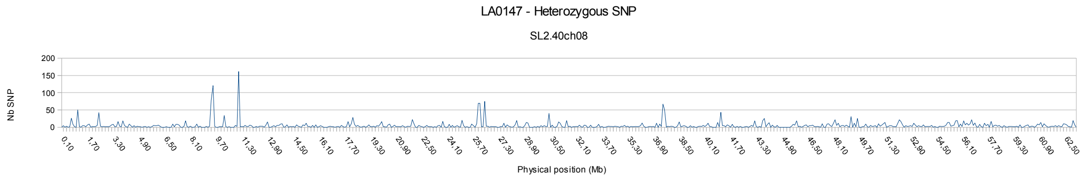
LA0147 - Heterozygous SNP
SL2.40ch08
Nb SNP
0
50
100
150
200
0.10 1.70 3.30 4.90 6.50 8.10 9.70 11.30 12.90 14.50 16.10 17.70 19.30 20.90 22.50 24.10 25.70 27.30 28.90 30.50 32.10 33.70 35.30 36.90 38.50 40.10 41.70 43.30 44.90 46.50 48.10 49.70 51.30 52.90 54.50 56.10 57.70 59.30 60.90 62.50
Physical position (Mb)

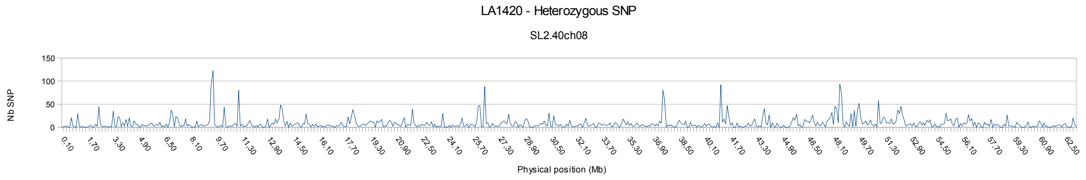
LA1420 - Heterozygous SNP
SL2.40ch08
Nb SNP
0
50
100
150
0.10 1.70 3.30 4.90 6.50 8.10 9.70 11.30 12.90 14.50 16.10 17.70 19.30 20.90 22.50 24.10 25.70 27.30 28.90 30.50 32.10 33.70 35.30 36.90 38.50 40.10 41.70 43.30 44.90 46.50 48.10 49.70 51.30 52.90 54.50 56.10 57.70 59.30 60.90 62.50
Physical position (Mb)

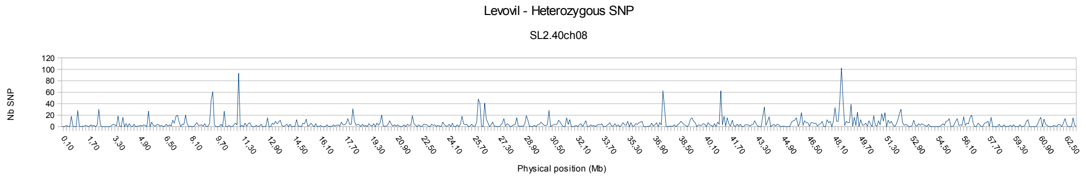
Levovil - Heterozygous SNP
SL2.40ch08
Nb SNP
0
20
40
60
80
100
120
0.10 1.70 3.30 4.90 6.50 8.10 9.70 11.30 12.90 14.50 16.10 17.70 19.30 20.90 22.50 24.10 25.70 27.30 28.90 30.50 32.10 33.70 35.30 36.90 38.50 40.10 41.70 43.30 44.90 46.50 48.10 49.70 51.30 52.90 54.50 56.10 57.70 59.30 60.90 62.50
Physical position (Mb)

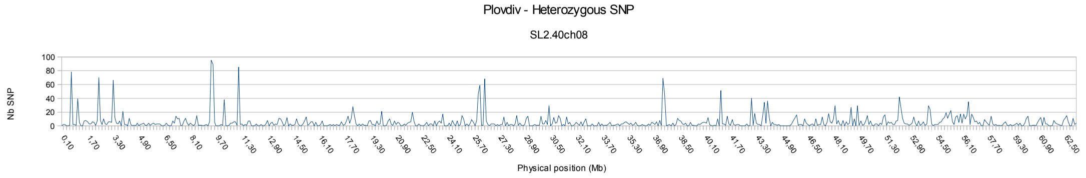
Plovdiv - Heterozygous SNP
SL2.40ch08
Nb SNP
0
20
40
60
80
100
0.10 1.70 3.30 4.90 6.50 8.10 9.70 11.30 12.90 14.50 16.10 17.70 19.30 20.90 22.50 24.10 25.70 27.30 28.90 30.50 32.10 33.70 35.30 36.90 38.50 40.10 41.70 43.30 44.90 46.50 48.10 49.70 51.30 52.90 54.50 56.10 57.70 59.30 60.90 62.50
Physical position (Mb)

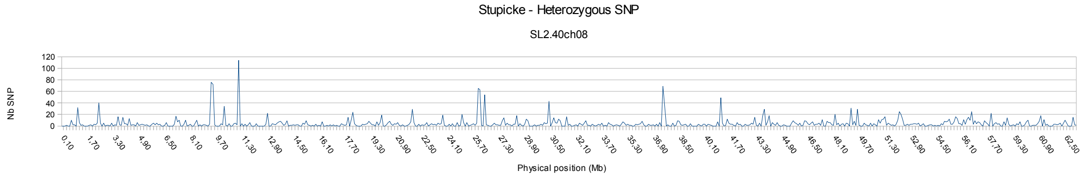
Stupicke - Heterozygous SNP
SL2.40ch08
Nb SNP
0
20
40
60
80
100
120
0.10 1.70 3.30 4.90 6.50 8.10 9.70 11.30 12.90 14.50 16.10 17.70 19.30 20.90 22.50 24.10 25.70 27.30 28.90 30.50 32.10 33.70 35.30 36.90 38.50 40.10 41.70 43.30 44.90 46.50 48.10 49.70 51.30 52.90 54.50 56.10 57.70 59.30 60.90 62.50
Physical position (Mb)

Cervil - Homozygous SNP

SL2.40ch08

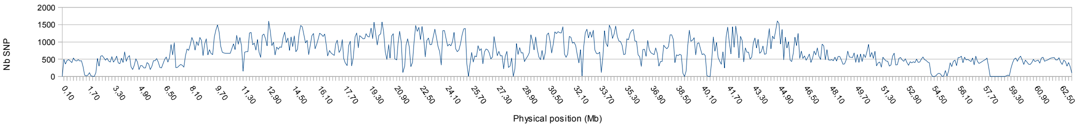

Criollo_new - Homozygous SNP

SL2.40ch08

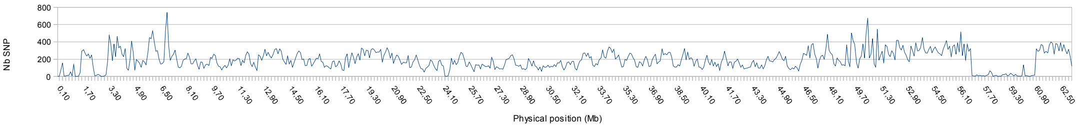

Ferum_new - Homozygous SNP

SL2.40ch08

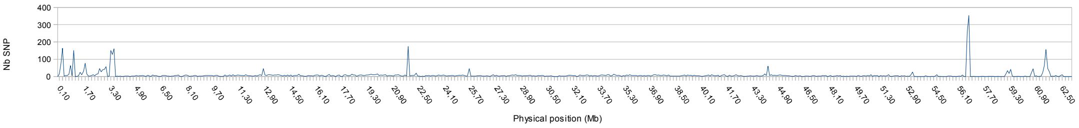

LA0147 - Homozygous SNP

SL2.40ch08

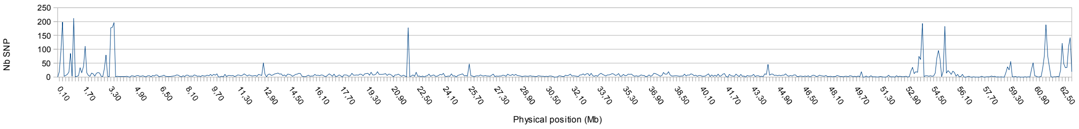

LA1420 - Homozygous SNP

SL2.40ch08

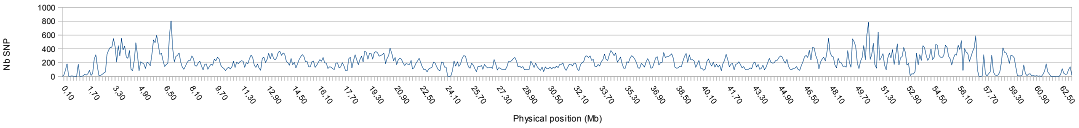

Levovil - Homozygous SNP

SL2.40ch08

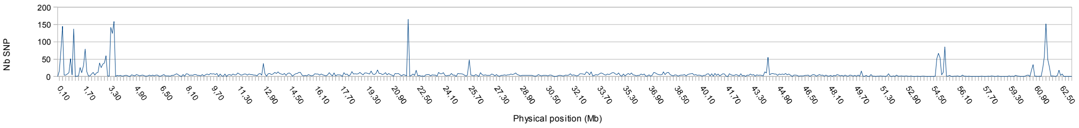

Plovdiv - Homozygous SNP

SL2.40ch08

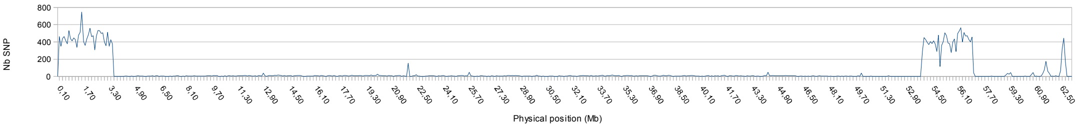

Stupicke - Homozygous SNP

SL2.40ch08

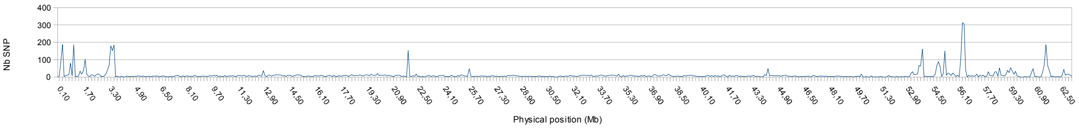

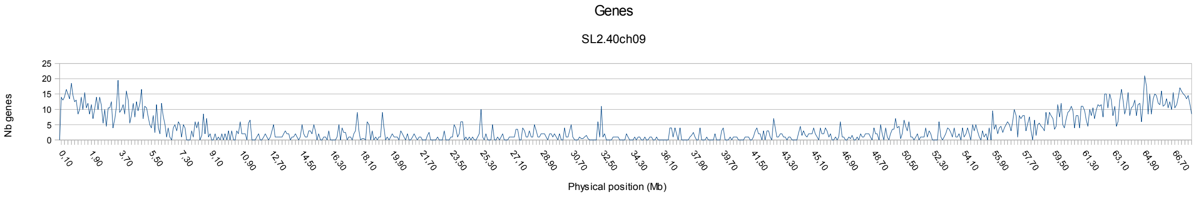
Genes
SL2.40ch09
Nb genes
25
20
15
10
5
0
0,10
1,90
3,70
5,50
7,30
9,10
10,90
12,70
14,50
16,30
18,10
19,90
21,70
23,50
25,30
27,10
28,90
30,70
32,50
34,30
36,10
37,90
39,70
41,50
43,30
45,10
46,90
48,70
50,50
52,30
54,10
55,90
57,70
59,50
61,30
63,10
64,90
66,70
Physical position (Mb)

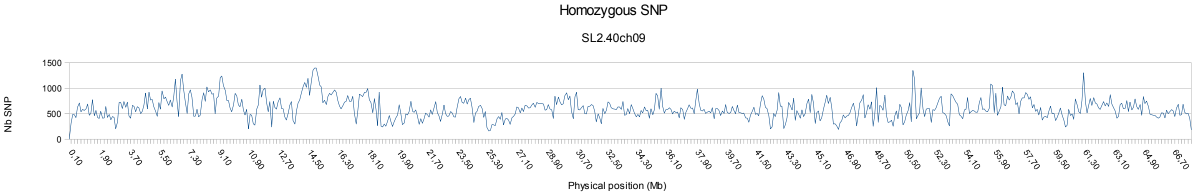
Homozygous SNP
SL2.40ch09
Nb SNP
1500
1000
500
0
0,10
1,90
3,70
5,50
7,30
9,10
10,90
12,70
14,50
16,30
18,10
19,90
21,70
23,50
25,30
27,10
28,90
30,70
32,50
34,30
36,10
37,90
39,70
41,50
43,30
45,10
46,90
48,70
50,50
52,30
54,10
55,90
57,70
59,50
61,30
63,10
64,90
66,70
Physical position (Mb)

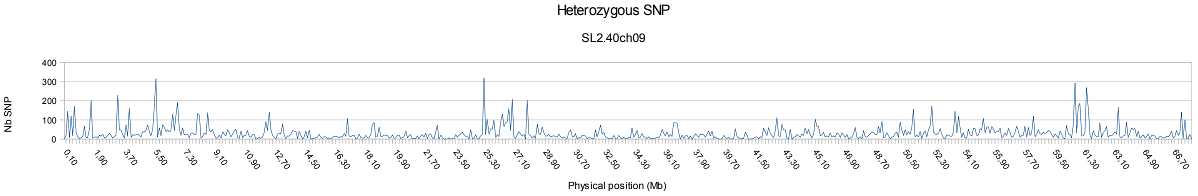
Heterozygous SNP
SL2.40ch09
Nb SNP
400
300
200
100
0
0,10
1,90
3,70
5,50
7,30
9,10
10,90
12,70
14,50
16,30
18,10
19,90
21,70
23,50
25,30
27,10
28,90
30,70
32,50
34,30
36,10
37,90
39,70
41,50
43,30
45,10
46,90
48,70
50,50
52,30
54,10
55,90
57,70
59,50
61,30
63,10
64,90
66,70
Physical position (Mb)

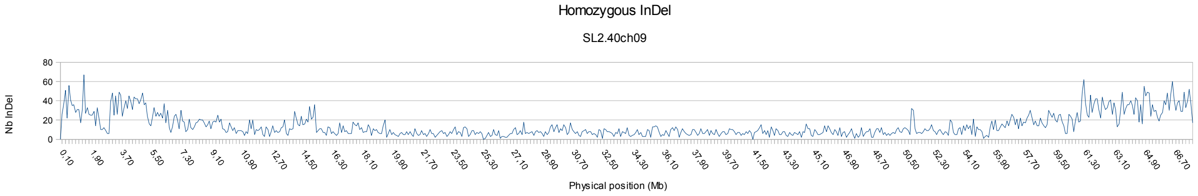
Homozygous InDel
SL2.40ch09
Nb InDel
80
60
40
20
0
0,10
1,90
3,70
5,50
7,30
9,10
10,90
12,70
14,50
16,30
18,10
19,90
21,70
23,50
25,30
27,10
28,90
30,70
32,50
34,30
36,10
37,90
39,70
41,50
43,30
45,10
46,90
48,70
50,50
52,30
54,10
55,90
57,70
59,50
61,30
63,10
64,90
66,70
Physical position (Mb)

InDel hmz

Cervil - Homozygous InDel

SL2.40ch09

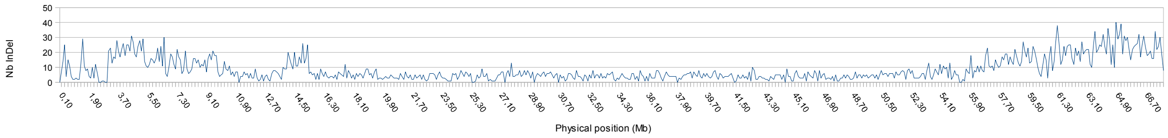

Criollo_new - Homozygous InDel

SL2.40ch09

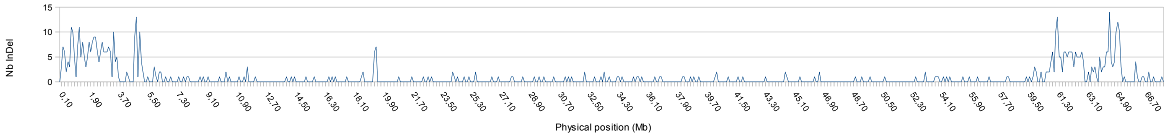

Ferum_new - Homozygous InDel

SL2.40ch09

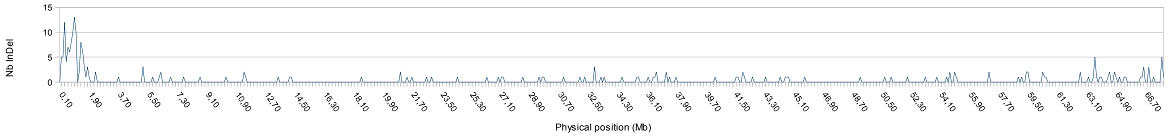

LA0147 - Homozygous InDel

SL2.40ch09

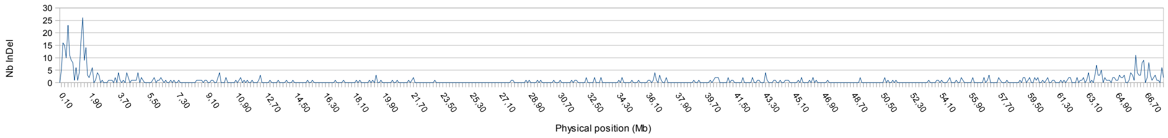

LA1420 - Homozygous InDel

SL2.40ch09

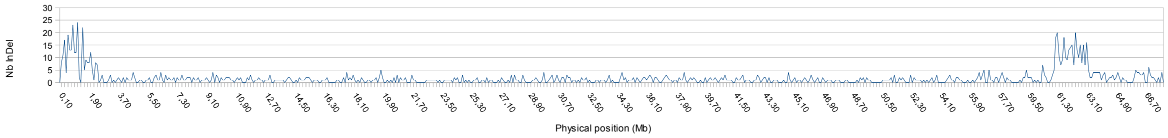

Levovil - Homozygous InDel

SL2.40ch09

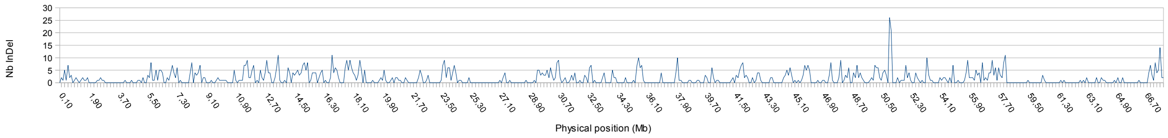

Plovdiv - Homozygous InDel

SL2.40ch09

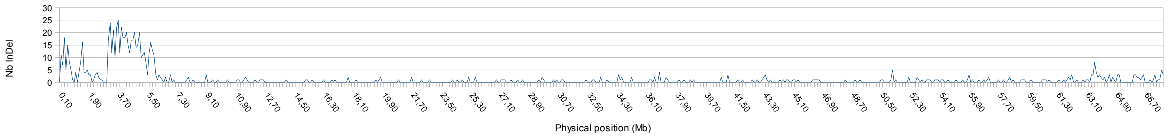

Stupicke - Homozygous InDel

SL2.40ch09

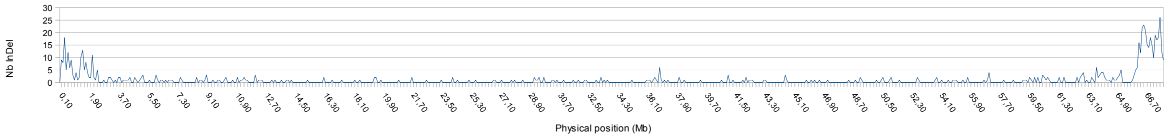

Cervil - Homozygous SNP

SL2.40ch09

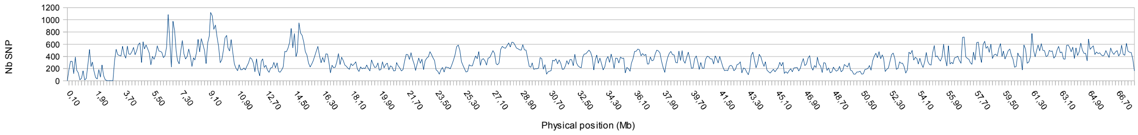

Criollo_new - Homozygous SNP

SL2.40ch09

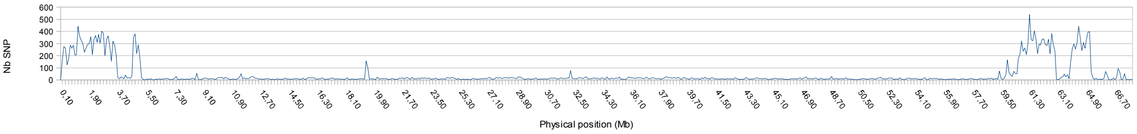

Ferum_new - Homozygous SNP

SL2.40ch09

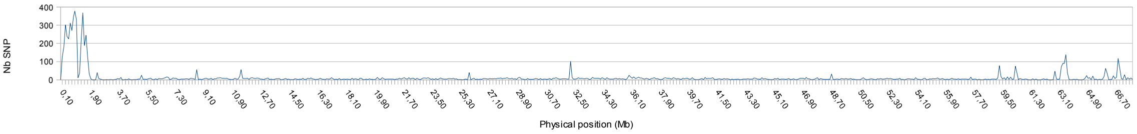

LA0147 - Homozygous SNP

SL2.40ch09

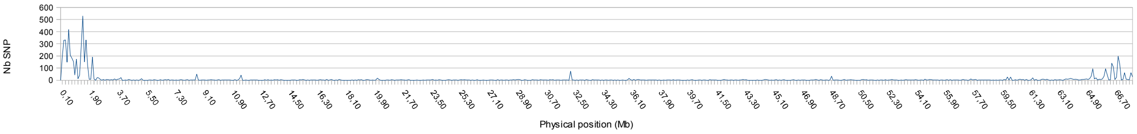

LA1420 - Homozygous SNP

SL2.40ch09

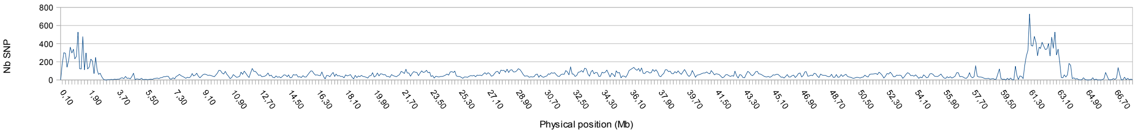

Levovil - Homozygous SNP

SL2.40ch09

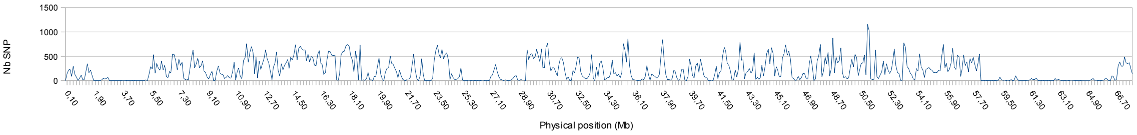

Plovdiv - Homozygous SNP

SL2.40ch09

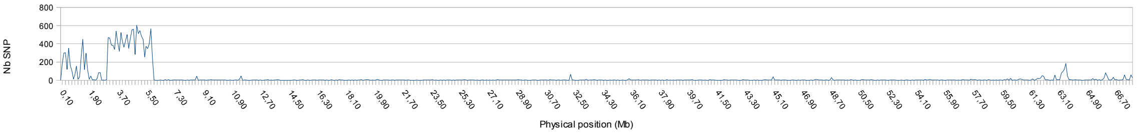

Stupicke - Homozygous SNP

SL2.40ch09

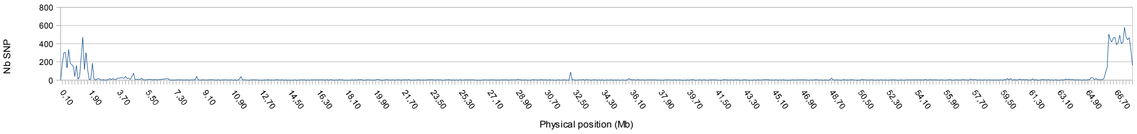

Genes

SL2.40ch10

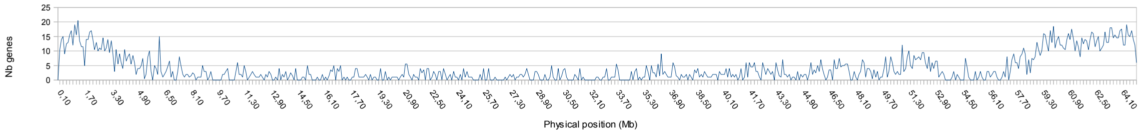

Homozygous SNP

SL2.40ch10

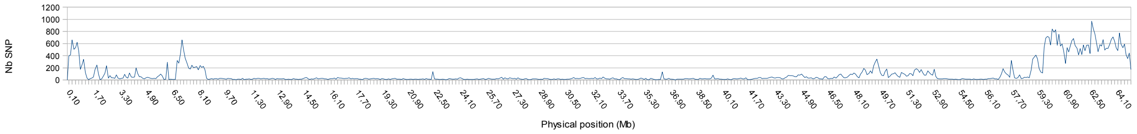

Heterozygous SNP

SL2.40ch10

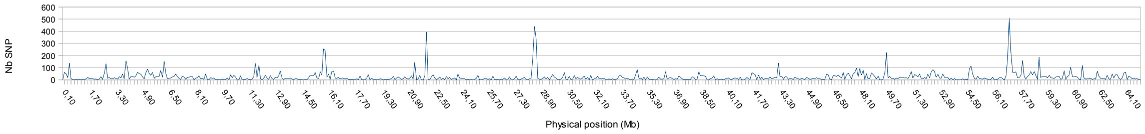

Homozygous InDel

SL2.40ch10

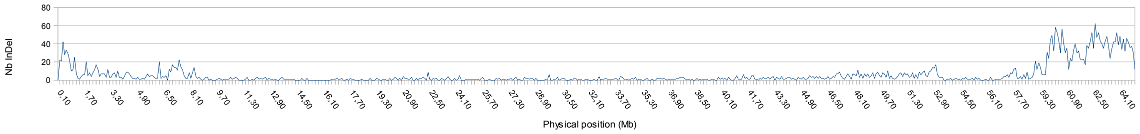

Cervil - Homozygous InDel

SL2.40ch10

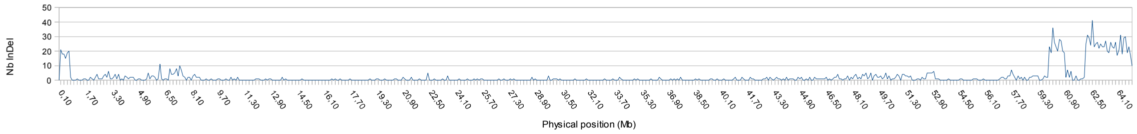

Criollo_new - Homozygous InDel

SL2.40ch10

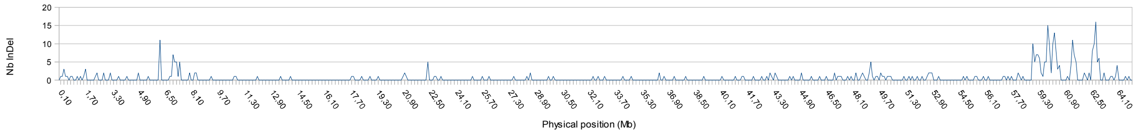

Ferum_new - Homozygous InDel

SL2.40ch10

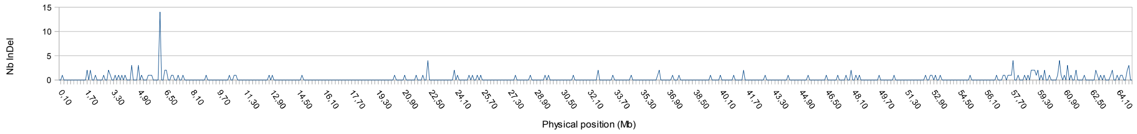

LA0147 - Homozygous InDel

SL2.40ch10

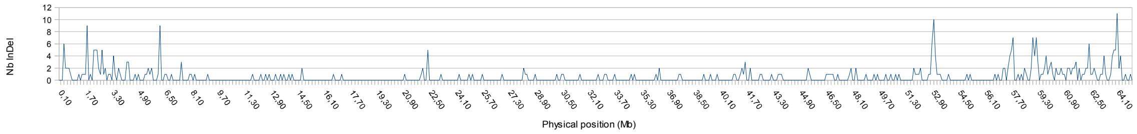

LA1420 - Homozygous InDel

SL2.40ch10

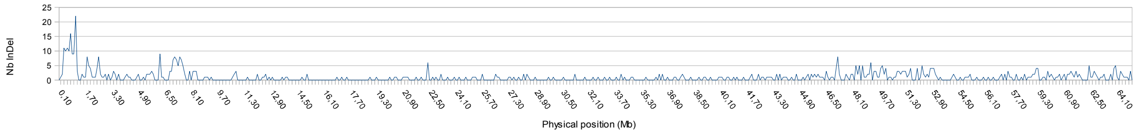

Levovil - Homozygous InDel

SL2.40ch10

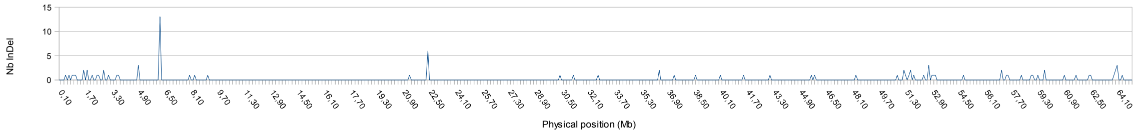

Plovdiv - Homozygous InDel

SL2.40ch10

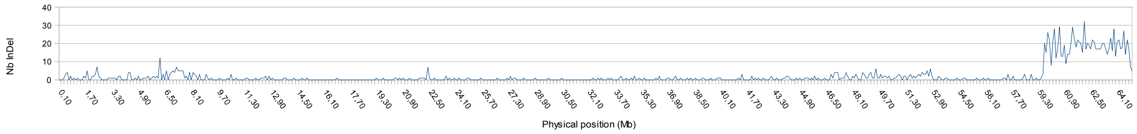

Stupicke - Homozygous InDel

SL2.40ch10

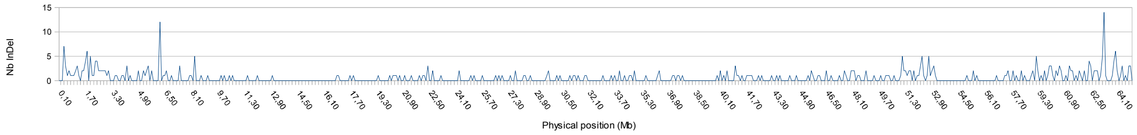

Cervil - Homozygous SNP

SL2.40ch10

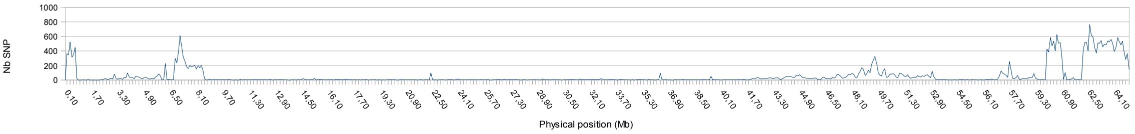

Criollo_new - Homozygous SNP

SL2.40ch10

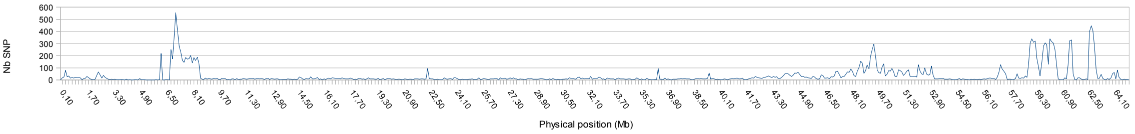

Ferum_new - Homozygous SNP

SL2.40ch10

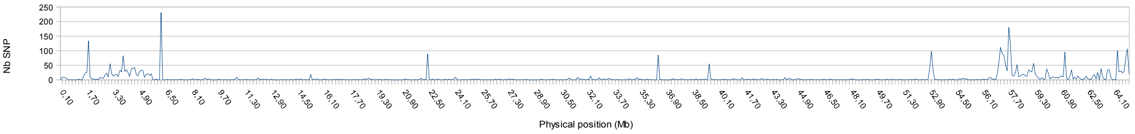

LA0147 - Homozygous SNP

SL2.40ch10

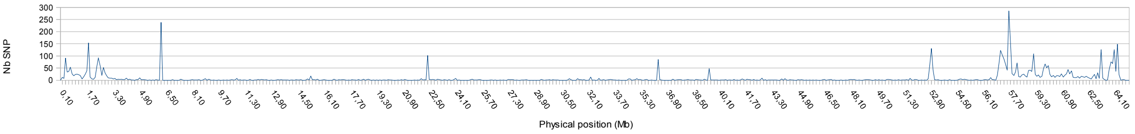

LA1420 - Homozygous SNP

SL2.40ch10

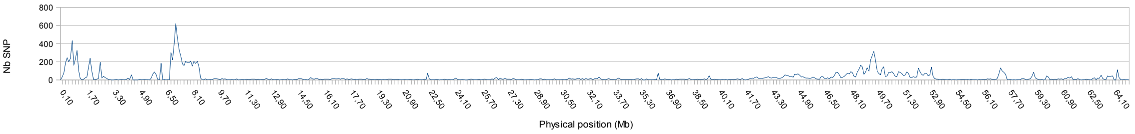

Levovil - Homozygous SNP

SL2.40ch10

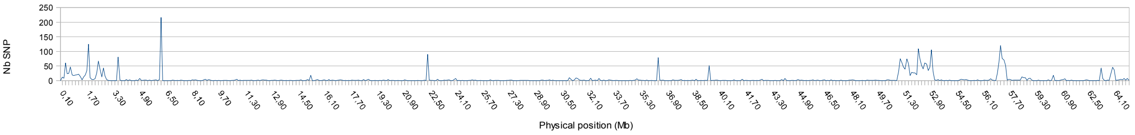

Plovdiv - Homozygous SNP

SL2.40ch10

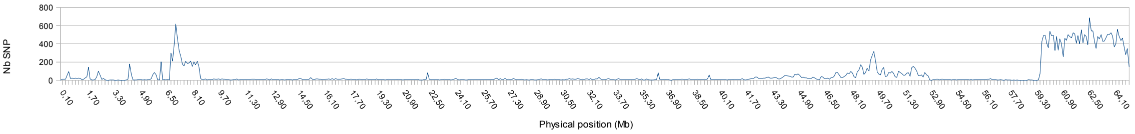

Stupicke - Homozygous SNP

SL2.40ch10

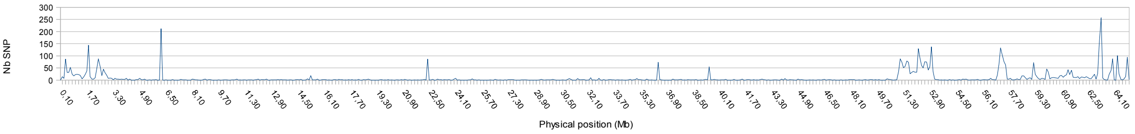

Cervil - Heterozygous SNP

SL2.40ch10

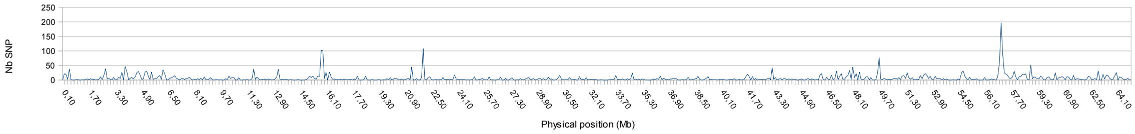

Criollo_new - Heterozygous SNP

SL2.40ch10

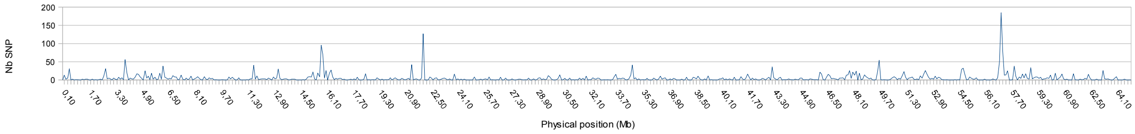

Ferum_new - Heterozygous SNP

SL2.40ch10

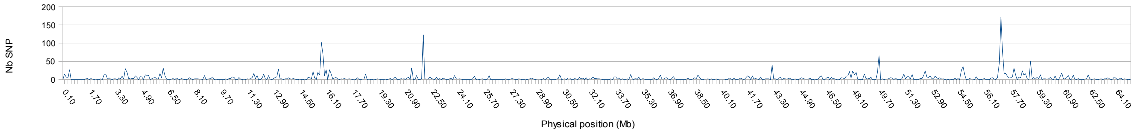

LA0147 - Heterozygous SNP

SL2.40ch10

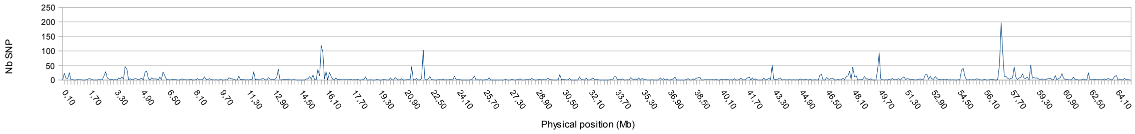

LA1420 - Heterozygous SNP

SL2.40ch10

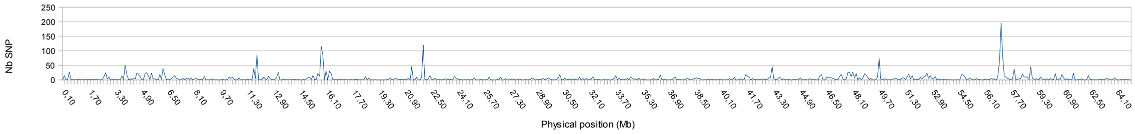

Levovil - Heterozygous SNP

SL2.40ch10

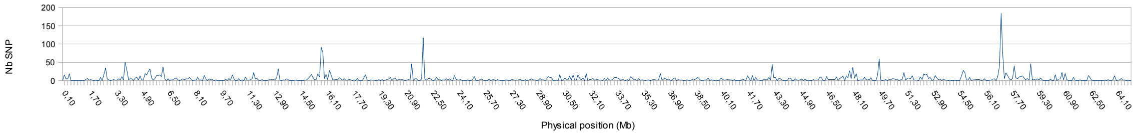

Plovdiv - Heterozygous SNP

SL2.40ch10

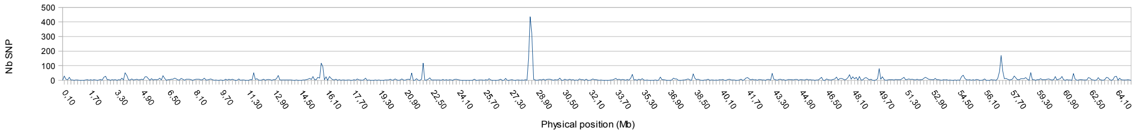

Stupicke - Heterozygous SNP

SL2.40ch10

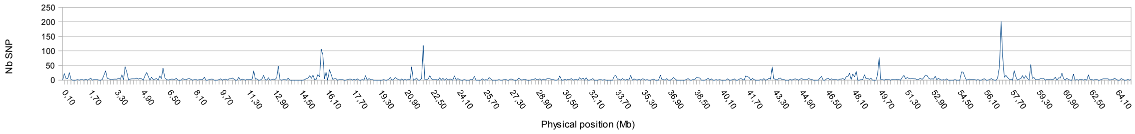

Genes

SL2.40ch11

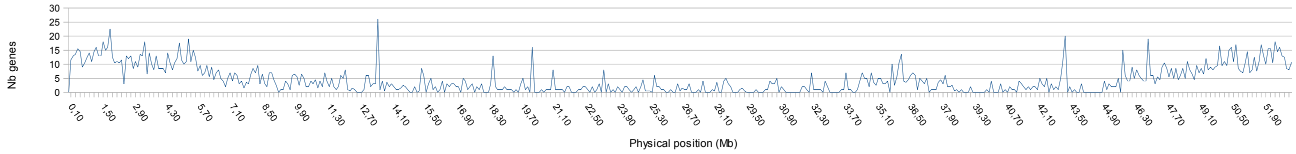

Homozygous SNP

SL2.40ch11

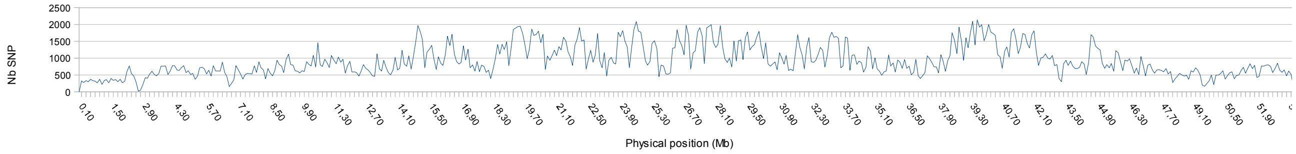

Heterozygous SNP

SL2.40ch11

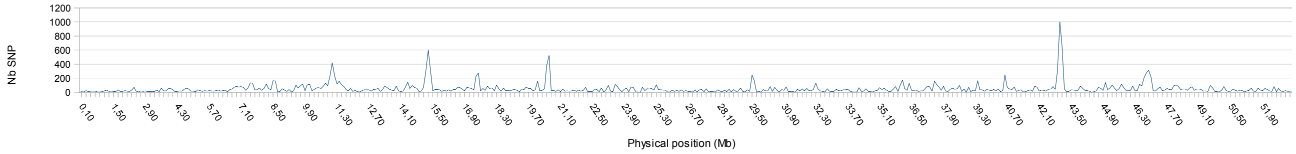

Homozygous InDel

SL2.40ch11

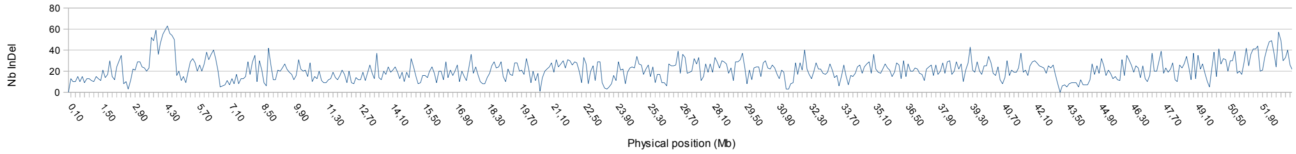

Cervil - Homozygous InDel

SL2.40ch11

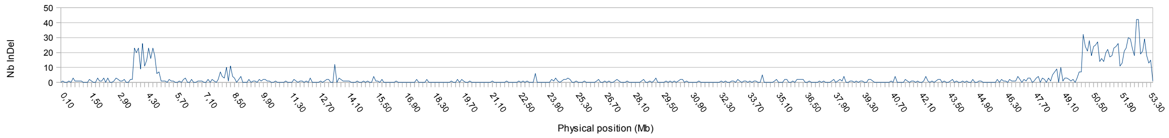

Criollo_new - Homozygous InDel

SL2.40ch11

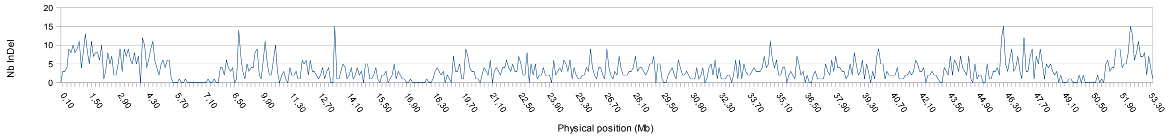

Ferum_new - Homozygous InDel

SL2.40ch11

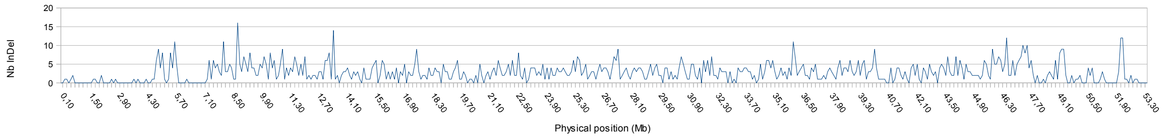

LA0147 - Homozygous InDel

SL2.40ch11

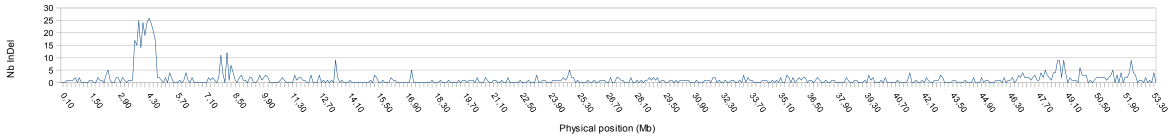

LA1420 - Homozygous InDel

SL2.40ch11

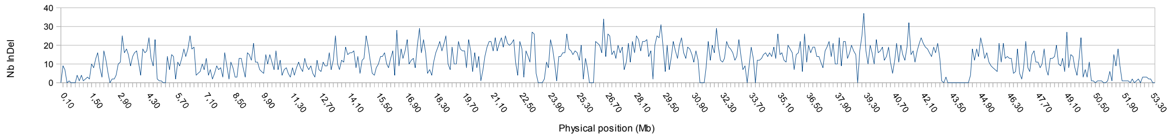

Levovil - Homozygous InDel

SL2.40ch11

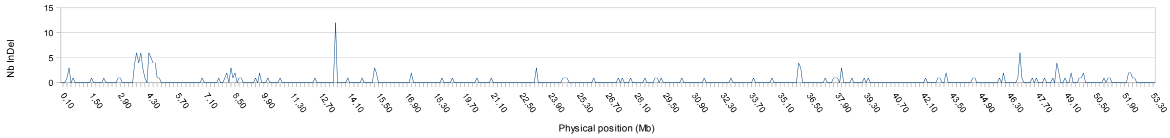

Plovdiv - Homozygous InDel

SL2.40ch11

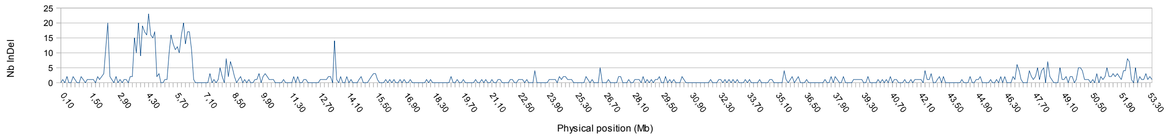

Stupicke - Homozygous InDel

SL2.40ch11

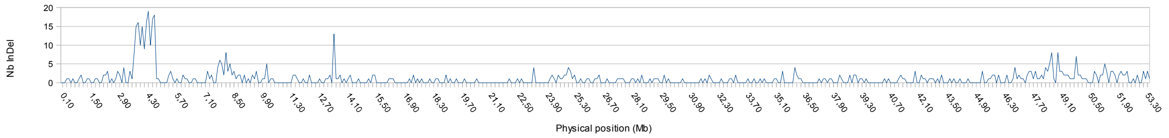

All_100kb

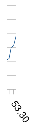

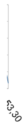

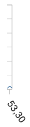

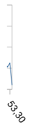

Cervil - Homozygous SNP

SL2.40ch11

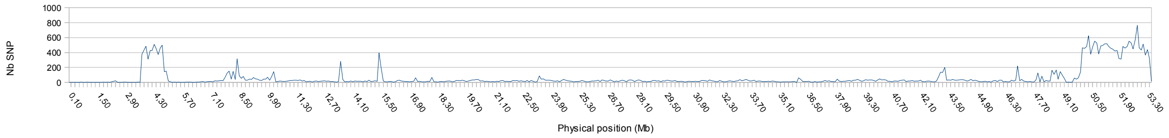

Criollo_new - Homozygous SNP

SL2.40ch11

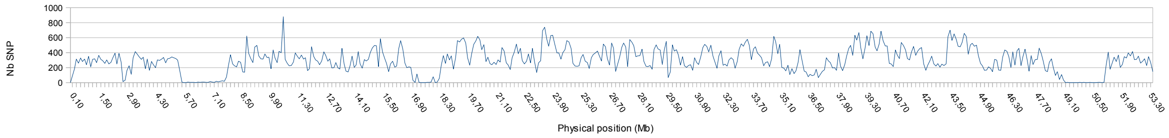

Ferum_new - Homozygous SNP

SL2.40ch11

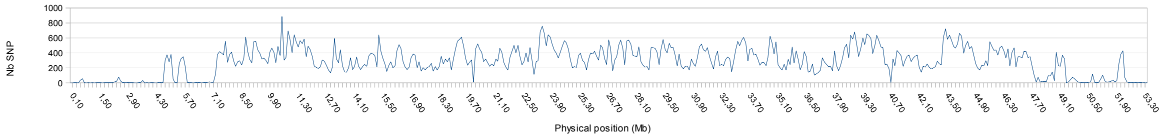

LA0147 - Homozygous SNP

SL2.40ch11

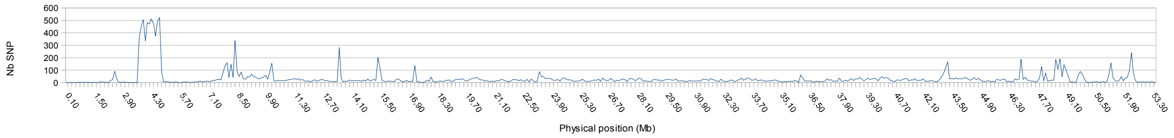

LA1420 - Homozygous SNP

SL2.40ch11

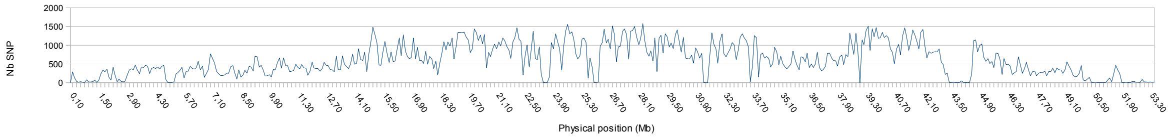

Levovil - Homozygous SNP

SL2.40ch11

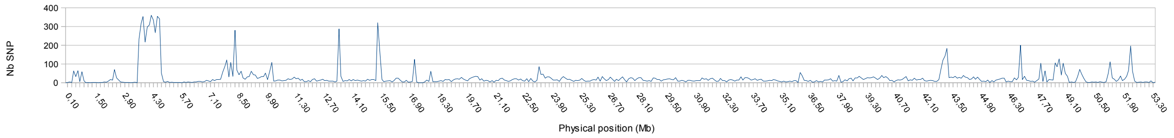

Plovdiv - Homozygous SNP

SL2.40ch11

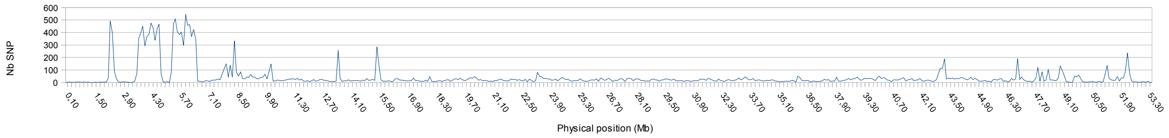

Stupicke - Homozygous SNP

SL2.40ch11

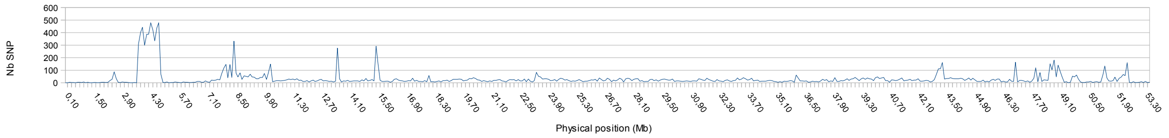

SNP htz

Cervil - Heterozygous SNP

SL2.40ch11

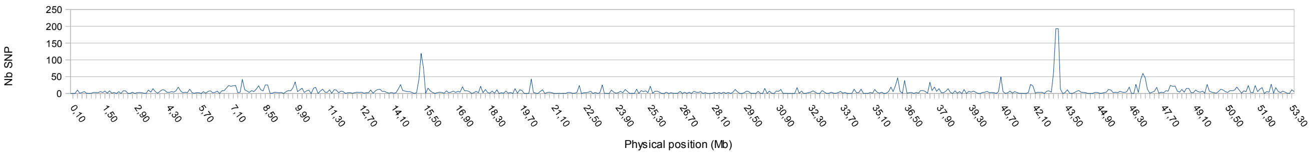

Criollo_new - Heterozygous SNP

SL2.40ch11

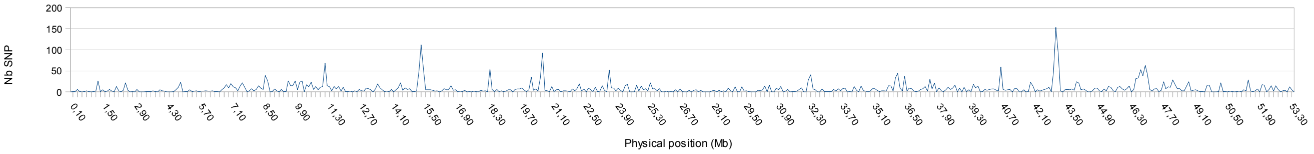

Ferum_new - Heterozygous SNP

SL2.40ch11

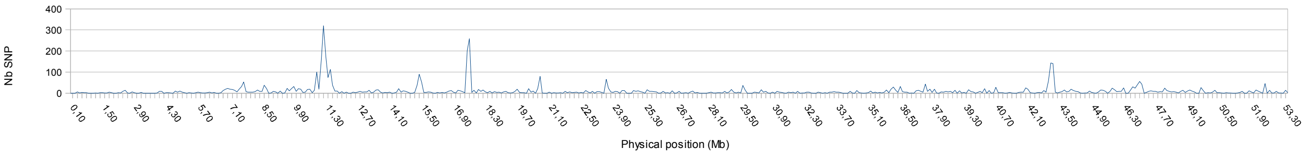

LA0147 - Heterozygous SNP

SL2.40ch11

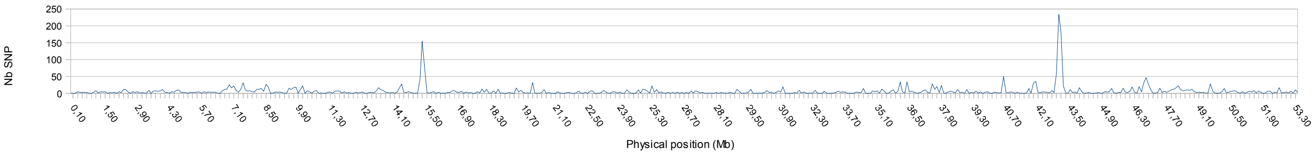

LA1420 - Heterozygous SNP

SL2.40ch11

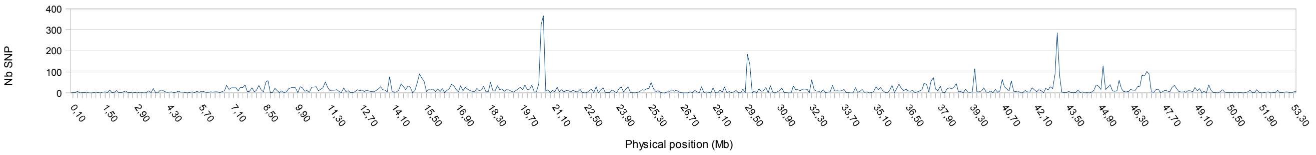

Levovil - Heterozygous SNP

SL2.40ch11

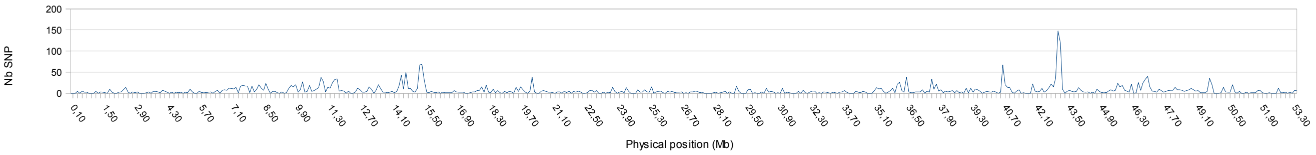

Plovdiv - Heterozygous SNP

SL2.40ch11

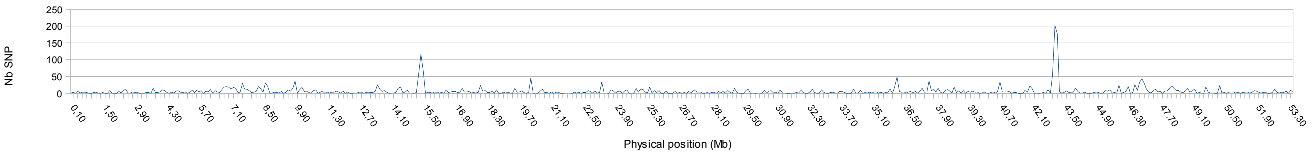

Stupicke - Heterozygous SNP

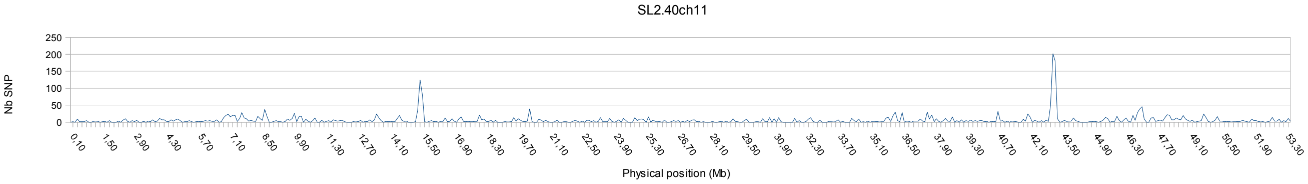
SL2.40ch11
Nb SNP
250
200
150
100
50
0
0.10
1.50
2.90
4.30
5.70
7.10
8.50
9.90
11.30
12.70
14.10
15.50
16.90
18.30
19.70
21.10
22.50
23.90
25.30
26.70
28.10
29.50
30.90
32.30
33.70
35.10
36.50
37.90
39.30
40.70
42.10
43.50
44.90
46.30
47.70
49.10
50.50
51.90
53.30
Physical position (Mb)

Genes

SL2.40ch12

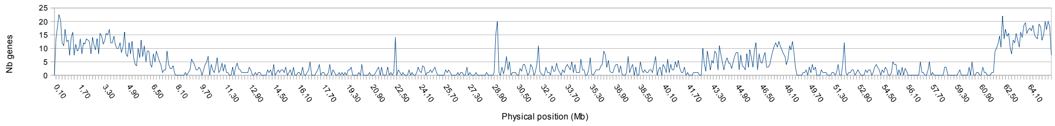

Homozygous SNP

SL2.40ch12

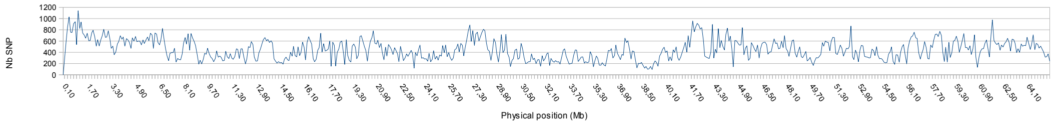

Heterozygous SNP

SL2.40ch12

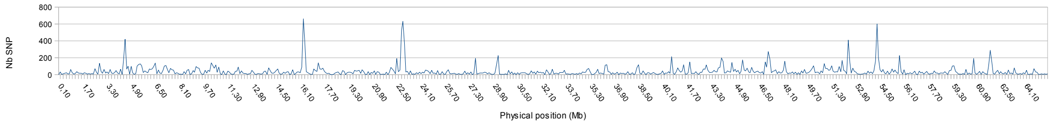

Homozygous InDel

SL2.40ch12

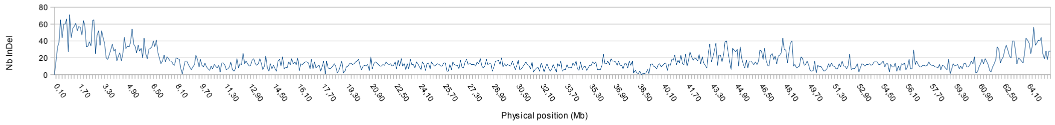

Cervil - Homozygous InDel

SL2.40ch12

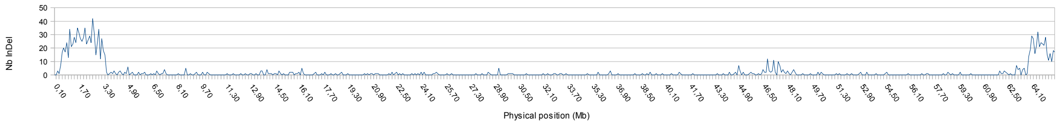

Criollo_new - Homozygous InDel

SL2.40ch12

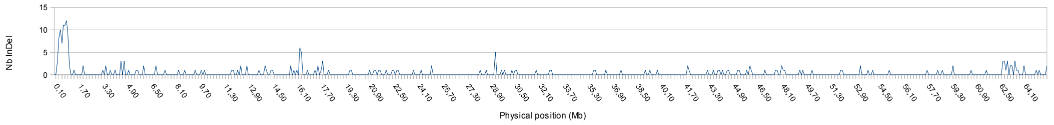

Ferum_new - Homozygous InDel

SL2.40ch12

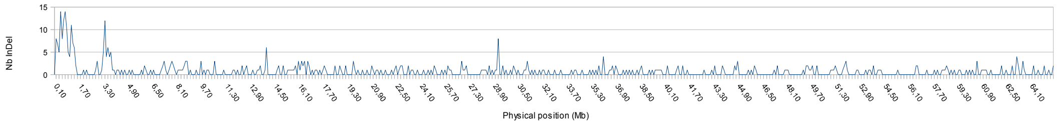

LA0147 - Homozygous InDel

SL2.40ch12

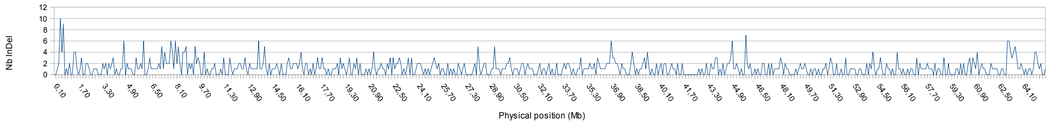

LA1420 - Homozygous InDel

SL2.40ch12

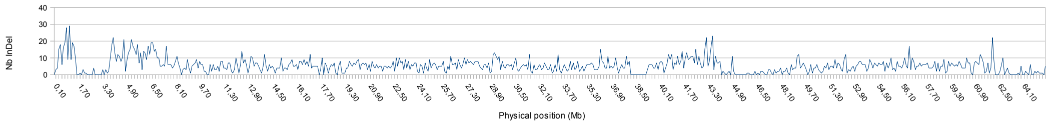

Levovil - Homozygous InDel

SL2.40ch12

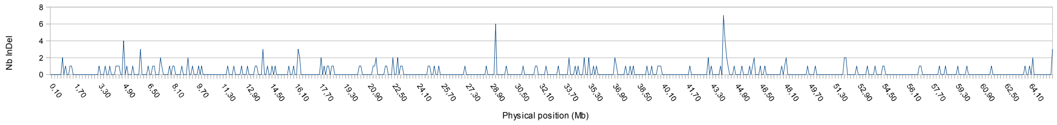

Plovdiv - Homozygous InDel

SL2.40ch12

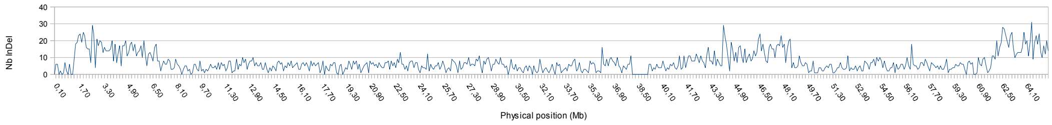

Stupicke - Homozygous InDel

SL2.40ch12

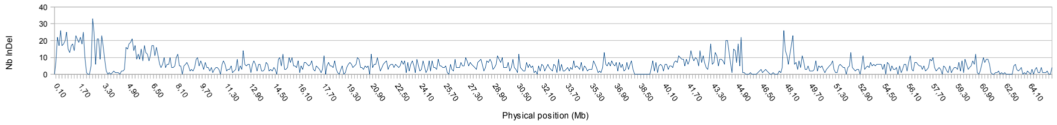

Cervil - Homozygous SNP

SL2.40ch12

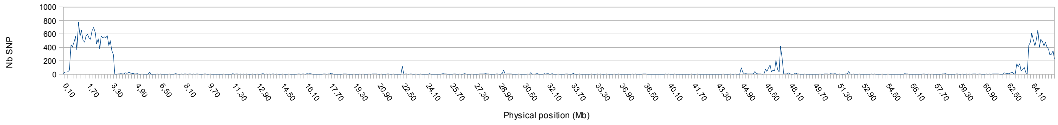

Criollo_new - Homozygous SNP

SL2.40ch12

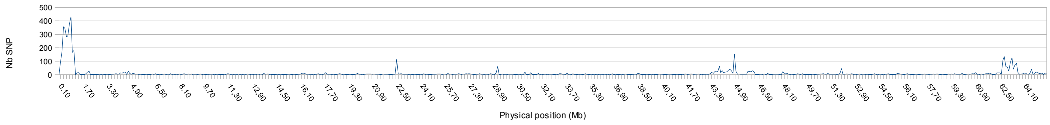

Ferum_new - Homozygous SNP

SL2.40ch12

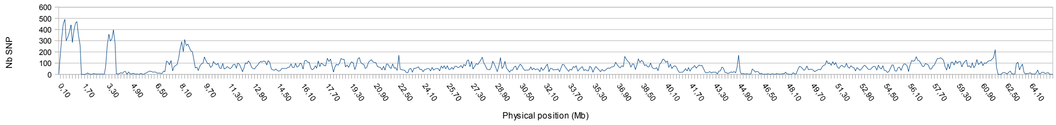

LA0147 - Homozygous SNP

SL2.40ch12

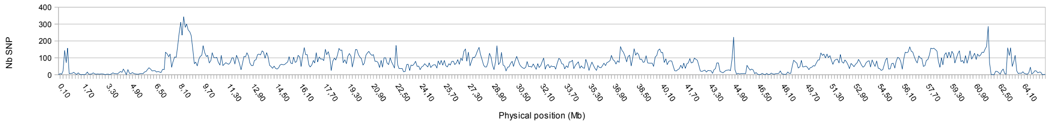

LA1420 - Homozygous SNP

SL2.40ch12

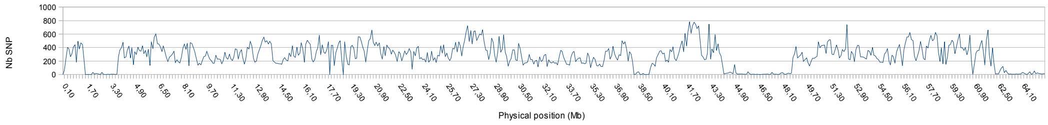

Levovil - Homozygous SNP

SL2.40ch12

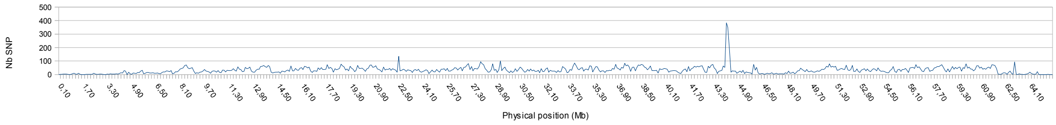

Plovdiv - Homozygous SNP

SL2.40ch12

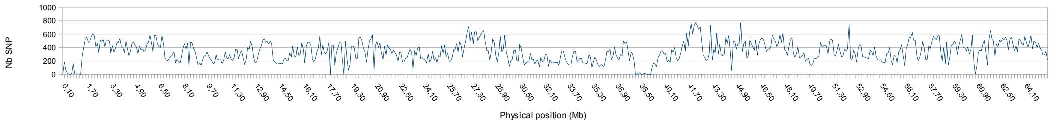

Stupicke - Homozygous SNP

SL2.40ch12

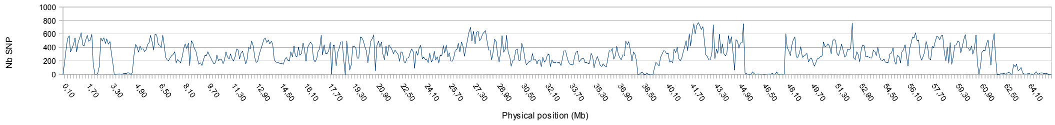

Cervil - Heterozygous SNP

SL2.40ch12

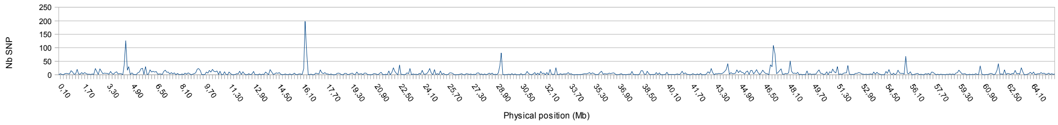

Criollo_new - Heterozygous SNP

SL2.40ch12

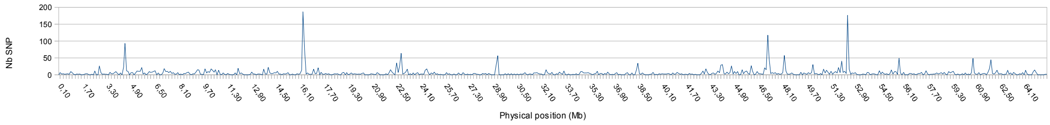

Ferum_new - Heterozygous SNP

SL2.40ch12

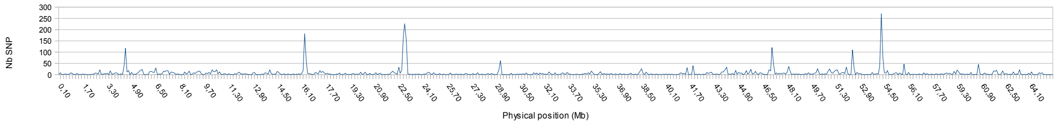

LA0147 - Heterozygous SNP

SL2.40ch12

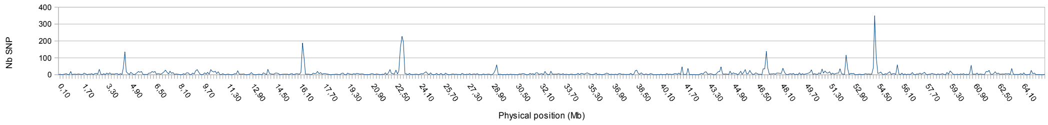

LA1420 - Heterozygous SNP

SL2.40ch12

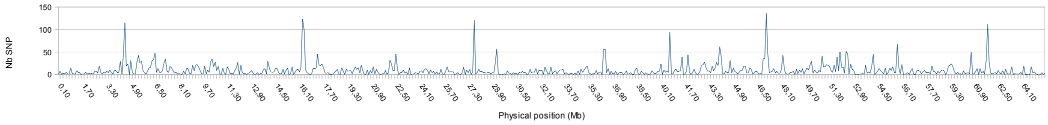

Levovil - Heterozygous SNP

SL2.40ch12

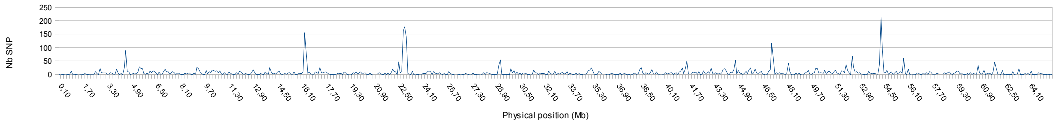

Plovdiv - Heterozygous SNP

SL2.40ch12

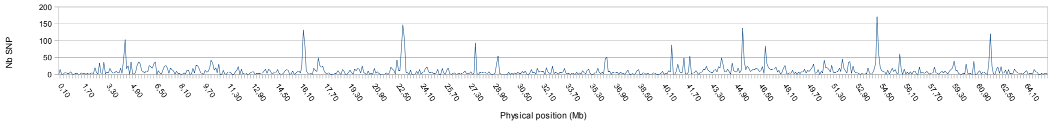

Stupicke - Heterozygous SNP

SL2.40ch12

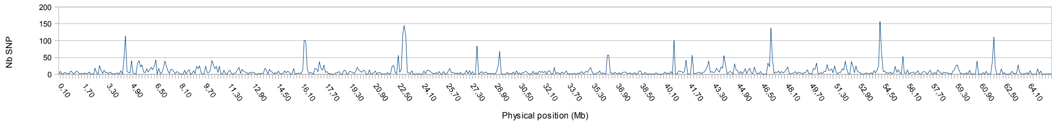

Supplement: Supplementary file 2 — Additional file 2: Figure showing the distribution of genes, homozygous and heterozygous SNPs and InDels along each chromosome over the 8 accessions and for each accession (using a window size of 100 kb). (PDF 14 MB) [file 12864_2013_5531_MOESM2_ESM.pdf]
