# Supplementary material for: Whole genome resequencing in tomato reveals variation associated with introgression and breeding events
Source: BMC Genomics. 2013 Nov 14;14(1):791. doi: 10.1186/1471-2164-14-791 (PMC4046683; doi:10.1186/1471-2164-14-791)
Supplement: Supplementary file 3 — Additional file 3: Table listing the number of homozygous InDels per chromosome and line. (DOC 52 KB) [file 12864_2013_5531_MOESM3_ESM.doc]

| **Accession** | Cervil | Plovdiv | LA1420 | Criollo | Stupicke | Ferum | Levovil | LA0147 | Total unique |
| --- | --- | --- | --- | --- | --- | --- | --- | --- | --- |
|  | *S.l.cera* | *S.l.cera* | *S.l.cera* | *S.l.cera* | *S. lyc* | *S. lyc* | *S. lyc* | *S. lyc* |  |
| **Chromosome / Totale** | 53522 | 33966 | 30927 | 13898 | 10886 | 4532 | 2894 | 7969 | 127913 |
| **ch00** | 669 | 293 | 499 | 236 | 136 | 91 | 54 | 108 | 1669 |
| **ch01** | 2624 | 1387 | 1889 | 1187 | 1100 | 313 | 290 | 1011 | 7467 |
| **ch02** | 5991 | 1856 | 1371 | 919 | 1050 | 267 | 110 | 461 | 10027 |
| **ch03** | 3026 | 5259 | 1648 | 560 | 445 | 320 | 113 | 909 | 10701 |
| **ch04** | 8216 | 6234 | 1052 | 2358 | 891 | 464 | 248 | 835 | 15835 |
| **ch05** | 8951 | 8412 | 3538 | 798 | 480 | 197 | 83 | 553 | 17956 |
| **ch06** | 3486 | 665 | 958 | 390 | 583 | 198 | 199 | 783 | 5917 |
| **ch07** | 562 | 709 | 5273 | 2973 | 495 | 159 | 91 | 446 | 8904 |
| **ch08** | 9720 | 1339 | 2765 | 1411 | 341 | 125 | 74 | 403 | 14096 |
| **ch09** | 5825 | 853 | 1202 | 627 | 701 | 233 | 1191 | 549 | 9658 |
| **ch10** | 1575 | 1434 | 660 | 391 | 394 | 161 | 93 | 369 | 3782 |
| **ch11** | 1418 | 821 | 6508 | 1808 | 592 | 1491 | 178 | 755 | 11346 |
| **ch12** | 1459 | 4704 | 3564 | 240 | 3678 | 513 | 170 | 787 | 10555 |

**Supplemental data S3: Number of homozygous InDels**

Accessions consist in four *S. lycopersicum* (S. lyc) and four cherry type (*S. l. cera*) accessions.
