# Supplementary material for: Whole genome resequencing in tomato reveals variation associated with introgression and breeding events
Source: BMC Genomics. 2013 Nov 14;14(1):791. doi: 10.1186/1471-2164-14-791 (PMC4046683; doi:10.1186/1471-2164-14-791)
Supplement: Supplementary file 4 — Additional file 4: Table listing the number of heterozygous SNPs in genomic DNA of the eight accessions (0.4> allelic frequency > 0.6). (DOC 52 KB) [file 12864_2013_5531_MOESM4_ESM.doc]

| **Accession** | Cervil | Plovdiv | LA1420 | Criollo | Stupicke | Ferum | Levovil | LA0147 | Total unique |  |
| --- | --- | --- | --- | --- | --- | --- | --- | --- | --- | --- |
|  | *S.l.cera* | *S.l.cera* | *S.l.cera* | *S.l.cera* | *S. lyc* | *S. lyc* | *S. lyc* | *S. lyc* |  |  |
| **Chromosome / Totale** | 88776 | 71391 | 70402 | 58044 | 50098 | 49218 | 49792 | 53511 | 314560 |  |
| **ch00** | 11765 | 10962 | 11168 | 10323 | 9536 | 9625 | 8527 | 10885 | 45927 |  |
| **ch01** | 7824 | 4605 | 4771 | 5616 | 6070 | 5510 | 5667 | 6913 | 26075 |  |
| **ch02** | 6634 | 3132 | 3108 | 3443 | 2668 | 2457 | 2445 | 2980 | 17499 |  |
| **ch03** | 4639 | 6435 | 3845 | 3192 | 2988 | 2960 | 3081 | 3361 | 19871 |  |
| **ch04** | 8923 | 8176 | 3144 | 4910 | 2923 | 3135 | 2927 | 2925 | 26164 |  |
| **ch05** | 9951 | 9623 | 5143 | 2716 | 2316 | 2091 | 2558 | 2479 | 27305 |  |
| **ch06** | 4219 | 3157 | 3158 | 2946 | 2806 | 2633 | 2698 | 3051 | 14194 |  |
| **ch07** | 3921 | 3550 | 8674 | 6286 | 3251 | 2907 | 3283 | 3392 | 23028 |  |
| **ch08** | 10734 | 3576 | 5453 | 4449 | 2863 | 2739 | 3258 | 3096 | 25714 |  |
| **ch09** | 7957 | 3199 | 3562 | 3061 | 2349 | 2351 | 3940 | 2620 | 21074 |  |
| **ch10** | 4252 | 4754 | 3592 | 3365 | 3137 | 2853 | 3353 | 3283 | 15822 |  |
| **ch11** | 3829 | 3239 | 8523 | 4148 | 3043 | 5254 | 3305 | 3256 | 25493 |  |
| **ch12** | 4128 | 6983 | 6261 | 3589 | 6148 | 4633 | 4750 | 5290 | 26494 |  |

**Supplemental data S4: Number of heterozygous SNP in genomic DNA of the eight accessions (0.4> allelic frequency > 0.6)**

Accessions consist in four *S. lycopersicum* (S. lyc) and four cherry type (*S. l. cera*) accessions.
