# Supplementary material for: Whole genome resequencing in tomato reveals variation associated with introgression and breeding events
Source: BMC Genomics. 2013 Nov 14;14(1):791. doi: 10.1186/1471-2164-14-791 (PMC4046683; doi:10.1186/1471-2164-14-791)
Supplement: Supplementary file 5 — Additional file 5: Figure showing the distribution of the number of heterozygous SNPs. (DOC 74 KB) [file 12864_2013_5531_MOESM5_ESM.doc]

Supplemental data S5: Distribution of the number of heterozygous SNPs in the 8 lines
