# Supplementary material for: Whole genome resequencing in tomato reveals variation associated with introgression and breeding events
Source: BMC Genomics. 2013 Nov 14;14(1):791. doi: 10.1186/1471-2164-14-791 (PMC4046683; doi:10.1186/1471-2164-14-791)
Supplement: Supplementary file 6 — Additional file 6: Table listing the classification of InDels in coding sequences according to their effects (snpEff version 2.1b). (DOC 60 KB) [file 12864_2013_5531_MOESM6_ESM.doc]

| **Accession** | Cervil | Plovdiv | LA1420 | Criollo | Stupicke | Ferum | Levovil | LA0147 |  |
| --- | --- | --- | --- | --- | --- | --- | --- | --- | --- |
|  | *S.l.cera* | *S.l.cera* | *S.l.cera* | *S.l.cera* | *S. lyc* | *S. lyc* | *S. lyc* | *S. lyc* |  |
| **HIGH** | **351** | **275** | **227** | **140** | **119** | **72** | **77** | **101** |  |
| Frame Shift | 335 | 263 | 216 | 131 | 114 | 68 | 72 | 96 |  |
| Splice Site Acceptor | 6 | 7 | 1 | 1 | 1 | 2 | 1 | 1 |  |
| Splice Site Donor | 9 | 4 | 7 | 6 | 4 | 1 | 1 | 4 |  |
| Stop Gained | 1 | 1 | 2 | 2 | 0 | 1 | 1 | 0 |  |
| Stop Lost | 0 | 0 | 1 | 0 | 0 | 0 | 0 | 0 |  |
| **MODERATE** | **75** | **52** | **55** | **36** | **20** | **11** | **6** | **10** |  |
| Codon Change Plus Codon Deletion | 17 | 10 | 14 | 12 | 4 | 3 | 1 | 2 |  |
| Codon Change Plus Codon Insertion | 11 | 11 | 5 | 7 | 7 | 1 | 1 | 2 |  |
| Codon Deletion | 20 | 18 | 21 | 9 | 7 | 3 | 2 | 3 |  |
| Codon Insertion | 27 | 13 | 15 | 8 | 2 | 4 | 2 | 3 |  |
| **MODIFIER** | **70206** | **43180** | **38400** | **19000** | **14991** | **6296** | **4037** | **11031** |  |
| Downstream | 17661 | 10252 | 8384 | 5095 | 4121 | 1762 | 1088 | 3298 |  |
| Intergenic | 25737 | 17956 | 17434 | 6178 | 4596 | 2038 | 1285 | 3032 |  |
| Intragenic | 1279 | 698 | 625 | 457 | 234 | 114 | 93 | 182 |  |
| Intron | 6672 | 4434 | 3488 | 2462 | 1653 | 729 | 496 | 1008 |  |
| Upstream | 18235 | 9802 | 8179 | 4574 | 4260 | 1602 | 1036 | 3423 |  |
| UTR 5 Prime | 153 | 89 | 77 | 54 | 39 | 13 | 6 | 31 |  |
| UTR_3_Prime | 469 | 249 | 213 | 180 | 88 | 38 | 33 | 57 |  |

**Supplemental data S6 : Classification of Indels in coding sequences according to their effects (snpEff version 2.1b)**
