# Supplementary material for: Whole genome resequencing in tomato reveals variation associated with introgression and breeding events
Source: BMC Genomics. 2013 Nov 14;14(1):791. doi: 10.1186/1471-2164-14-791 (PMC4046683; doi:10.1186/1471-2164-14-791)
Supplement: Supplementary file 7 — Additional file 7: Table listing the number of regions showing a significant copy number variant (+: excess, -: default of copy number compared to the reference genome). (DOC 54 KB) [file 12864_2013_5531_MOESM7_ESM.doc]

| chromosome | total nb excess  regions default | | Cervil | Plovdiv | LA1420 | Stupicke | LA0147 |
| --- | --- | --- | --- | --- | --- | --- | --- |
| 1 | 63 | + | 7 | 5 | 11 | 5 | 10 |
|  |  | - | 32 | 9 | 11 | 6 | 14 |
| 2 | 120 | + | 10 | 4 | 12 | 3 | 40 |
|  |  | - | 59 | 14 | 5 | 3 | 10 |
| 3 | 137 | + | 11 | 7 | 4 | 0 | 21 |
|  |  | - | 32 | 70 | 18 | 3 | 9 |
| 4 | 153 | + | 23 | 11 | 10 | 5 | 37 |
|  |  | - | 85 | 83 | 10 | 8 | 10 |
| 5 | 248 | + | 23 | 17 | 9 | 5 | 66 |
|  |  | - | 127 | 122 | 32 | 3 | 4 |
| 6 | 90 | + | 8 | 0 | 2 | 0 | 32 |
|  |  | - | 50 | 1 | 6 | 10 | 4 |
| 7 | 156 | + | 15 | 2 | 17 | 2 | 21 |
|  |  | - | 15 | 3 | 99 | 3 | 13 |
| 8 | 207 | + | 24 | 5 | 6 | 3 | 44 |
|  |  | - | 122 | 10 | 26 | 6 | 8 |
| 9 | 111 | + | 21 | 6 | 3 | 3 | 25 |
|  |  | - | 60 | 3 | 14 | 3 | 11 |
| 10 | 64 | + | 16 | 8 | 2 | 2 | 29 |
|  |  | - | 22 | 6 | 7 | 2 | 4 |
| 11 | 175 | + | 13 | 1 | 19 | 0 | 17 |
|  |  | - | 9 | 2 | 119 | 2 | 20 |
| 12 | 160 | + | 63 | 7 | 4 | 2 | 74 |
|  |  | - | 28 | 58 | 55 | 48 | 18 |

Supplemental data S7: Number of regions showing a significant copy number variant (+: excess, -: default)
