# Supplementary figures and images for: Whole genome resequencing in tomato reveals variation associated with introgression and breeding events
Source: BMC Genomics. 2013 Nov 14;14(1):791. doi: 10.1186/1471-2164-14-791 (PMC4046683; doi:10.1186/1471-2164-14-791)

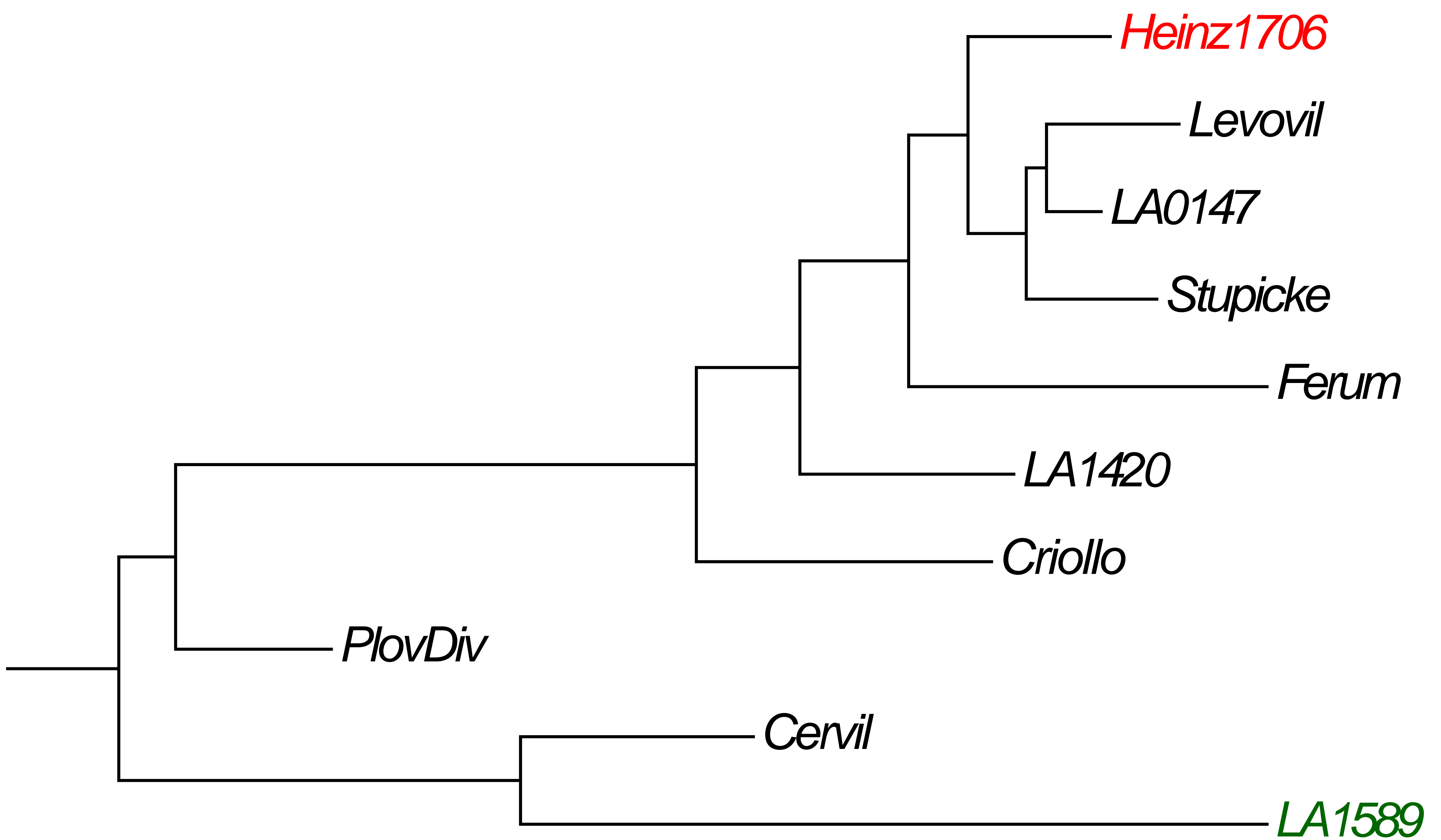

0.06

Supplement: Supplementary file 8 — Additional file 8: Phylogenetic tree representing the 8 accessions, Heinz 1706 and LA 1589, constructed with the set of 7200 SNP positions common to the SolCap array. (PDF 6 KB) [file 12864_2013_5531_MOESM8_ESM.pdf]
